# Phylogeny and Ecology of *Trebouxia* Photobionts From Bolivian Lichens

**DOI:** 10.3389/fmicb.2022.779784

**Published:** 2022-03-28

**Authors:** Magdalena Kosecka, Martin Kukwa, Agnieszka Jabłońska, Adam Flakus, Pamela Rodriguez-Flakus, Łucja Ptach, Beata Guzow-Krzemińska

**Affiliations:** ^1^Department of Plant Taxonomy and Nature Conservation, Faculty of Biology, University of Gdańsk, Gdańsk, Poland; ^2^W. Szafer Institute of Botany, Polish Academy of Sciences, Kraków, Poland

**Keywords:** biodiversity, secondary metabolites, selectivity, specificity, symbiosis

## Abstract

In the past few years, new phylogenetic lineages in *Trebouxia* were detected as a result of molecular approaches. These studies included symbiont selectivity in lichen communities, transects along altitudinal gradients at local and global scales and the photobiont diversity in local populations of lichen-forming fungal species. In most of these studies, phylogenetic and haplotype analyses based on the internal transcribed spacer (ITS) locus have continuously allowed the recognition of new monophyletic lineages, which suggests that still numerous undiscovered *Trebouxia* lineages can be hidden in lichens from unexplored areas, especially in the tropics. Here, we estimated the biodiversity of photobionts in Bolivian Andean vegetation and assessed their specificity. About 403 lichen samples representing 42 genera, e.g., *Haematomma*, *Heterodermia*, *Hypotrachyna*, *Lecanora*, *Lepra*, *Leucodermia*, *Parmotrema*, *Pertusaria*, *Polyblastidium*, and *Usnea*, containing *Trebouxia* photobionts, were analyzed. ITS ribosomal DNA (rDNA) and *rbc*L markers were used. We obtained *Trebouxia* sequences from Bolivian samples belonging to already described clades A, C, I, and S. Thirty-nine *Trebouxia* lineages were distinguished within these clades, while 16 were new. To reveal the structure of the community of Bolivian photobionts and their relationships with mycobionts, the comparative effects of climate, altitude, geographical distances, substrate, and habitat type, as well as functional traits of lichens such as growth forms, propagation mode and secondary metabolites, were analyzed. Furthermore, new Bolivian records were included in analysis on a global scale. In our study, the mycobiont genus or even species are the most important factors correlated with photobiont identity. Moreover, we revealed that the community of Bolivian photobionts is shaped by altitude.

## Introduction

Lichens are globally distributed symbiotic associations formed by fungi (mycobionts) with autotrophic partners (photobionts), which are mainly represented by eukaryotic green algae (mostly belonging to genera *Asterochloris*, *Trebouxia*, and *Trentepohlia*) and more rarely by cyanobacteria (mainly *Nostoc*). Their thalli are also inhabited by bacteria and other fungal species, including endolichenic fungi (Friedl and Büdel, 2008; Nash, 2008; Muggia and Grube, 2018). The distribution of lichens and their habitat preferences are shaped by the requirements of all symbionts forming the holobiont (Vančurová et al., 2018), and, according to this hypothesis, symbionts allow their hosts to live in habitats from which they would be otherwise excluded. Furthermore, hosts show huge variation in their dependence on symbiotic partners (Peksa and Škaloud, 2011) or *vice versa*—symbiotic partners vary in their specificity and interaction with the host or the whole symbiotic system (Muggia and Grube, 2018).

About 90% of the lichen-forming fungal species (mycobionts) are associated with green photobionts belonging to Chlorophyta (Tschermak-Woess, 1988, 1989), with *Trebouxia* being one of the most common genera of coccoid algae found in lichen thalli (Ahmadjian, 2001; Muggia et al., 2020). The representatives of this genus are associated with lichen species of different growth forms and from different habitats, and, according to some studies, *Trebouxia* does not necessarily require lichenization and may frequently occur also as free-living (Mukhtar et al., 1994; Ettl and Gärtner, 1995). The representatives of the genus have also been detected in different environmental samples (Sanders, 2005; Handa et al., 2007; Hallmann et al., 2013; Yung et al., 2014); however, it is not certain if lichenized diaspores were present as admixture in those samples.

The application of diverse molecular approaches over the past few years has led to a better understanding of the relationships and species diversity within *Trebouxia*, and nowadays, the determination of species or OTUs (operational taxonomic units) using molecular data has become faster and therefore preferred over phenotypic studies with cultures (e.g., Beck, 1999; Helms et al., 2001; Blaha et al., 2006; Voytsekhovich and Beck, 2015; Molins et al., 2018; Muggia et al., 2020). *Trebouxia* is thus now one of the best studied lichen photobionts, but the knowledge on their diversity and specificity to lichen mycobionts, especially in tropical regions, is far from being satisfactory.

In the past few years, new phylogenetic lineages in *Trebouxia* were identified by molecular phylogenetic methods. These studies focused on symbiont selectivity in the lichen communities of typically lichen-rich ecosystems such as in Antarctic regions (Pérez-Ortega et al., 2012; Ruprecht et al., 2012) or alpine habitats (Leavitt et al., 2013; Muggia et al., 2014b). Moreover, transects along altitudinal gradients at local and global scales and the photobiont diversity in the local populations of lichen-forming fungal species were analyzed (e.g., Werth and Sork, 2010, 2014; Vargas-Castillo and Beck, 2012; Werth, 2012; Dal Grande et al., 2017). Studies were also conducted on globally distributed lichens (e.g., Fernandez-Mendoza et al., 2011; Domaschke et al., 2012; Muggia et al., 2014a,b, 2020). In most of these studies, phylogenetic and haplotype analyses based on a single ITS locus (which is proposed as a candidate for DNA-barcoding approaches for these algae) suggested the recognition of new monophyletic lineages, which showed that still numerous undiscovered *Trebouxia* lineages may be hidden in lichens occurring in poorly explored areas, especially in the tropics.

Interactions between potential lichen symbionts are frequently described in terms of availability, selectivity, and specificity. However, there are several definitions of these terms. In the approach proposed by Beck et al. (2002) selectivity is the range of potential partners selected by the mycobiont. Five levels of selectivity were distinguished: very low, low, intermediate, high, and very high. On the other hand, the term specificity refers to the concept of a symbiotic relationship in which the selectivity is very high, and an exclusive interaction occurs. In another view, from the perspective of lichen-forming fungi, specificity can be defined as the range of compatible photobiont partners, while selectivity is used to describe the frequency of associations with distinct photobiont lineages (Yahr et al., 2004). The patterns of associations can therefore be described by the degree of specificity and selectivity. It was suggested that mycobionts may be qualitatively characterized as photobiont specialists, intermediates, and generalists (Rambold et al., 1998; Yahr et al., 2004). Specialists are restricted to a single lineage or species of *Trebouxia*, whereas generalists can be associated with several lineages of *Trebouxia*. In this paper, we follow the definitions provided by Yahr et al. (2004).

Here, we estimate the biodiversity of *Trebouxia* photobionts in Bolivian Andean vegetation, taking into account their specificity in the context of global data. We aim to understand and reveal the global spatial distribution, develop hypotheses about the adaptation strategies of *Trebouxia* algae, and study how their diversity is influenced by various factors, including: mycobiont hosts, morphological traits of thalli (growth form and type of reproduction) climatic conditions, altitude, spatial distribution, and the composition of secondary metabolites in lichen thalli.

## Materials and Methods

### Materials

Four hundred three lichen samples containing *Trebouxia* photobionts were studied. Lichens were collected randomly from various habitats and substrata. The majority of samples were obtained not only from the Yungas forest and Tucuman-Bolivian forest but also from the dry inter-Andean forest. Lichen samples were collected in Bolivia with the permission of Ministerio de Media Ambiente y Agua (MMAYA/VMABCC GDF/DGBAP/MEG No 03272/2016) and in cooperation with Herbario Nacional de Bolivia (LPB), which, in turn, made specimens available to the herbarium of University of Gdańsk (UGDA) and Polish Academy of Sciences (KRAM). Original samples are deposited in herbarium LPB, with duplicates stored in KRAM and UGDA. The determination of lichen species was conducted with appropriate literature and identification keys (e.g., Hale, 1965; Elix and Nash, 1992; Kashiwadani and Kalb, 1993; Sipman et al., 2009; Truong et al., 2011, 2013; Guzow-Krzemińska et al., 2019). Morphology and secondary metabolites were studied for each specimen. For several *Usnea* spp. and all sterile crustose lichen samples, the sequencing of the nuclear internal transcribed spacer (nucITS) rDNA of mycobiont was performed to facilitate the determination and, in the case of sterile samples, to assign the sample to the appropriate genus. Lichen secondary metabolites were identified with thin-layer chromatography (TLC) in solvents A, B, and/or C (Orange et al., 2001). Detailed data are presented in Supplementary Table 1. A map with precise localities is available at: https://www.google.com/maps/d/edit?mid=17VHEu0_jywTPdrGKaHG8CRhSDvB76dBH&usp=sharing.

### Methods

#### Molecular Methods

Well-preserved specimens lacking any visible symptoms of fungal infection were used for DNA isolation (total lichen DNA), following the plant protocol for the Plant and Fungi DNA Purification Kit (EURx, Gdańsk, Poland), with the incubation step extended to 1 h. The algal nuclear internal transcribed spacer (ITS, ITS1-5.8S-ITS2) (Kroken and Taylor, 2000; Helms et al., 2001; Guzow-Krzemińska, 2006) and, in a few cases of unique lineages of *Trebouxia*, also *rbc*L gene fragment (Nozaki et al., 1995; Nelsen et al., 2011) were amplified. For several *Usnea* spp. and sterile crustose lichen samples, the amplification and sequencing of the nucITS rDNA of mycobiont was performed using primers ITS1F and ITS4A (Gardes and Bruns, 1993; Kroken and Taylor, 2001). The PCR condition is presented in Supplementary Table 2. Sequencing was performed in Macrogen^®^ (Amsterdam, Netherlands) using amplification primers. Sequences were compared to those available in GenBank using Megablast searches (Altschul et al., 1990) to verify their identity and detect potential contaminants. The newly obtained sequences of the ITS rDNA and fragments of the *rbc*L gene were deposited in GenBank (Supplementary Table 1).

#### Phylogenetic Analysis

We conducted phylogenetic analysis separately for each of described *Trebouxia* clades A, C, I, and S to ascertain the position of our sequences within these clades. Clade A alignment included 2,152 sites and contained 221 sequences, 33 of which were newly generated. The clade C alignment included 1,562 sites and contained 295 sequences, 224 of which were newly obtained, while the clade I alignment included 1,552 sites and contained 229 sequences, 149 of which were newly generated here. Finally, the clade S alignment included 1,919 sites and contained 72 sequences, 5 of which were newly obtained sequences. The alignments for clades A and S included *cox*2 (cyclooxygenase) reference sequences (Muggia et al., 2020). About 406 previously published *Trebouxia* sequences were used as references, and represent OTUs distinguished in the latest review paper by Muggia et al. (2020), together with additional newly described OTUs (Molins et al., 2018; Škaloud et al., 2018; Moya et al., 2021; Supplementary Table 3). Sequence alignments were performed using multiple alignment using fast fourier transform (MAFFT) with automatically selected parameters—multiple alignment using fast Fourier transform (Katoh et al., 2002) as implemented in UGENE (Okonechnikov et al., 2012). The best fit evolutionary model was selected for concatenated, ITS rDNA, *rbc*L, and *cox*2 datasets, based on Akaike information criterion as implemented in MrModeltest 2.0 (Nylander, 2004). The *Trebouxia* sequence datasets were analyzed as a concatenated dataset of three loci with the GTR + I + G model selected.

Phylogenetic relationships were inferred with Bayesian inference carried out in MrBayes v.3.2.2 (Huelsenbeck and Ronquist, 2001). In cases where *rbc*L sequences were lacking, they were treated as missing data. Two parallel MCMC runs were performed, using four independent heated chains and 12 million generations, sampling every 1,000th tree. The initial 3,000 trees of each run (25%) were discarded as burn-in, and posterior probabilities were estimated by constructing a majority-rule consensus tree of all sampled post-burn in trees. Maximum likelihood (ML) analysis was performed using the edge-linked partition model in *IQ-TREE* (Nguyen et al., 2015; Chernomor et al., 2016) with 10,000 bootstrap replications on the CIPRES Science Gateway (Miller et al., 2010).

The tree topology obtained by the ML method did not contradict the Bayesian tree; therefore, only the Bayesian tree is shown. The consensus trees were visualized using FigTree v1.4.2 (Rambaut, 2006–2014). Branches with bootstrap support ≥ 70% and posterior probabilities ≥ 0.95 were considered to be strongly supported.

#### Statistical Analysis

The comparative effects of selected variables were analyzed by variation partitioning in the redundancy analysis described in Kosecka et al. (2021). To reveal the structure of the Bolivian photobiont pattern, we analyzed its relationship with the lichen genus or family and assessed the comparative effects of climate, altitude, geographical distances, substrate, and habitat type, as well as thallus type, propagation mode, and secondary metabolites composition. Variation partitioning analysis was carried on the ITS database separately for all Bolivian data obtained in this study (*N* = 408 and additional four from Garrido-Benavent et al., 2020). Additionally, using all *Trebouxia* sequences available in GenBank, we applied several variation partitioning analyses on different datasets: all available data (*N* = 2,880), Clade A (*N* = 1,080), Clade C (*N* = 347), Clade I (*N* = 381), and Clade S (*N* = 1,072). In the case of data obtained from GenBank, only records with precise geospatial coordinates and species affiliation were used. Furthermore, we filtered those data by selecting one representative haplotype of *Trebouxia* for species of mycobiont for each locality, to avoid homogeneity in the dataset. For those datasets, we applied a series of analysis, using mycobiont host as an explanatory variable together with climate, altitude, and geographical distance. To reveal any relationship on different taxonomic ranks, we coded mycobiont host as a genus and family in subsequent series of variation partitioning analysis. As secondary metabolites composition is correlated with the taxonomic affiliation of lichens, we assessed the presence or absence of identified groups of metabolites separately as an explanatory variable with remaining factors. For the Bolivian dataset, we also applied habitat type, thalli forms, propagation mode, and substrate as variables. In our sampling, certain genera of lichens were more abundant; therefore, we prepared datasets supplemented by GenBank data for *Heterodermia* (*N* = 21), *Hypotrachyna* (*N* = 60), *Lecanora* (*N* = 140), *Lecidea* (*N* = 100), *Lepra* (*N* = 48), *Parmotrema* (*N* = 137), *Pertusaria* (*N* = 33), *Polyblastidium* (*N* = 28), *Usnea* (*N* = 51), and *Xanthoparmelia* (*N* = 431). For those datasets, we conducted a series of variation partitioning analysis, using mycobiont host species and secondary metabolites as an explanatory variable together with climate and geographical distance. Distance-based redundancy analysis (dbRDA) (McArdle and Anderson, 2001) was used to select statistically significant predictors for explaining variation for each of the datasets used in the variation partitioning analysis (Supplementary Table 5). We selected 15 groups of lichens’ secondary metabolites: A—no substances, B—aliphatic (fatty) acids, C—anthraquinones, D—ergochromes, E—depsones, F—orcinol depsides, G—β-orcinol depsides, H—orcinol depsidones, I—β-orcinol depsidones, J—orcinol tridepsides, K—pulvinic acid derivatives, L—terpenoids, M—usnic acid derivatives, N—xanthones, and O—pigments. We coded secondary metabolites based on the presence or absence of particular groups of substances detected by TLC. We treated orcinol depsides, β-orcinol depsides, pulvinic acid derivatives, and usnic acid derivatives as substances that show an allelopathic or toxic impact on photobionts, based on the research of Bačkor et al. (1998, 2010a) and Lokajová et al. (2014). No substances, anthraquinones, ergochromes, orcinol depsidones, β-orcinol depsidones, orcinol tridepsides, and pigments were recognized as groups of compounds neutral for photobionts and in most cases, even protecting them against the negative effects of UV radiation (Bačkor et al., 1998, 2010a; Lokajová et al., 2014; Goga et al., 2018). Unfortunately, the remaining groups of compounds cannot be fully interpreted here due to the lack of information about their influence on the cells of green algae. If the substance could not be identified and received the status “unknown,” it was not considered in further analysis. Most publications do not provide the results of TLC, and for such records, we assumed the composition of secondary metabolites according to the generally available knowledge, disregarding intraspecific chemical variability (Supplementary Tables 6–10).

To detect and visualize the differential ordination (tendency or strategies) of data in the hyperspace, we performed principal component analysis (PCA), considering the climatic factors (BIO1–BIO19) and the composition of secondary metabolites grouping results depending on the taxonomic affiliation to the *Trebouxia* clades defined by Muggia et al. (2020). In the case analysis of separate clades, we divided them into groups based on taxonomic affiliation and phylogenetic similarity. In addition, we performed these analysis for Bolivian samples, grouping the results by habitat type. All analyses were performed in R v 3.6.0 (R Core Team, 2017), using RStudio v.1.2.1335 (RStudio Team, 2018).

## Results

### Phylogenetic Analysis

In this study, from 403 lichen samples representing 42 genera (e.g., *Haematomma*, *Heterodermia*, *Hypotrachyna*, *Lecanora*, *Lepra*, *Leucodermia*, *Parmotrema*, *Pertusaria*, *Polyblastidium*, and *Usnea*), containing *Trebouxia* photobionts, we generated 411 new ITS rDNA (OK533683 – OK534091) sequences and 94 sequences of the chloroplast *rb*cL gene fragment (OK492060 – OK492154) (Supplementary Table 1). Altogether, we recovered 117 lineages, 101 of which have been previously identified (Molins et al., 2018; Škaloud et al., 2018; Muggia et al., 2020; Medeiros et al., 2021; Moya et al., 2021), and for the new ones, we applied the same scheme of nomenclature as in previous papers.

**FIGURE 1:**
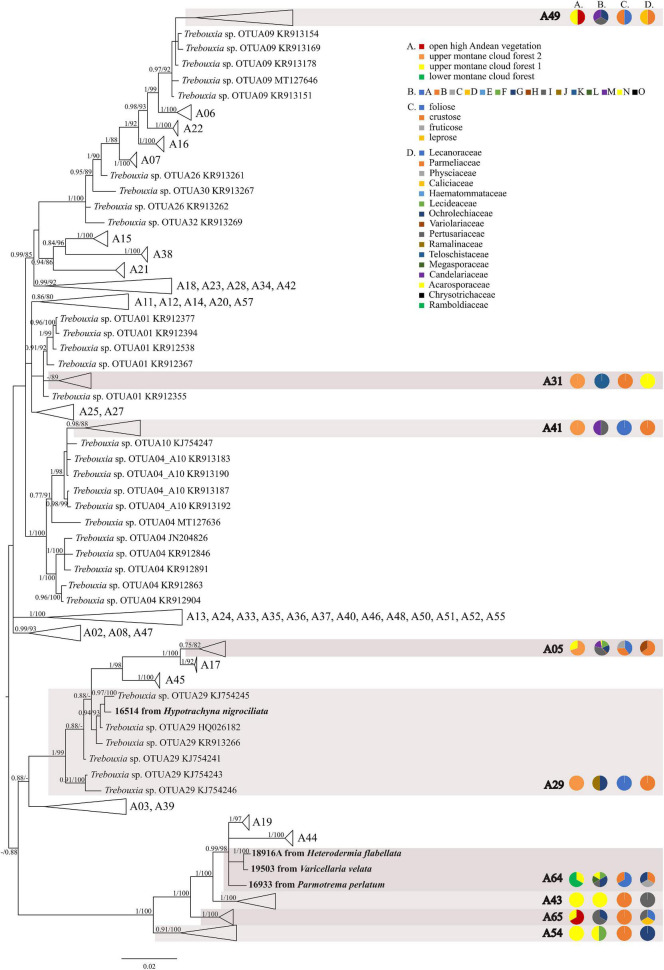
Collapsed majority-rule consensus tree from Bayesian analysis of *Trebouxia* clade A based on ITS rDNA, *rbc*L and cox2 locus dataset with posterior probabilities and bootstrap support values from *IQ-TREE* analysis presented near the branches. For each record from GenBank, accession no. with photobiont name are given (following Moya et al., 2017; Molins et al., 2018; Muggia et al., 2020). For newly sequenced samples, voucher no. and their mycobiont host name are given. Newly sequenced photobionts from Bolivia are marked in bold. Species or phylogenetic lineages to which Bolivian samples belong are marked in boxes with appropriate names. The first column of pie charts shows the percentage of specimens originating from open high Andean vegetation, upper montane cloud forest 2, upper montane cloud forest 1 and lower montane cloud forest in each *Trebouxia* lineage. The second column of pie charts shows the percentage of specimens in which defined groups of secondary metabolites were found in each *Trebouxia* lineage, absence of substances (A), presence of aliphatic (fatty) acids (B), anthraquinones (C), ergochromes (D), depsones (E), orcinol depsides (F), β-orcinol depsides (G), orcinol depsidones (H), β-orcinol depsidones (I), orcinol tridepsides (J), pulvinic acid derivatives (K), terpenoids (L), usnic acid derivatives (M), xanthones (N), and pigments (O). The next column of pie charts shows the percentage of specimens in each *Trebouxia* lineage with a given lichen growth form. In the last column of pie charts the percentage of given lichen family is shown. The color scheme is explained in the legend.

**FIGURE 2:**
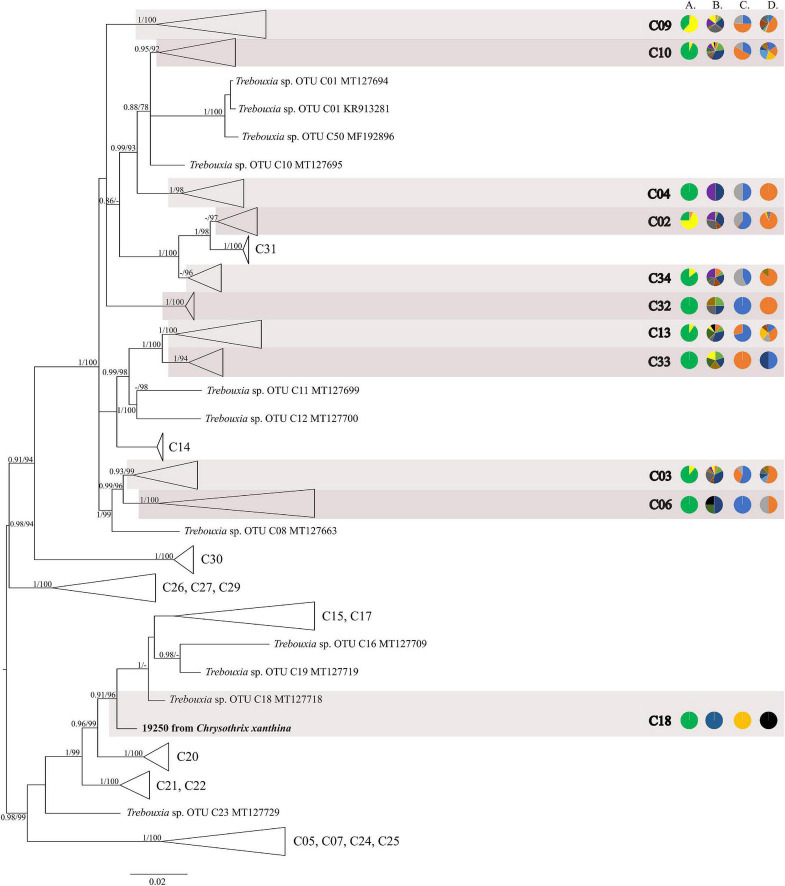
Collapsed majority-rule consensus tree from Bayesian analysis of *Trebouxia* clade C based on ITS rDNA and *rbc*L locus dataset with posterior probabilities and bootstrap support values from *IQ-TREE* analysis presented near the branches. For each record from GenBank, accession no. with photobiont name are given (following Škaloud et al., 2018; Muggia et al., 2020). For newly sequenced samples, voucher no. and their mycobiont host name are given. Newly sequenced photobionts from Bolivia are marked in bold. Species or phylogenetic lineages are marked in boxes with appropriate names. The first column of pie charts shows the percentage of specimens originating from open high Andean vegetation, upper montane cloud forest 2, upper montane cloud forest 1 and lower montane cloud forest in each *Trebouxia* lineage. The second column of pie charts shows the percentage of specimens in which defined groups of secondary metabolites were found in each *Trebouxia* lineage, absence of substances (A), presence of aliphatic (fatty) acids (B), anthraquinones (C), ergochromes (D), depsones (E), orcinol depsides (F), β-orcinol depsides (G), orcinol depsidones (H), β-orcinol depsidones (I), orcinol tridepsides (J), pulvinic acid derivatives (K), terpenoids (L), usnic acid derivatives (M), xanthones (N), and pigments (O). The next column of pie charts shows the percentage of specimens in each *Trebouxia* lineage with a given lichen growth form. In the last column of pie charts, the percentage of given lichen family is shown. The color scheme is explained in the legend on Figure 1.

**FIGURE 3:**
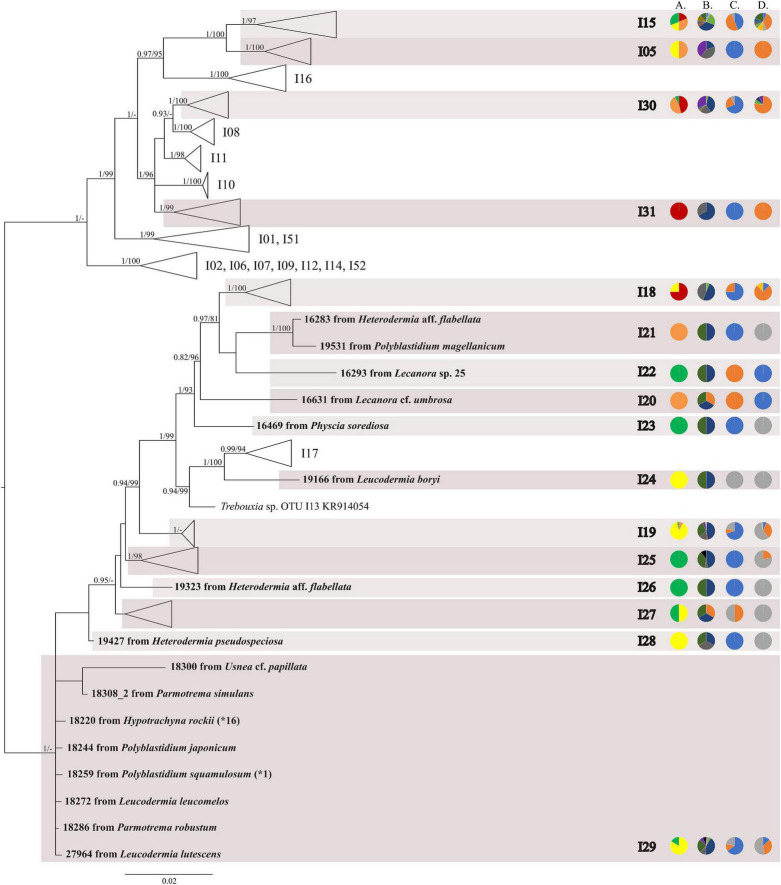
Collapsed majority-rule consensus tree from Bayesian analysis of *Trebouxia* clade I based on ITS rDNA and *rbc*L locus dataset with posterior probabilities and bootstrap support values from *IQ-TREE* analysis presented near the branches. For each record from GenBank, accession no. with photobiont names are given (following Molins et al., 2018; Muggia et al., 2020). For newly sequenced samples voucher no. and their mycobiont host names are given. Newly sequenced photobionts from Bolivia are marked in bold. Haplotypes that were represented by more than one sequence are marked with the star and number of occurrences. Species or phylogenetic lineages are marked in boxes with appropriate names. The first column of pie charts shows the percentage of specimens originating from open high Andean vegetation, upper montane cloud forest 2, upper montane cloud forest 1 and lower montane cloud forest in each *Trebouxia* lineage. The second column of pie charts shows the percentage of specimens in which defined groups of secondary metabolites were found in each *Trebouxia* lineage, absence of substances (A), presence of aliphatic (fatty) acids (B), anthraquinones (C), ergochromes (D), depsones (E), orcinol depsides (F), β-orcinol depsides (G), orcinol depsidones (H), β-orcinol depsidones (I), orcinol tridepsides (J), pulvinic acid derivatives (K), terpenoids (L), usnic acid derivatives (M), xanthones (N), and pigments (O). The next column of pie charts shows the percentage of specimens in each *Trebouxia* lineage with a given lichen growth form. In the last column of pie charts, the percentage of given lichen family is shown. The color scheme is explained in the legend on Figure 1.

**FIGURE 4:**
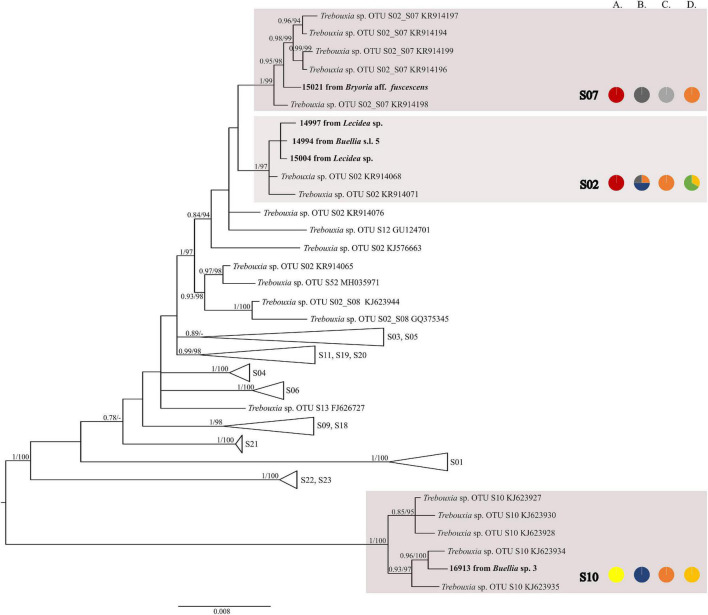
Collapsed majority-rule consensus tree from Bayesian analysis of *Trebouxia* clade S based on ITS rDNA, *rbc*L, and cox2 locus dataset with posterior probabilities and bootstrap support values from *IQ-TREE* analysis presented near the branches. For each record from GenBank, accession no. with photobiont name is given (following Molins et al., 2018; Muggia et al., 2020). For newly sequenced samples voucher no. and their mycobiont host names are given. Newly sequenced photobionts from Bolivia are marked in bold. Species or phylogenetic lineages are marked in boxes with appropriate names. The first column of pie charts shows the percentage of specimens originating from open high Andean vegetation, upper montane cloud forest 2, upper montane cloud forest 1 and lower montane cloud forest in each *Trebouxia* lineage. The second column of pie charts shows the percentage of specimens in which defined groups of secondary metabolites were found in each *Trebouxia* lineage, absence of substances (A), presence of aliphatic (fatty) acids (B), anthraquinones (C), ergochromes (D), depsones (E), orcinol depsides (F), β-orcinol depsides (G), orcinol depsidones (H), β-orcinol depsidones (I), orcinol tridepsides (J), pulvinic acid derivatives (K), terpenoids (L), usnic acid derivatives (M), xanthones (N), and pigments (O). The next column of pie charts shows the percentage of specimens in each *Trebouxia* lineage with a given lichen growth form. In the last column of pie charts, the percentage of given lichen family is shown. The color scheme is explained in the legend on Figure 1.

The first major clade A consisted of 58 *Trebouxia* lineages, of which two, A64 and A65 are newly described from Bolivia (Figure 1 and Supplementary Figure 1). Within the second major clade C, 34 lineages were found, of which three are new, i.e., C32, C33, and C34 (Figure 2 and Supplementary Figure 2). The third major clade I consists of 34 lineages, of which 11 were newly identified, i.e., lineages I21–I31 (Figure 3 and Supplementary Figure 3). One new lineage was recovered previously from South Africa (Fryday et al., 2020), but without phylogenetic affiliation; therefore, we named it here as I27. Finally, within clade S, only five sequences from Bolivia were placed, and they belong to *Trebouxia* spp. S02, S07, and S10 (Figure 4 and Supplementary Figure 4).

The most abundant lineages in our dataset were C09 (*N* = 120) and the recently distinguished I19 (*N* = 65) (Medeiros et al., 2021). Moreover, OTUs C02 (*N* = 28), C10 (*N* = 31), I29 (*N* = 24), C13 (*N* = 21), A05 (*N* = 16), and I30 (*N* = 13) were also frequently found in Bolivian lichens (Supplementary Table 1).

The majority of Bolivian samples were placed within clades C04, C09, and C10 (Figure 2). The samples placed in clade C09, apart from the Bolivian material, were also found in specimens from Brazil, Japan, Kenya, Peru, Russia, and the United States; those placed in clade C10 were identified in the material from Australia, Brazil, Japan, and Kenya (Supplementary Table 8). The newly discovered lineage C33 was found in samples originating from lower montane cloud forests at the altitude of c. 1,300 m a.s.l. and is closely related to lineages C11–C14 that were recovered from Kenya, and in the case of C12, also from Australia. Within clades C13 and C19 *Trebouxia* sequences from specimens collected in lower montane cloud forest were placed, together with two sequences from specimens found above 2,200 m a.s.l. Moreover, the closely related OTUs C03, C06, and C08 originated from specimens collected in Kenya and Uruguay (C03) and Japan (C06). In this mainly tropical group, 10 Bolivian specimens were placed, and they were collected below 1,900 m a.s.l. except in the case of *Ramalina celastri* (ID 19513a), which was recovered at 2,270 m a.s.l.

Within clade I, we recovered 11 new *Trebouxia* OTUs (Figure 3). Lineages I19–I24 are closely related to I18 and I17, which were previously recognized from Kenya, and to I13 from Ohio, United States. Moreover, the newly discovered OTUs I25–I29 are closely related to the lineages mentioned above. Altogether, 18 Bolivian samples were placed within *Trebouxia* sp. I05, which has a wide occurrence range (Canary Islands, United States, India, Italy, and Japan) and within I15, which was known only from Kenya (Supplementary Table 9). Newly recognized lineages I30 and I31 are closely related to OTUs I08, I10, and I11.

**FIGURE 5:**
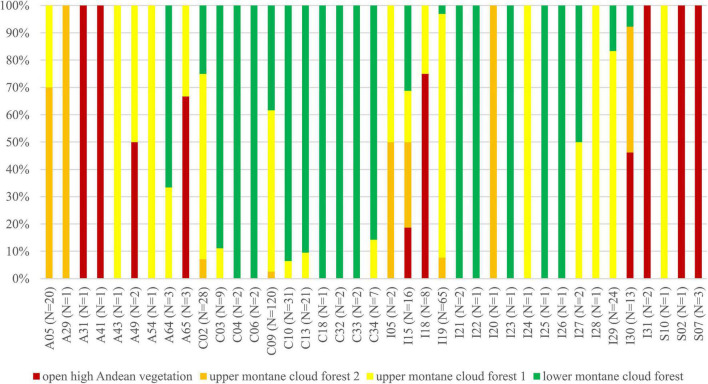
Bar graphs showing the percentage of specimens originating from open high Andean vegetation (red), upper montane cloud forest 2 (orange), upper montane cloud forest 1 (yellow) and lower montane cloud forest (green) in each *Trebouxia* lineage.

**FIGURE 6:**
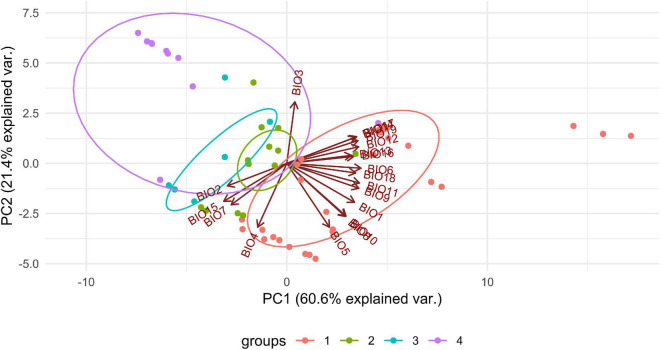
PCA result of *Trebouxia* distribution depending on climatic factors and selected habitat types in Bolivia. 1—lower montane cloud forest, 445–1,943 m a.s.l, 2—upper montane cloud forest 1, 2,130–2,879 m a.s.l, 3—upper montane cloud forest 2, 3,000–3,893 m a.s.l and 4—open high Andean vegetation, 4,020–4,850 m a.s.l.

We observed dissimilarities in photobiont composition within four habitat types: lower montane cloud forest (445–1,943 m a.s.l.), upper montane cloud forest 1 (2,130–2,879 m a.s.l.), upper montane cloud forest 2 (3,000–3,893 m a.s.l.), and open high Andean vegetation (4,020–4,850 m a.s.l.) (Figures 1–5 and Supplementary Table 4). In each habitat, we observed the lineages that were not detected in other regions. The PCA illustrated that the ranges of photobionts from different habitats may overlap (Figure 6), as some lineages were identified in more than one habitat type (Figure 5 and Supplementary Table 4). In lower montane cloud forest, the most abundant *Trebouxia* lineages were C09 (*N* = 46), C10 (*N* = 29), C13 (*N* = 19), I25 (*N* = 9), C03 (*N* = 8), and C02 (*N* = 7); in upper montane cloud forest 1, *Trebouxia* lineages C09 (*N* = 71) and I19 (*N* = 58) were dominating, while C02 (*N* = 19) were concurrent (Figure 5). In the second section of upper montane cloud forest, *Trebouxia* spp. A05 (*N* = 12), I30 (*N* = 6), I15 (*N* = 5), and I19 (*N* = 5) were the most abundant, while in open high Andean vegetation, the occurrences of *Trebouxia* spp. I18 (*N* = 6) and I30 (*N* = 6) were noted. Interestingly, the two most abundant lineages detected in Bolivian lichens, *Trebouxia* spp. C09 and I19, were not observed in open high Andean vegetation. Moreover, lineages belonging to clade C occurred mainly in lower montane cloud forest and upper montane cloud forest 1, with only few specimens (*N* = 5, 2%) occurring in upper montane cloud forest 2.

### Statistical Analysis

To reveal the local Bolivian community structure of the mycobionts toward the *Trebouxia* photobiont, we tested the influence of climate, altitude, geographical distance, habitat, substrate, as well as growth forms, propagation mode, and secondary metabolites composition in comparison with the effects of the symbiotic partner (mycobiont) at the genus, family, and secondary metabolites ranks. Distance-based redundancy analysis (Supplementary Table 5) revealed that growth forms were not statistically significant, so they were omitted. Variation partitioning showed that the mycobiont coded as the genus or family of lichen-forming fungi was the most important factor, while the climate was the second and altitude the third. The importance of the last two independent variables was lower than that of the mycobiont itself (Supplementary Figures 5a,b). At the family level, the correlation between mycobionts and photobionts was lower than at the mycobiont genus level, which may be caused by the low number of families analyzed in this study. Moreover, 17% of the variability was described by secondary metabolites (Supplementary Figure 5c). The PCA showed that the different lineages of *Trebouxia* photobionts within clades A, C, I, and S overlapped in their occupation of similar climates in Bolivia (Supplementary Figure 6). Clade S occupied a unique portion of hyperdimensional climate space. The PCA showed that the range of secondary metabolites overlapped in hyperdimensional space (Supplementary Figure 7).

Lichens exhibit various distribution patterns at macro levels, so we decided to proceed variation partitioning analysis at a global scale using all available sequences in GenBank with global positioning system (GPS) information available (*N* = 2,880, Supplementary Table 6). We excluded Antarctic data as they represent a different climatic zone; moreover, the available GPS data proved to be insufficient to retrieve correct bioclimatic data. The variation partitioning of all data at three different ranks revealed that the genus of mycobiont may be a dominating factor in photobiont selection, in connection with climatic conditions (Supplementary Figure 8); the correlation between particular photobiont lineage and the family of mycobiont is low (Supplementary Figure 8b). The composition of secondary metabolites explained 18% of variation as an independent factor, and 12% in co-correlation with climate and altitude (Supplementary Figure 8c). In the case of climatic conditions, PCA showed that photobionts from clade C occurred under different conditions than other *Trebouxia* lineages but still overlap with clades I and A (Supplementary Figure 9). In the case of secondary metabolites produced by lichen thalli, we found that all *Trebouxia* lineages overlap with each other (Supplementary Figure 10).

Due to the non-constant distribution of each major *Trebouxia* clade, i.e., clades C and I dominated in the tropics, while clades A and S in the temperate region, we conducted variation partitioning and PCA analysis separately for those four clades. In clade A (*N* = 1,080), we did not observe a strict separation of groups within climatic and secondary metabolites hyperdimensional space; however, not all groups overlap with each other (Supplementary Table 7 and Supplementary Figures 11, 12). Variation partitioning showed that the genetic diversity of *Trebouxia* clade A is correlated with mycobiont genus (41%) in co-correlation with climatic and altitudinal conditions (Supplementary Figure 13a). In the case of the mycobiont coded as family or secondary metabolites composition, the most important variable was the climate (Supplementary Figures 13b,c). Similarly, in clade C (*N* = 347), we did not observe the separation of any group within climatic and secondary metabolites hyperdimensional space (Supplementary Table 8 and Supplementary Figures 14, 15). Interestingly, in this clade, the correlation between the genetic diversity of photobionts and mycobionts at every rank was very low: mycobiont genus—10%, mycobiont family—8%, and secondary metabolites composition—10% in co-correlation with the remaining variables that were tested (Supplementary Figures 16a–c). In clade I (*N* = 381) (Supplementary Figure 17), photobiont groups I and V were shifted to BIO1, while groups III and IV to BIO4 and 7, and group II ranked in the middle. Groups I, II, and V have a similar range of occurrence (Kenya, Central Andes, Mediterranean region), as well as III and IV (tropical as well as temperate regions) (Supplementary Table 9). In the case of secondary metabolites hyperdimensional space, we observed a shift of the fifth group to β-orcinol depsides (G), but it was still overlapping with other groups (Supplementary Figure 18). Variation partitioning revealed that in *Trebouxia* clade I, the genetic diversity of photobionts was not only correlated with the mycobiont genus (29%) but also with climatic conditions, altitude, and spatial distribution (31%) (Supplementary Figure 19a). With mycobiont coded by family or secondary metabolites composition, the correlation was lower (9–10%) as independent factors, while 11–21% with the co-correlation of remaining variables (Supplementary Figures 19b,c). Clade S did not show the separation of any group within climatic and secondary metabolites hyperdimensional space (Supplementary Table 10 and Supplementary Figures 20, 21). Meanwhile, variation partitioning showed that selected variables are crucial as cumulative variables (Supplementary Figures 22a–c). The selected variables explained 62% of the variability with mycobiont coded as genus, 57% as family, and 57% as secondary metabolites composition (Supplementary Table 11).

**FIGURE 7:**
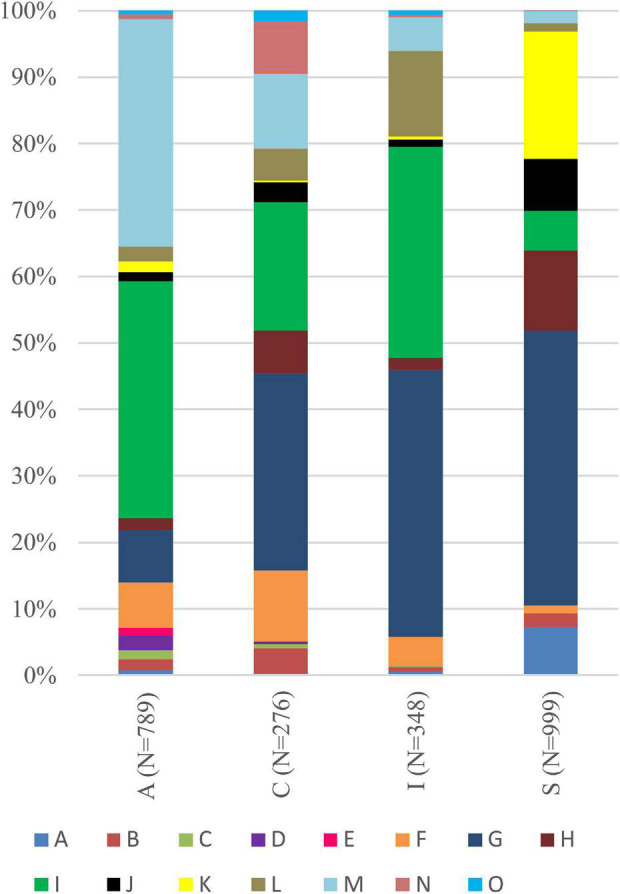
Bar graphs of the percentage occurrence of particular groups of lichen secondary metabolites found in lichen thalli with identified *Trebouxia* photobionts belonging to clades A, C, I, and S. Symbols A–O correspond to groups of secondary metabolites: no substances (A), presence of aliphatic (fatty) acids (B), anthraquinones (C), ergochromes (D), depsones (E), orcinol depsides (F), β-orcinol depsides (G), orcinol depsidones (H), β-orcinol depsidones (I), orcinol tridepsides (J), pulvinic acid derivatives (K), terpenoids (L), usnic acid derivatives (M), xanthones (N), and pigments (O).

**FIGURE 8:**
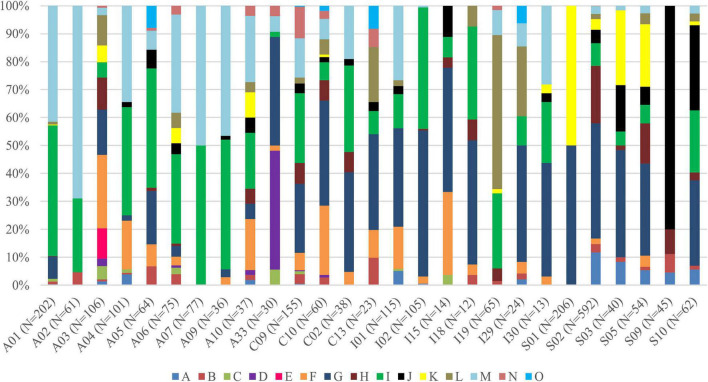
Bar graphs of selected *Trebouxia* photobiont OTUs showing the percentage occurrence of particular groups of lichen secondary metabolites found in lichen thalli. Symbols A–O correspond to groups of secondary metabolites: no substances (A), presence of aliphatic (fatty) acids (B), anthraquinones (C), ergochromes (D), depsones (E), orcinol depsides (F), β-orcinol depsides (G), orcinol depsidones (H), β-orcinol depsidones (I), orcinol tridepsides (J), pulvinic acid derivative (K), terpenoids (L), usnic acid derivatives (M), xanthones (N), and pigments (O).

Interestingly, for lichens with *Trebouxia* photobionts from clade A, we identified all selected secondary metabolites groups, while in the case of clade C, depsones were not present. In the case of clade S, four groups of secondary metabolites were not found, i.e., anthraquinones, ergochromes, depsones, and pigments, and were therefore excluded from further analysis (Figures 7, 8 and Supplementary Table 12). We proceeded the variation partitioning of photobiont diversity for only two variables as control, between the mycobiont coded as genus or family with the secondary metabolites composition. The results showed that secondary metabolites are important factors influencing the presence of photobiont in lichen thalli in the Bolivian dataset, in the general dataset, and separately for each clade (all variation partitioning analyses are summarized in Supplementary Table 11). The mycobionts that were related to *Trebouxia* photobionts from clades C and I in most cases produced secondary metabolites from β-orcinol depsides and β-orcinol depsidone groups, as well as orcinol depsides in several samples (Figure 7). In clade A, in the majority of specimens, β-orcinol depsidones and usnic acid derivatives were detected, while β-orcinol depsides occur in lichens associated with the following lineages: A01, A03, and A64 (Figure 8). In the case of lichen thalli that are associated with *Trebouxia* belonging to clade S, we found that they mostly produced β-orcinol depsides and pulvinic acid derivatives that were not common in the rest of the samples associated with *Trebouxia* from clades A, C, and I (Figures 7, 8).

**FIGURE 9:**
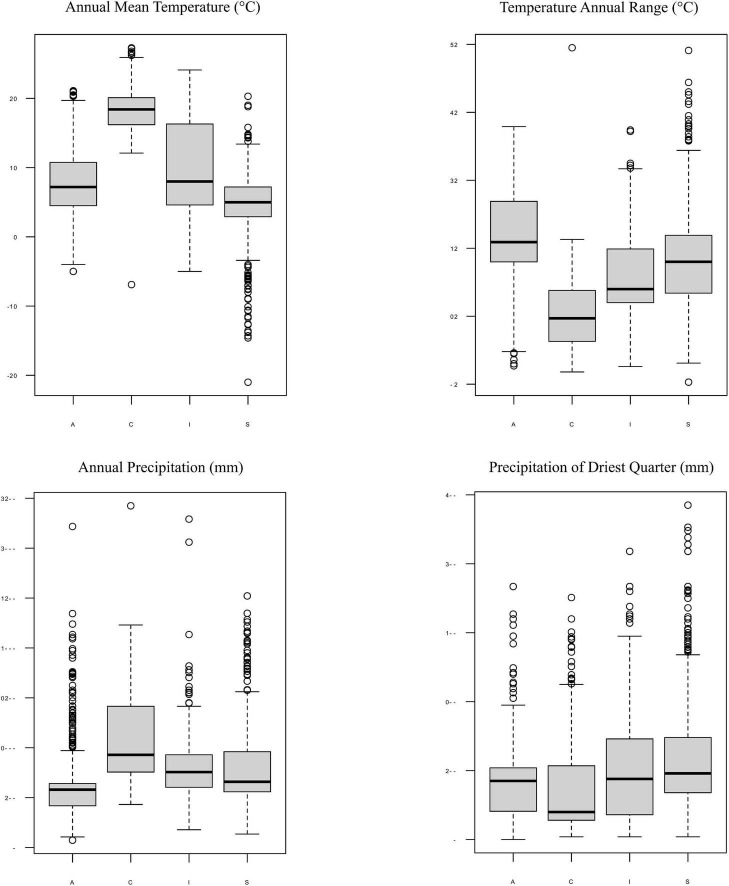
Box-plot diagrams representing differences in climatic preferences for four clades of *Trebouxia* photobionts—A, C, I, and S. Climatic data were obtained from the Global Climate Data—WorldClim.

Moreover, each *Trebouxia* lineage of clades A, C, I, and S may occur in different climatic condition ranges, but they overlap with each other in the worldwide range. The *Trebouxia* from clade A were noted in habitats with an annual mean temperature (BIO1) between –5°C to 21.1°C, clade C –6.9°C to 27.3°C, clade I –5°C to 24.1°C, and clade S –21°C to 20.3°C (Figure 9). Clade A shows the highest range of annual temperature range (BIO7) between 12.7 and 47.9°C, similarly to clade S – 10.3°C–59.1°C. Photobionts belonging to clade C may indicate the highest tolerance not only to heavy rains (BIO12 up to 3,424 mm) but also to drought (BIO17: 4–351 mm, median = 40 mm).

As the highest correlation, we identified that between the genus of mycobionts and photobiont genetic diversity, we performed further analysis for the different genera of lichen-forming fungi, i.e., *Lecanora* (Lecanoraceae), *Lecidea* (Lecideaceae), *Hypotrachyna*, *Parmotrema*, *Usnea*, *Xanthoparmelia* (Parmeliaceae), *Heterodermia*, *Polyblastidium* (Physciaceae), *Pertusaria* (Pertusariaceae), and *Lepra* (Variolariaceae).

**FIGURE 10:**
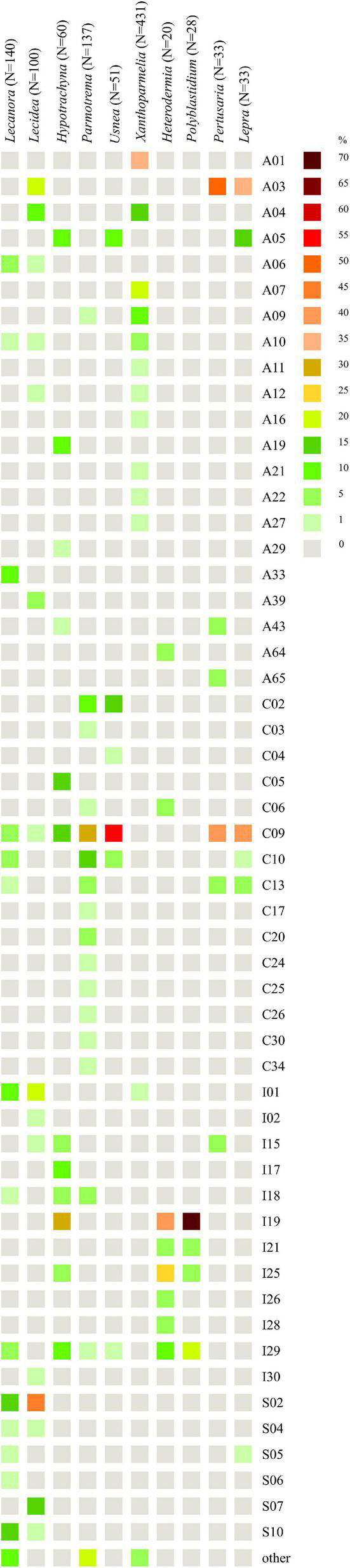
Heatmap of relative abundance of *Trebouxia* OTUs in genera *Lecanora* (Lecanoraceae), *Lecidea* (Lecideaceae), *Hypotrachyna*, *Parmotrema*, *Usnea*, *Xanthoparmelia* (Parmeliaceae), *Heterodermia*, *Polyblastidium* (Physciaceae), *Pertusaria* (Pertusariaceae), and *Lepra* (Variolariaceae).

We found that *Lecanora* spp. (*N* = 140) form a lichen symbiosis with 23 different OTUs of *Trebouxia* from four clades—A, C, I, and S (Figure 10), of which A03 (*N* = 30) was the most abundant, and S02 (*N* = 23) was the second and S10 (*N* = 19)—the third. Variation partitioning showed that 43% of the variability of photobiont diversity depend on lichen species and 32% with the co-correlation of climate and geographical distance (Supplementary Figure 23a). Analysis revealed that secondary metabolites’ composition explained 17% by itself and 19% with the correlation of climate and geographical distance, while climate as an independent factor described 16% of the variability (Supplementary Figure 23b). The climate dependence is visible in the PCA scheme (Supplementary Figure 24). Clades A and S were separated from clade C, which mostly occurs in tropics, while clade I overlaps with all those clades. In the case of the influence of secondary metabolites’ composition, clades C, I, and S showed different ranges in hyperdimensional space (Supplementary Figure 25).

For *Lecidea* (*N* = 100), we observed that this lichen genus is associated with *Trebouxia* spp. from 14 different OTUs belonging to all four clades (Figure 10). OTUs S02 (*N* = 45), I02 (*N* = 18), and S07 (*N* = 14) were the most abundant, while C09 was found in only one specimen from Bolivia. Variation partitioning indicated that 32% of the variation was explained by species identity alone, and 11% in correlation of climate and geographical distance (Supplementary Figure 26a). On the other hand, climate did not show an important influence (6%). Secondary metabolites composition explained 15% itself and 9% in summary with the climate (Supplementary Figure 26b). In climatic hyperdimensional space, we observed an overlap of all analyzed clades (Supplementary Figure 27). In secondary metabolites hyperdimensional space, we noticed that clade S occupied unique portions of hyperdimensional space (a shift to β-orcinol depsidones) (Supplementary Figure 28).

*Hypotrachyna* (*N* = 60), from the family Parmeliaceae, was studied in terms of photobionts that originated from Bolivia (this study), Kenya (Muggia et al., 2020), and Poland (one specimen, Singh et al., 2019). This work demonstrates that *Hypotrachyna* species form a symbiosis with *Trebouxia* spp. from clades A, C, and I, of which I19 (*N* = 16) was the most abundant in Bolivia, A05 (*N* = 10) and C09 (*N* = 8) were detected in Kenya and Bolivia, while the latter also in Japan and the United States (Figure 10). Variation partitioning demonstrated that 73% of the variability was explained by *Hypotrachyna* species identity (Supplementary Figure 29a), while secondary metabolites composition explained 24% without a co-correlation with remaining factors (Supplementary Figure 29b). These analyses indicated a very low correlation of *Hypotrachyna* photobionts with climatic conditions, as shown in the PCA scheme, where all three clades overlap in hyperdimensional space (Supplementary Figure 30). Interestingly, in the secondary metabolites hyperdimensional space, we observed a shift of clade C to orcinol depsidones (H), orcinol tridepsides (J), and xanthones (N) that were not detected in lichen specimens with *Trebouxia* photobionts from clades A and I (Supplementary Figure 31).

Another parmelioid genus, *Parmotrema* (*N* = 137), was studied in terms of photobionts using specimens from Bolivia (this study), Kenya (Muggia et al., 2020), and Japan (Ohmura et al., 2006). The representatives from this genus form a lichen symbiosis with 34 different *Trebouxia* OTUs from three clades—A, C, and I (Figure 10). Specimens with *Trebouxia* spp. A05, A09, and A19 originated from Kenya (three specimens) and Bolivia (two specimens) and from four different species. Moreover, photobionts from *Trebouxia* clade I were detected in *Parmotrema* for the first time. The most abundant *Trebouxia* OTUs were C09 (*N* = 39), C10 (*N* = 22), and C02 (*N* = 14). Variation partitioning analysis showed that 20% of the variability was explained by *Parmotrema* species (Supplementary Figure 32a). Climatic conditions had a low influence on photobiont diversity in this genus (9% in correlation with the remaining factors), whereas secondary metabolites composition showed insignificant influence (1%) (Supplementary Figure 32b). In the PCA scheme, we observed that the photobionts from clade C spanned a broad range—all three countries, while those from clade I, identified only in Bolivia, occupied unique portions of hyperdimensional space (Supplementary Figure 33). The specimens of *Parmotrema* that contained *Trebouxia* spp. belonging to the clade I originated from 1,790 to 4,780 m a.s.l. In terms of secondary metabolites, this factor appeared insignificant in this genus, for which the ranges of all analyzed clades overlapped in the PCA hyperdimensional space (Supplementary Figure 34).

*Usnea* (*N* = 51) is another representative of Parmeliaceae. Here, we focused on characterizing the diversity of *Trebouxia* OTUs associated with *Usnea* in Bolivia. We observed that this lichen-forming fungal genus can form lichen symbiosis with *Trebouxia* from clades A, C, and I (also, clade S was found in Antarctica (i.e., Domaschke et al., 2012), clade C in Japan—GenBank no. MK328539, while clade I in Korea—GenBank nos. MH258964-67, but GPS information was lacking). In Bolivia, we found eight different *Trebouxia* OTUs, of which C09 (*N* = 28) was the most abundant, followed by C02 (*N* = 8) (Figure 10). *Trebouxia* sp. A05 was found in lichen specimens collected above 3,000 m a.s.l. Variation partitioning revealed that 45% of the variability was explained by *Usnea* species identity (Supplementary Figure 35). Variation partitioning on secondary metabolites composition, climate, and geographical distance yielded ambiguous results; therefore, we decided to omit these analysis. In climatic and secondary metabolites hyperdimensional space, we did not observe any clusters (Supplementary Figures 36, 37).

*Xanthoparmelia* (*N* = 431) was the best sampled lichen genus in terms of photobiont diversity in Parmeliaceae (Leavitt et al., 2013, Leavitt et al., 2015). It was found that this genus formed lichen symbiosis with 24 different OTUs of *Trebouxia* from two clades—A and I (Figure 10). Clade A dominated, whereas photobionts belonging to clade I were found in only three specimens. The most abundant *Trebouxia* out was A01 (*N* = 153), followed by A07 (*N* = 77) and A04 (*N* = 56), although A06 (*N* = 41), A09 (*N* = 32), and A10 (*N* = 22) were also frequent. In two Bolivian specimens, *Xanthoparmelia microspora* (ID 16493) and *X. mexicana* (ID 16501) from localities above 4,000 m a.s.l., we identified *Trebouxia* spp. A41 and A49, respectively. *Trebouxia* sp. A41 was also found in *X. salkiboensis* from Kenya (MT127650) originating from the area above 4,000 m a.s.l. Interestingly, I01 (*N* = 3) and I04 (N = 1) were found only in the United States. Generally, *Xanthoparmelia* samples originated mostly from the United States, Canada, and Czech Republic; however, in Kenya clades A04, A10, and A27 were also identified (Muggia et al., 2020). Variation partitioning showed that the species of mycobionts (5%) and climate (9%) had a weak influence on photobiont diversity (Supplementary Figure 38a), whereas secondary metabolites composition explained 3% of the variability (Supplementary Figure 38b and Supplementary Table 11). We decided to visualize these data in a PCA scheme using only *Trebouxia* OTUs with abundant occurrence in *Xanthoparmelia* lichens. In climatic hyperdimensional space, we observed the shift of *Trebouxia* sp. A01 into BIO2 (Supplementary Figure 39). On the other hand, we did not observe any clustering of analyzed *Trebouxia* OTUs in secondary metabolites hyperdimensional space (Supplementary Figure 40).

*Heterodermia* (*N* = 20) (Physciaceae) was not well represented in terms of photobiont diversity. We obtained 20 sequences of *Trebouxia* spp. Associated with 11 *Heterodermia* species. This lichen-forming fungal genus formed a symbiosis with eight different *Trebouxia* OTUs (Figure 10) belonging to three clades, A, C, and I, of which clade I was the dominant one. The most abundant *Trebouxia* OTUs were I19 (*N* = 8) and I25 (*N* = 5). The dbRDA revealed that climatic factors were not significant for this dataset; therefore, we omitted the three factorial variation partitioning analysis. However, control analysis revealed that 34 and 15% of the variability were explained by *Heterodermia* species and secondary metabolites composition, respectively, as individual components (Supplementary Table 11).

*Polyblastidium* (*N* = 28) is another representative of Physciaceae. Similarly to *Heterodermia*, this group of lichens was not well studied in terms of associated photobionts. Therefore, we obtained 28 sequences of *Trebouxia* photobionts from four *Polyblastidium* species. We detected four *Trebouxia* OTUs from clade I only (Figure 10). *Trebouxia* sp. I19 was the most abundant (*N* = 20) and I29 was less abundant (*N* = 6), while I21 and I25 were detected in one specimen each. Variation partitioning showed that 15% as an individual factor and 33% in correlation with climatic conditions were explained by *Polyblastidium* species identity (Supplementary Figure 41a). Secondary metabolites composition accounted for only 4% of the variability (Supplementary Figure 41b). Control variation partitioning between mycobiont species, secondary metabolites’ composition and photobiont diversity showed that 34 and 6% of the variability were explained, respectively, by these factors (Supplementary Table 11). In the case of PCA, we did not observe clustering in climatic and chemical hyperdimensional space (Supplementary Figures 42, 43).

Concerning photobiont diversity in *Pertusaria*, only *P. coccodes* and *P. leioplaca* from Poland were previously included in the study by Singh et al. (2019). Here, we analyzed 17 specimens of *Pertusaria* from Bolivia. We found that this genus of lichen-forming fungi formed a symbiosis with six different *Trebouxia* OTUs from three clades, A, C, and I (*N* = 33) (Figure 10). The most common were A03 (*N* = 16) and C09 (*N* = 13). Variation partitioning analysis revealed that 36% of the variability was explained by *Pertusaria* species identity (Supplementary Figure 44a) and 41% was co-correlated within all selected factors. In the case of the influence of secondary metabolites composition on the photobiont diversity, in juxtaposition with climatic and geographical factors, this analysis showed that all selected components were co-correlated by 24% of the variability in summary (Supplementary Figure 44b). On the other hand, control variation partitioning exposed that 58 and 21% of the variability were explained by *Pertusaria* species and secondary metabolites’ composition, respectively (Supplementary Table 11). In climatic hyperdimensional space, we observed a significant separation between *Trebouxia* photobionts from clades A and C in terms of the conditions in which *Pertusaria* specimens and their photobionts occurred (Supplementary Figure 45). Clade A was shifted to BIO4 and BIO7, which, in the case of this particular situation, signified higher temperature annual range and higher temperature seasonality. Interestingly, in *Pertusaria* sp. (ID 18577) collected at 2,545 m a.s.l. and *P. tesselaria* (ID 16892a) collected at 2,879 m a.s.l., for which the above values were in the upper range of conditions for Bolivia, we also found the representatives of *Trebouxia* clade A. In secondary metabolites hyperdimensional space (Supplementary Figure 46), we noticed a shift of *Trebouxia* clade A into presence of anthraquinones I and β-orcinol depsidone (I), while clade C into xanthones (N), aliphatic acids (B), and orcinol depsides (F).

Concerning photobionts studied in *Lepra* (Variolariaceae), Singh et al. (2019) found *Trebouxia* sp. A03 as a photosymbiotic component in *Lepra amara* from Poland, whereas Peksa et al. (2022) recovered *Trebouxia* sp. S05 in *L. corallina* from the Czechia. Here, we obtained 30 *Trebouxia* sequences from *Lepra* specimens from localities from 465 to 3,780 m a.s.l. We summarized all information regarding the photobionts of this genus (*N* = 48), and we recovered that *Trebouxia* sp. C09 (*N* = 20) and A03 (*N* = 17) were the most abundant lineages (Figure 10). However, we did not find *Trebouxia* sp. A03 in Bolivia. Interestingly, we found *Trebouxia* sp. A05 in seven specimens of *Lepra* from high altitudes (2,760–3,780 m a.s.l.). Variation partitioning revealed a co-correlation between all selected factors in both series of analysis performed using the species of *Lepra* as an explanatory variable and secondary metabolites composition (Supplementary Figures 47a,b). On the other hand, control analysis uncovered 21% of the variability explained by the species of mycobiont, while 56% by secondary metabolites (Supplementary Table 11). In climatic hyperdimensional space, we observed a shift of *Trebouxia* clades A and S to higher temperature annual range and higher temperature seasonality (Supplementary Figure 48), whereas clade C was shifted into higher annual mean temperature and annual precipitation. In secondary metabolites hyperdimensional space, we noticed an overlap of ranges with a shift of *Trebouxia* sp. clade A into the presence of depsones (E) in lichen thalli, but those were related to a homogenous sampling of *L. amara* from Poland by Singh et al. (2019; Supplementary Figure 49).

## Discussion

We recovered 16 new lineages in three recognized clades, A, C, and I, in Bolivian Andean vegetation and additionally, lineage I27 that was previously found in South Africa but remained undescribed. The screening of unexplored areas, such as our multi-species and multi-genera approach, enables the verification of biodiversity and through sequencing methods, also the hidden biodiversity of *Trebouxia* photobionts, while single-species and single-genus sampling probably leads to the slip of this biodiversity.

It was already shown that photobiont diversity can be shaped by altitudinal gradient (Vargas-Castillo and Beck, 2012; Kosecka et al., 2020, 2021), the reproductive and dispersal strategies of the mycobiont (Cao et al., 2015; Steinová et al., 2019), geography (Muggia et al., 2014a; Werth and Sork, 2014; Leavitt et al., 2015), substrate (Bačkor et al., 2010b; Leavitt et al., 2013; Muggia et al., 2014a), and macroclimate (Leavitt et al., 2015). Here, we found a pattern of the occurrence of particular *Trebouxia* OTUs depending on altitudinal gradient. Interestingly, the representatives of *Trebouxia* clades A and S appeared as lichen photobionts in specimens from above 1,943 m a.s.l., while photobionts from clade C were noted mostly from areas located below 2,879 m a.s.l. Furthermore, we noticed that the climatic ranges of particular *Trebouxia* clades overlap (Figure 6), but to understand the relations that occurred in this particular Andean environment, we had to consider climatic dependencies.

Nelsen et al. (2021) suggested that understanding the macroecological preferences of symbionts may reveal the processes underlying niche differentiation and insight into their potential responses to environmental disturbances. Furthermore, they attempted to reveal the evolutionary lability of the macroecological preferences of *Trebouxia* algae in order to understand the evolution of ecological tolerances in the evolutionary history of these algae (Nelsen et al., 2021). Our estimate is consistent with the proposal of Nelsen et al. (2021) that the clade I occupies warmer, as well as cooler and drier, habitats, i.e., Eastern Europe. Moreover, *Trebouxia* spp., belonging to clade C, occupy hot and humid climate in partially or exclusively forested habitats (Nelsen et al., 2021). This may indicate the preferences of the representatives of the entire clade C, which may be the reason why we did not observe the presence of this lineage above the tree line in the Andes (above 3,600 m a.s.l.). According to the estimate of Nelsen et al. (2021), clade A also occupied only or partially forested habitats, and it subsequently expanded in the Cenozoic Era to occupy regimes characterized by cooler and drier habitats—loosely coinciding with or after clade S invasion to similar climatic regimes. However, this estimate may not be complete due to the uneven and unrepresentative sampling in public database in the data that were used in the work of Nelsen et al. (2021). The latest data indicate that this clade has wider ecological preferences and in fact, it is dominant in a temperate climate (i.e., in Lecanoraceae, Parmeliaceae, and Physciaceae) but has also been recorded not only in the Mediterranean climate (Singh et al., 2019; Molins et al., 2020; Moya et al., 2021), in the tropics (Kenya; Muggia et al., 2020), and in Parmeliaceae but also in Umbilicariaceae, originating from the United States (Rolshausen et al., 2020). Furthermore, *Trebouxia* spp. belonging to clade A were relatively abundant in Bolivia. We found this algal lineage in 33 specimens from eight families: Acarosporaceae, Caliciaceae, Lecanoraceae, Ochrolechiaceae, Parmeliaceae, Pertusariaceae, Physciaceae, and Variolariaceae (Supplementary Figure 1). Those specimens were collected from low mountain (from 1,500 m a.s.l) as well as high mountain areas (up to 4,380 m a.s.l). In the case of clade S, it consists of taxa that occupy cooler and drier climates, continued development in cool, temperate habitats and coinciding with the diversification of *Trebouxia*-associated lineages of lichen-forming fungi, such as the cetrarioid core (Nelsen et al., 2021). Those data correspond to our finding of two OTUs from clade S at high elevation (4,396–4,650 m a.s.l) where they were associated with *Bryoria* (Parmeliaceae), *Buellia* (Caliciaceae), and *Lecidea* (Lecideaceae), which are frequent in such climatic conditions (Lindgren et al., 2014; Singh et al., 2019; Ruprecht et al., 2020). Interestingly, the exception in clade S is *Trebouxia* sp. S10, which is known to occur also in the Mediterranean (Sadowska-Deś et al., 2013) and the Neotropical climate (Costa Rica—Nelsen and Gargas, 2009; Bolivia—Garrido-Benavent et al., 2020).

Such exceptions as in clades A and S prompted us to perform variation partitioning analysis separately for each clade. The determination of the distribution patterns of lichenized algae was the topic of numerous publications (i.e., Kroken and Taylor, 2000; Řídká et al., 2014; Kosecka et al., 2020). Here, we showed that in *Trebouxia* clades A, I, and S, mycobiont identity has the greatest influence on the distribution of the photobiont lineages. Moreover, clade I showed a slightly higher correlation with the mycobiont genus (29%), which may indicate the intermediate level of relationship of these photobionts in lichen symbiosis, while clades A and S revealed moderate dependence between photobiont diversity and their hosts. Thus, we need more extensive and proportional sampling for diverse habitats to be able to describe properly the specificity level of those algal lineages. However, clade C, without a doubt, demonstrates generalist relationships in lichen symbiosis, as the influence of mycobiont genera in variation partitioning was low (Supplementary Figure 16a). In this case, climate seems to be the most important factor shaping the diversity of those *Trebouxia* spp. Furthermore, each of *Trebouxia* clusters shows its own specific preferences, but by overlapping successive niches, they enable the continuity of the lichen symbiosis (Figure 9 and Supplementary Figure 9). The subclades inside the individual clades do not separate in climatic space, although they have different habitat preferences (Supplementary Figures 11, 14, 17, 20). However, this does not exclude the detection of the possible presence of the specialist pattern that has been described for *Nostoc*-associated lichen fungi (Otálora et al., 2010).

The family Lecanoraceae includes over 25 genera and about 791 described species (Lücking et al., 2016). *Lecanora* is the core genus of the family. Previous research about photobiont diversity in this genus revealed that *Lecanora pulicaris* can share a photobiont pool with *Pseudevernia furfuracea*, *Hypogymnia physodes*, and *H. tubulosa* from the same tree trunk (Mark et al., 2020). Mark et al. (2020) hypothesized that the mycobiont-driven selection for symbiont is determined by photobiont ecological specialization in combination with symbiont interaction efficiency, as *L. pulicaris* showed the highest genetic and distributional differences between the photobionts. This is consistent with our results on the photobiont diversity in *Lecanora* on a global scale. The correlation between mycobiont species and photobiont diversity explained the majority of the variability (43%) (Supplementary Figure 23a). The photobiont diversity of several other representatives of Lecanoraceae have been studied, e.g., *Bryonora*, *Lecanora, Myriolecis* (Medeiros et al., 2021), *Protoparmeliopsis* (Guzow-Krzemińska, 2006; Muggia et al., 2013; Leavitt et al., 2015, 2016; Medeiros et al., 2021), *Rhizoplaca* (Leavitt et al., 2015, 2016), and *Tephromela* (Muggia et al., 2014a). Medeiros et al. (2021) presented that the distribution of *Trebouxia* species from *Lecanora* and *Myriolecis* was structured by elevation, substrate, and mycobiont identity. *Rhizoplaca* and *Protoparmeliopsis* showed low specificity, while in *Tephromela*, a strong selectivity of the mycobionts for the photobionts was observed in six monophyletic *Tephromela* clades (Guzow-Krzemińska, 2006; Muggia et al., 2014a,b; Leavitt et al., 2015, 2016).

A similar situation was observed in *Lecidea*. Mycobiont species explained 32% of the variability in variation partitioning analysis, but this correlation is combined with the local availability of algal lineages. Here, we did not apply extensive sampling for this lichen genus; however, depending on the environment, *Lecidea* in Bolivia adopts available *Trebouxia* photobionts. Furthermore, Ruprecht et al. (2020) revealed that the majority of *Lecidea*, as well as *Porpidia* and *Poeltidea* sampled from southern South America, harbor cosmopolitan *Trebouxia* lineages, i.e., S02 and S07. They also found highly diverse and locally differentiated and/or endemic lineages of *Trebouxia* and *Asterochloris* (Ruprecht et al., 2020).

Leavitt et al. (2015) suggested that the fungal host genus determines the composition of the algal partner more than ecology at the ecoregions scale. Furthermore, they hypothesized that the evolutionary history of lichen-forming fungi is important and a potentially dominant factor in building interactions in the lichen symbioses of Parmeliaceae. Here, we summarized and complemented the data regarding the diversity of photobionts in four parmelioid genera: *Hypotrachyna*, *Parmotrema*, *Usnea*, and *Xanthoparmelia.*

Interestingly, the highest proportion of previously unrecognized species diversity of *Trebouxia* associated with *Hypotrachyna* and *Parmotrema* (Parmeliaceae) in East Africa was identified within the clade C (Muggia et al., 2020). In the case of *Hypotrachyna*, we found that the genus was poorly studied in terms of photobiont diversity. We tested 42 specimens from Bolivia and found that the photobionts belonged to clades A, C, and I. The newly recognized lineage of *Trebouxia* I19 (*N* = 16) was the most abundant in the Bolivian samples of *Hypotrachyna*; however, it was also associated with the representatives of *Lecanora, Leucodermia, Polyblastidium, Remototrachyna*, and *Usnea.* Moreover, the diversity of photobionts in *Hypotrachyna* was highly dependent on mycobiont species (73%), while climatic factors did not show much impact (Supplementary Figure 29a). This parmelioid genus deserves more attention, as it may represent a specific pattern of mycobiont–photobiont interaction. In our study, we obtained 63 sequences of *Trebouxia* photobionts from *Parmotrema* spp., in which we found two new *Trebouxia* OTUs, i.e., I29 and I31. Furthermore, variation partitioning revealed that only 20% of the variability was explained by *Parmotrema* species identity. This genus of lichen-forming fungi may also be consistent with habitat-adaptive hypothesis (Rodriguez et al., 2008), assuming the possibility of choosing locally available strains of green algae optimal for the development of thalli.

In the case of *Usnea*, *Trebouxia jamesii* (A03) is associated with widespread *Usnea aurantiaco-atra* in Fildes Peninsula (Cao et al., 2017). Here, we obtained 51 sequences of *Trebouxia* photobionts from *Usnea*. We suppose that *Usnea* exhibits a generalist pattern of interactions in lichen symbiosis, as we showed non-specific relationships with many *Trebouxia* lineages in Bolivia.

Leavitt et al. (2015) showed that *Xanthoparmelia* species are associated with an extensive range of *Trebouxia* species and/or lineages at a local scale, but, at a broader range, they associate with a similar pool of *Trebouxia* photobionts, different from the photobiont pools of accompanying *Rhizoplaca* spp. We made a similar observation in this study, i.e., in two *Xanthoparmelia* specimens, we identified distinct *Trebouxia* OTUs (A41, A49) in comparison to the remaining Bolivian specimens.

*Heterodermia* and *Polyblastidium* have not been studied well in respect to their photobionts, in contrast to the other representatives of Physciaceae, i.e., *Physcia* (Beck et al., 1998; Dahlkild et al., 2001; Helms et al., 2001; Mark et al., 2020) and *Physconia* (Wornik and Grube, 2010). In our study, we found that *Heterodermia* species form a lichen symbiosis with *Trebouxia* photobionts from clades A, C, and I, and those from clade I are the most common. However, *H. flabellata* (ID 18916) and *Heterodermia* sp. (ID 18921_2) are exceptions as they are associated with *Trebouxia* spp. A64 and C06, respectively. Further studies are needed to explore the cause of this condition. *Polyblastidium* spp. were found to interact exclusively with four different *Trebouxia* lineages within clade I. Additionally, in *Leucodermia* from Bolivia (*N* = 17, Supplementary Table 1), we found a photobiont belonging to *Trebouxia* clades I and C. Further analysis is needed to determine whether the relationships within Physciaceae are dependent on certain factors. While we do not observe distinct biogeographic patterns of lichen-forming fungi of Physciaceae family and their algal partners, photobiont diversity associated with *Polyblastidium* was co-correlated with climatic factors in Bolivia (Supplementary Figure 41a).

In the case of species belonging to *Pertusaria* (Pertusariaceae) and *Lepra* (Variolariaceae), we supplemented the homogenous sampling of *Pertusaria coccodes*, *P. leioplaca*, and *L. amara* from Poland. In a previous study for these genera, *Trebouxia* sp. A03 was found to be the most abundant photobiont (Singh et al., 2019) and S05 was found in one specimen of *L. corallina* (Peksa et al., 2022). Based on available data, we estimated that the diversity of *Trebouxia* photobionts in *Pertusaria* and *Lepra* show little dependence at the species level of those lichen-forming fungi (Supplementary Figures 44a, 47a). Singh et al. (2019) assumed that the genus *Pertusaria* is more selective toward the photobionts, depending on the climatic conditions. We revealed that in Andean vegetation, *Pertusaria* as well as *Lepra* form lichen symbiosis with *Trebouxia* spp. from clades C and A at higher altitudes, where the temperature range is lower. In the case of *Pertusaria* and *Lepra*, more extensive sampling is required to determine whether selectivity is climatically dependent. Understanding the climatic tolerances of mycobiont–photobiont relationships is interesting, especially in sterile lichens, as it was previously shown that algal swapping can extend the range of fungus by associating with locally adapted algae (Nelsen and Gargas, 2009).

The vegetation growing in the Andes undoubtedly experiences severe abiotic stresses such as desiccation, extreme temperatures, and high light levels (Pullaiah, 2019). Lichens have the ability to tolerate extreme stress and have been termed “extremophiles,” organisms that can thrive under conditions that do not allow other, less specialized organisms to survive (Beckett et al., 2021). Additionally, Beckett et al. (2021) suggested that acclimatization can be distinguished from adaptation, which applies to a genetically determined level of resistance arising through selection. Huneck and Yoshimura (1996) noted that most lichen secondary metabolites absorb UV-B radiation very efficiently. Furthermore, lichens show significant plasticity in response to high light stress, as demonstrated by their ability to show seasonal changes in photosynthesis and the ability of lichens to show “sun” or “shadow” forms (Gauslaa and Goward, 2020). Gauslaa and Goward (2020) also showed that in heavily melanized, sun-exposed thalli of *Lobaria pulmonaria*, the photobiont could still be shade adapted. Furthermore, another strategy to protect thalli is that many secondary compounds occur as crystals outside fungal hyphae (Gauslaa and Goward, 2020). Therefore, in this context, it is worth investigating whether the production of secondary metabolites may be the crucial factor allowing the selection of certain *Trebouxia* photobiont lineages and shaping their selectivity and specificity toward some mycobionts. Lokajová et al. (2014) hypothesized that lichen photobiont evolved protective mechanisms against the phytotoxicity of secondary metabolites through co-evolution. Furthermore, the higher resistance to usnic acid in *Trebouxia* cultures may be an adaptation resulting from the long co-evolution of these algae with fungi that produce secondary metabolites (Bačkor et al., 2010a). Here we summarized the occurrence of *Trebouxia* clades in lichens with respect to the secondary metabolites composition of the lichen thallus to test whether the presence of certain secondary metabolites groups is correlated with *Trebouxia* photobiont selection. In this study, we selected 15 groups of lichens’ secondary metabolites. Despite the fact that we observed the correlation of chemical factors with photobiont diversity in the group of Bolivian lichens (17%) and in all available data (24%) (Supplementary Table 11), it is difficult to conclude here about the resistance of photobionts to the metabolites produced by lichen-forming fungi. A separate variation partitioning on the available data of *Trebouxia* clades showed a weak influence of secondary metabolites composition. However, control variation partitioning revealed that *Trebouxia* clade A (18%) and C (6%) showed low dependence on secondary metabolites composition, while dependence was moderate in clade I (29%) and high in clade S (51%). Based on the secondary metabolites composition in the available dataset for clade A, we can hypothesize that those *Trebouxia* photobionts exhibit moderate resistance to depsides and usnic acid derivatives and preference to lichens that can produce β-orcinol depsidones. *Trebouxia* clade C shows potentially strong resistance to depsides, and moderate resistance to usnic acid derivatives (especially in the case of C09). *Trebouxia* clade I, on the other hand, may show resistance to β-orcinol depsides and usnic acids derivatives. Furthermore, in lichens that harbor *Trebouxia* clade I we noticed the frequent production of β-orcinol depsidones. The resistance to pulvinic acid derivatives may be characteristic for *Trebouxia* clade S. This *Trebouxia* lineage may also show resistance to β-orcinol depsides.

More data are needed to determine whether the occurrence of protective substances is a result of the high specificity of lichens in relation to a given *Trebouxia* lineage. We still lack comprehensive data not only on sunlight exposure but also on other factors, such as the age of lichen, location of the secondary metabolities in the thallus, elevation, temperature fluctuations, and seasonality, and its influence on the production of protective substances (Kosanić and Ranković, 2015). A project with a selected group of lichens at various sites, both sunny and shaded, considering anthropogenic factors, would be needed to study the dynamics of lichens in adaptation to and of protection of specific photobionts. Also, determining at what point secondary metabolites become toxic to the photobionts, even if they protect algal layer from intense light levels (Waring, 2008), would be of interest.

## Conclusion

The investigation of photobiont diversity in light of the many factors that determine the stability of the lichen symbiosis has become complex, as the diversity of the photosymbiotic partners also shapes the symbiosis. The knowledge of the evolutionary preferences of lichen-forming fungi, i.e., the selectivity and specificity, will identify noteworthy groups of lichens that can be used as model organisms to study all factors affecting symbiosis (including lichen metabolites), in specialists, intermediates, and generalists in lichen symbioses. Despite many years of research, we still find understudied areas in terms of lichens and their photobiont biodiversity, which can be essential for studying adaptation strategies in the lichen symbiosis. We believe that the multi-species and multi-genera approach adopted here should help to solve this problem on a global scale, especially in biodiversity hotspots such as Andean vegetation.

## Data Availability Statement

The datasets presented in this study can be found in online repositories. The names of the repository/repositories and accession number(s) can be found in the article/Supplementary Material.

## Author Contributions

MKo, BG-K, and MKu designed the study and wrote the manuscript. MKu, AF, and PR-F conducted the fieldwork and collected the specimens. MKu determined most of the lichen specimens and lichen secondary substances with contribution of AF and PR-F. MKo, AJ, and PR-F performed the laboratory work with contributions from BG-K. MKo and ŁP constructed the database. MKo analyzed the data with contributions from BG-K and MKu. All authors edited the manuscript.

## Conflict of Interest

The authors declare that the research was conducted in the absence of any commercial or financial relationships that could be construed as a potential conflict of interest.

## Publisher’s Note

All claims expressed in this article are solely those of the authors and do not necessarily represent those of their affiliated organizations, or those of the publisher, the editors and the reviewers. Any product that may be evaluated in this article, or claim that may be made by its manufacturer, is not guaranteed or endorsed by the publisher.
