# Supplementary material for: Phylogeny and Ecology of Trebouxia Photobionts From Bolivian Lichens
Source: Front Microbiol. 2022 Mar 28;13:779784. doi: 10.3389/fmicb.2022.779784 (PMC8996191; doi:10.3389/fmicb.2022.779784)
Supplement: Supplementary file 3 [file Data_Sheet_3.pdf]

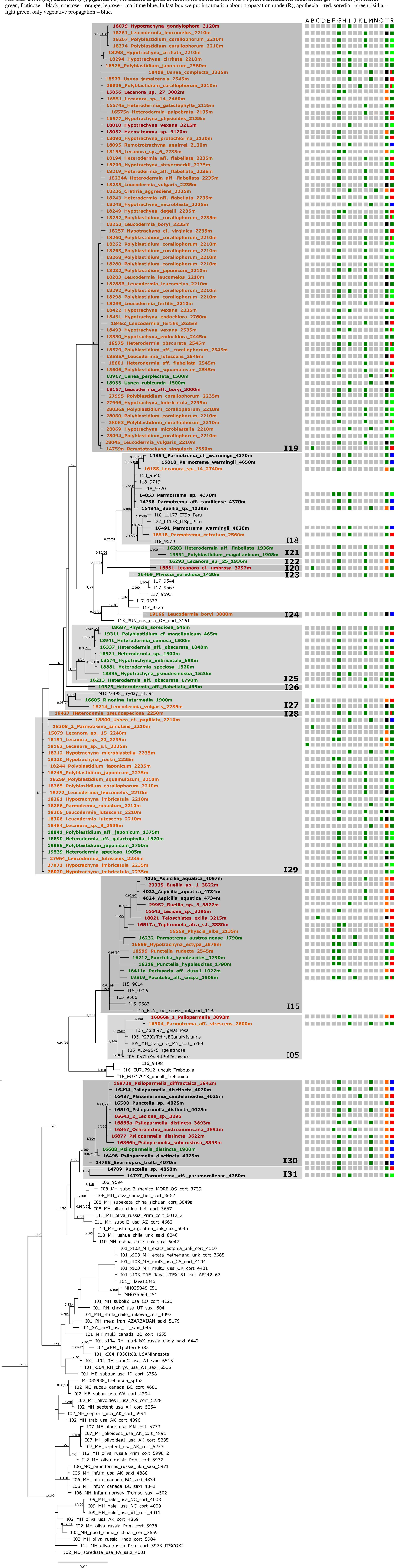

Supplementary Figure 3. Majority-rule consensus tree from Bayesian analysis of *Trebouxia* sp. clade I based on ITS rDNA and rbcL locus data set with posterior probabilities and bootstrap support values from *IQ-TREE* analysis presented near the branches. For each record from GenBank, accession no. with photobiont name were given (followed Molins et al., 2018; Muggia et al., 2020). For newly sequenced samples voucher no. and their mycobiont host name and altitude were given. Based on altitude of the newly sequenced samples we marked them in proper colours depending on habitat type; green bold - lower montane cloud forest, 445 – 1943 m a.s.l, orange bold – upper montane cloud forest 1, 2130 – 2879 m a.s.l, red bold - upper montane cloud forest 2, 3000 – 3893 m a.s.l, and black bold - open high Andean vegetation, 4020 – 4850 m a.s.l. Regarding the secondary metabolites detected in lichen thallus by TLC, we added information which group of secondary metabolites were detected in particular sample. Absence of substances are marked in green in first box (A). Presence of aliphatic (fatty) acids (B), anthraquinones (C), ergochromes (D), depsones (E), orcinol depsides (F), β-orcinol depsides (G), orcinol depsidones (H), β-orcinol depsidones (I), orcinol tridepsides (J), pulvinic acid derivatives (K), terpenoids (L), usnic acid derivatives (M), xanthones (N), pigments (O) are marked in green in subsequent boxes. In next box we placed information about lichen growth form (T); foliose – green, fruticose – black, crustose – orange, leprose – maritime blue. In last box we put information about propagation mode (R); apothecia – red, soredia – green, isidia – light green, only vegetative propagation – blue.
