# Supplementary material for: Phylogeny and Ecology of Trebouxia Photobionts From Bolivian Lichens
Source: Front Microbiol. 2022 Mar 28;13:779784. doi: 10.3389/fmicb.2022.779784 (PMC8996191; doi:10.3389/fmicb.2022.779784)
Supplement: Supplementary file 5 [file Data_Sheet_5.pdf]

Supplementary Material

1.1 Supplementary Figures

Supplementary Figure 1. Majority-rule consensus tree from Bayesian analysis of *Trebouxia* sp. clade A based on ITS rDNA, *rbc*L and cox2 locus data set with posterior probabilities and bootstrap support values from *IQ-TREE* analysis presented near the branches. For each record from GenBank, accession no. with photobiont name were given (followed Moya et al., 2017; Molins et al., 2018; Muggia et al., 2020). For newly sequenced samples voucher no. and their mycobiont host name and altitude were given. Based on altitude of the newly sequenced samples we marked them in proper colours depending on habitat type; green bold - lower montane cloud forest, 445 – 1943 m a.s.l, orange bold – upper montane cloud forest 1, 2130 – 2879 m a.s.l, red bold - upper montane cloud forest 2, 3000 – 3893 m a.s.l, and black bold - open high Andean vegetation, 4020 – 4850 m a.s.l. Regarding the secondary metabolites detected in lichen thallus by TLC, we added information which group of secondary metabolites were detected in particular sample. Absence of substances are marked in green in first box (A). Presence of aliphatic (fatty) acids (B), anthraquinones (C), ergochromes (D), depsones (E), orcinol depsides (F), β-orcinol depsides (G), orcinol depsidones (H), β-orcinol depsidones (I), orcinol tridepsides (J), pulvinic acid derivatives (K), terpenoids (L), usnic acid derivatives (M), xanthones (N), pigments (O) are marked in green in subsequent boxes. In next box we placed information about lichen growth form (T); foliose – green, fruticose – black, crustose – orange, leprose – maritime blue. In last box we put information about propagation mode (R); apothecia – red, soredia – green, isidia – light green, only vegetative propagation – blue.

Supplementary Figure 2. Majority-rule consensus tree from Bayesian analysis of *Trebouxia* sp. clade C based on ITS rDNA and *rbc*L locus data set with posterior probabilities and bootstrap support values from *IQ-TREE* analysis presented near the branches. For each record from GenBank, accession no. with photobiont name were given (followed Škaloud et al., 2018; Muggia et al., 2020). For newly sequenced samples voucher no. and their mycobiont host name and altitude were given. Based on altitude of the newly sequenced samples we marked them in proper colours depending on habitat type; green bold - lower montane cloud forest, 445 – 1943 m a.s.l, orange bold – upper montane cloud forest 1, 2130 – 2879 m a.s.l, red bold - upper montane cloud forest 2, 3000 – 3893 m a.s.l, and black bold - open high Andean vegetation, 4020 – 4850 m a.s.l. Regarding the secondary metabolites detected in lichen thallus by TLC, we added information which group of secondary metabolites were detected in particular sample. Absence of substances are marked in green in first box (A). Presence of aliphatic (fatty) acids (B), anthraquinones (C), ergochromes (D), depsones (E), orcinol depsides (F), β-orcinol depsides (G), orcinol depsidones (H), β-orcinol depsidones (I), orcinol tridepsides (J), pulvinic acid derivatives (K), terpenoids (L), usnic acid derivatives (M), xanthones (N), pigments (O) are marked in green in subsequent boxes. In next box we placed information about lichen growth form (T); foliose – green, fruticose – black, crustose – orange, leprose – maritime blue. In last box we put information about propagation mode (R); apothecia – red, soredia – green, isidia – light green, only vegetative propagation – blue.

Supplementary Figure 3. Majority-rule consensus tree from Bayesian analysis of *Trebouxia* sp. clade I based on ITS rDNA and *rbc*L locus data set with posterior probabilities and bootstrap support values from *IQ-TREE* analysis presented near the branches. For each record from GenBank, accession no. with photobiont name were given (followed Molins et al., 2018; Muggia et al., 2020). For newly sequenced samples voucher no. and their mycobiont host name and altitude were given. Based on altitude of the newly sequenced samples we marked them in proper colours depending on habitat type; green bold - lower montane cloud forest, 445 – 1943 m a.s.l, orange bold – upper montane cloud forest 1, 2130 – 2879 m a.s.l, red bold - upper montane cloud forest 2, 3000 – 3893 m a.s.l, and black bold - open high Andean vegetation, 4020 – 4850 m a.s.l. Regarding the secondary metabolites detected in lichen thallus by TLC, we added information which group of secondary metabolites were detected in particular sample. Absence of substances are marked in green in first box (A). Presence of aliphatic (fatty) acids (B), anthraquinones (C), ergochromes (D), depsones (E), orcinol depsides (F), β-orcinol depsides (G), orcinol depsidones (H), β-orcinol depsidones (I), orcinol tridepsides (J), pulvinic acid derivatives (K), terpenoids (L), usnic acid derivatives (M), xanthones (N), pigments (O) are marked in green in subsequent boxes. In next box we placed information about lichen growth form (T); foliose – green, fruticose – black, crustose – orange, leprose – maritime blue. In last box we put information about propagation mode (R); apothecia – red, soredia – green, isidia – light green, only vegetative propagation – blue.

Supplementary Figure 4. Majority-rule consensus tree from Bayesian analysis of *Trebouxia* sp. clade S based on ITS rDNA, *rbc*L and cox2 locus data set with posterior probabilities and bootstrap support values from *IQ-TREE* analysis presented near the branches. For each record from GenBank, accession no. with photobiont name were given (followed Molins et al., 2018; Muggia et al., 2020). For newly sequenced samples voucher no. and their mycobiont host name and altitude were given. Based on altitude of the newly sequenced samples we marked them in proper colours depending on habitat type; green bold - lower montane cloud forest, 445 – 1943 m a.s.l, orange bold – upper montane cloud forest 1, 2130 – 2879 m a.s.l, red bold - upper montane cloud forest 2, 3000 – 3893 m a.s.l, and black bold - open high Andean vegetation, 4020 – 4850 m a.s.l. Regarding the secondary metabolites detected in lichen thallus by TLC, we added information which group of secondary metabolites were detected in particular sample. Absence of substances are marked in green in first box (A). Presence of aliphatic (fatty) acids (B), anthraquinones (C), ergochromes (D), depsones (E), orcinol depsides (F), β-orcinol depsides (G), orcinol depsidones (H), β-orcinol depsidones (I), orcinol tridepsides (J), pulvinic acid derivatives (K), terpenoids (L), usnic acid derivatives (M), xanthones (N), pigments (O) are marked in green in subsequent boxes. In next box we placed information about lichen growth form (T); foliose – green, fruticose – black, crustose – orange, leprose – maritime blue. In last box we put information about propagation mode (R); apothecia – red, soredia – green, isidia – light green, only vegetative propagation – blue.

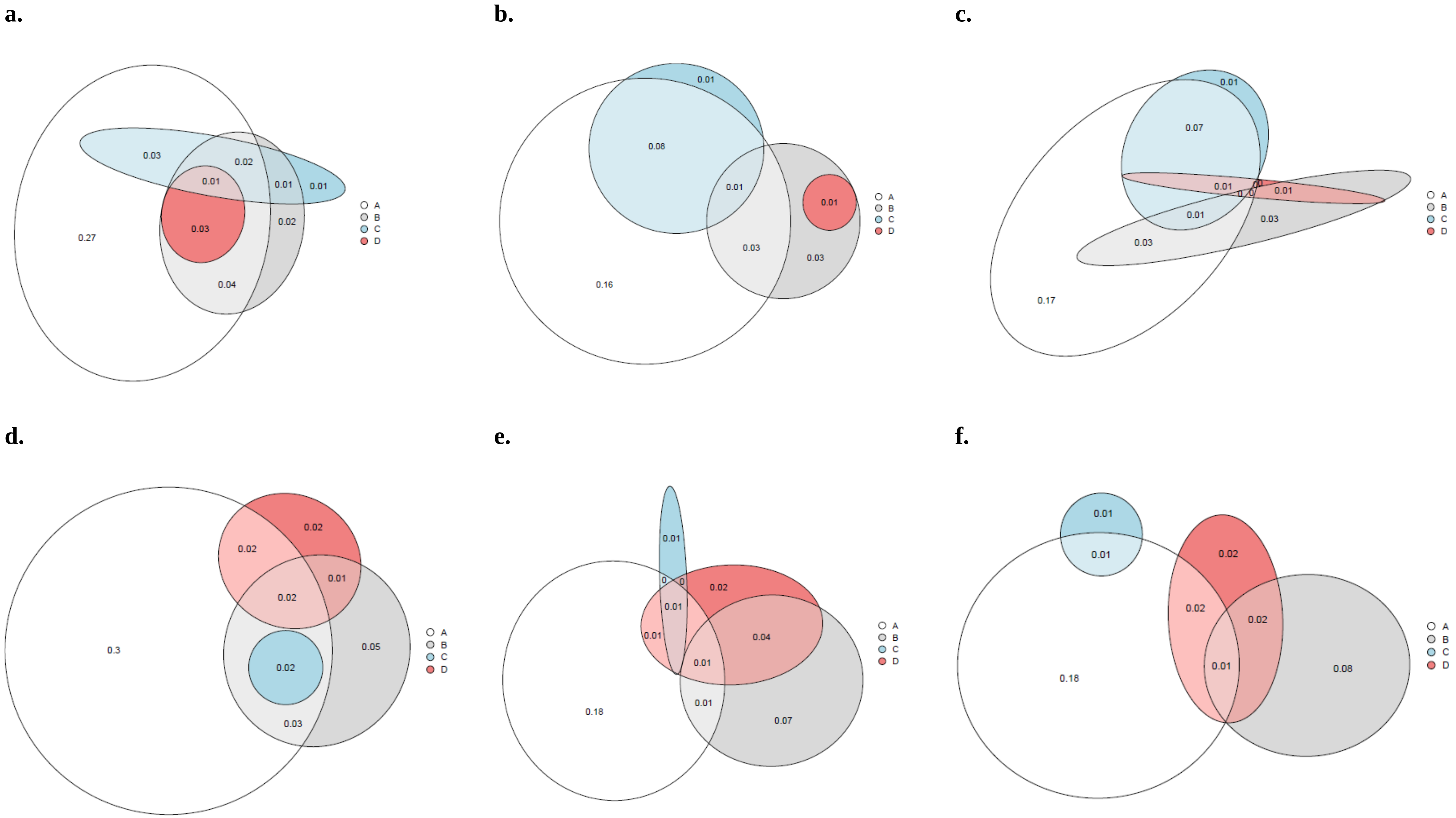

Supplementary Figure 5. Venn diagrams showing the variation partitioning of the genetic variation of Bolivian *Trebouxia* photobiont explained by each group of explanatory variables (A – mycobiont, B – climatic variable, C- altitude, D - geographical distances for Supplementary Figures a – c; A –mycobiont, B – habitat, C – propagation mode, D – substrate for Supplementary Figures d – f); a, d – genus of mycobiont, b, e – family of mycobiont, c, f – secondary metabolites composition.

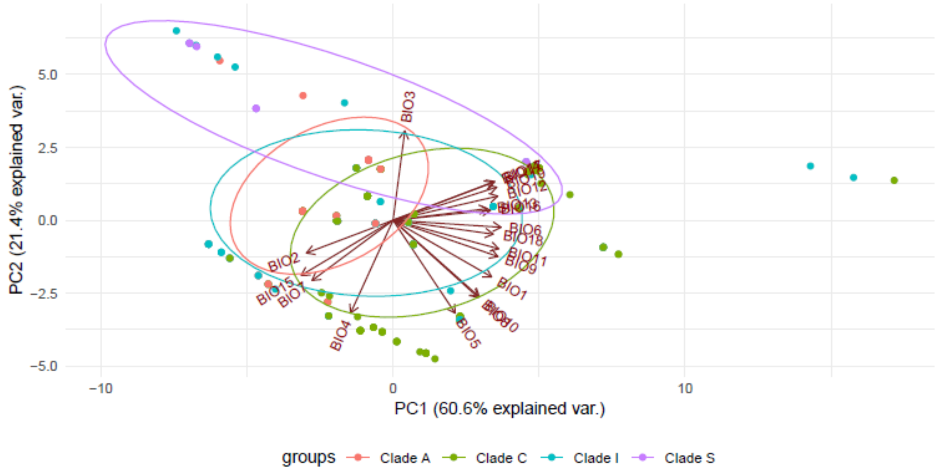

Supplementary Figure 6. PCA result of Bolivian *Trebouxia* distribution depending on climatic factors and *Trebouxia* major clades (N=419).

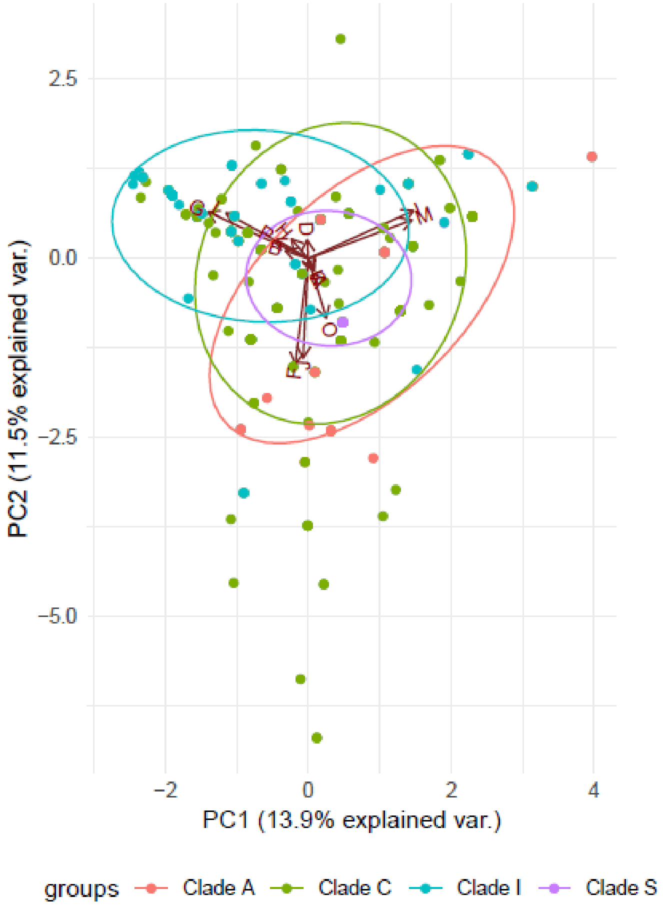

Supplementary Figure 7. PCA result of Bolivian *Trebouxia* distribution depending on secondary metabolites factors and *Trebouxia* major clades (N=419). No substances (A). Presence of aliphatic (fatty) acids (B), anthraquinones (C), ergochromes (D), depsones (E), orcinol depsides (F), β-orcinol depsides (G), orcinol depsidones (H), β-orcinol depsidones (I), orcinol tridepsides (J), pulvinic acid derivatives (K), terpenoids (L), usnic acid derivatives (M), xanthones (N), pigments (O).

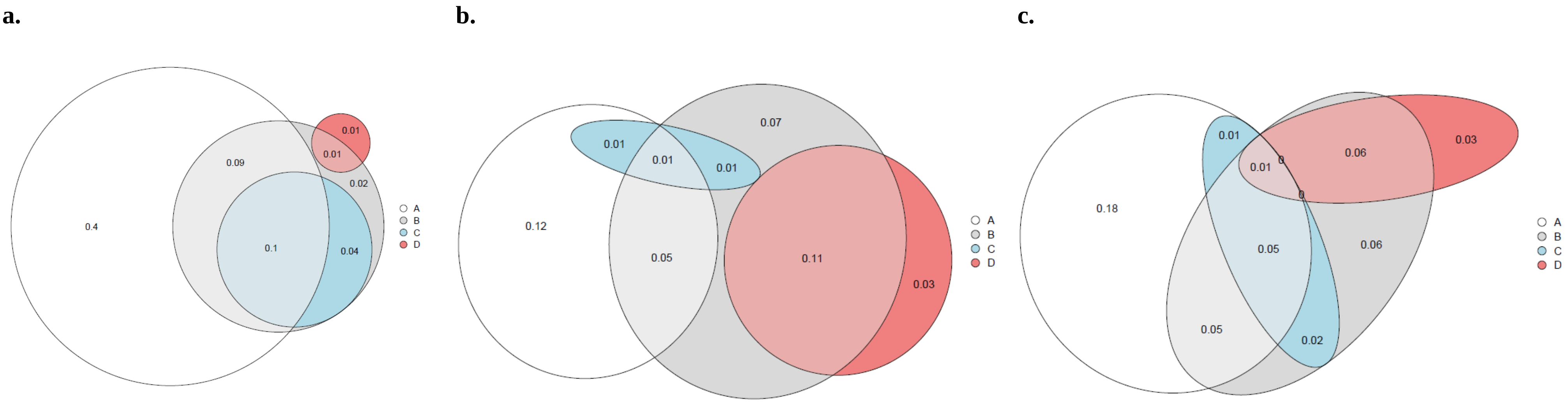

Supplementary Figure 8. Venn diagrams showing the variation partitioning of the genetic variation of *Trebouxia* photobiont explained by each group of explanatory variables based on dataset of all available in GenBank (N=2880) (A – mycobiont, B – climatic variable, C- altitude, D - geographical distances) a – genus of mycobiont, b – family of mycobiont, c – secondary metabolites composition.

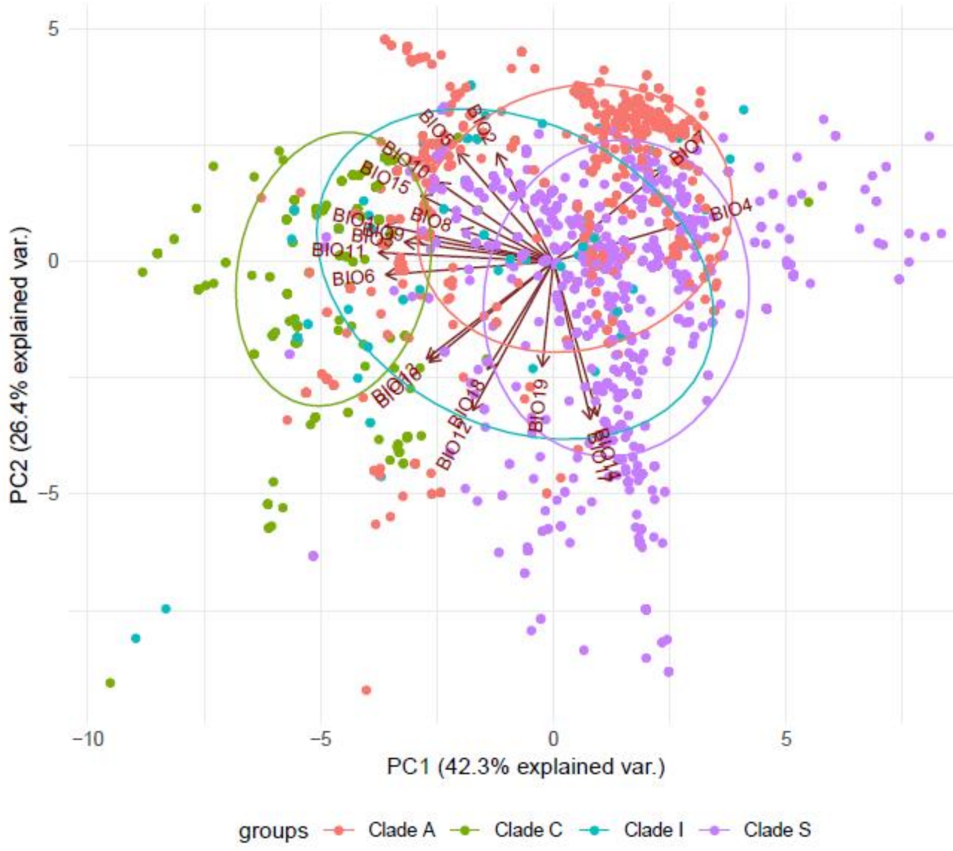

Supplementary Figure 9. PCA result of *Trebouxia* distribution depending on climatic factors and *Trebouxia* major clades, based on dataset of all available in GenBank (N=2880).

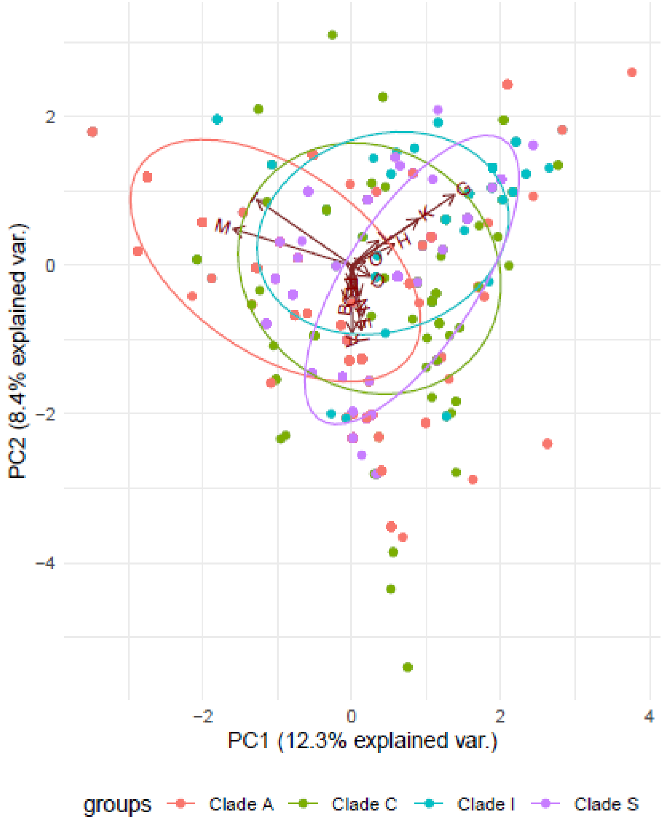

Supplementary Figure 10. PCA result of *Trebouxia* distribution depending on secondary metabolites factors and *Trebouxia* major clades, based on dataset of all available in GenBank (N=2880). No substances (A). Presence of aliphatic (fatty) acids (B), anthraquinones (C), ergochromes (D), depsones (E), orcinol depside (F), β-orcinol depsides (G), orcinol depsidone (H), β-orcinol depsidone (I), orcinol tridepside (J), pulvinic acid derivative (K), terpenoids (L), usnic acid derivatives(M), xanthones (N), pigments (O).

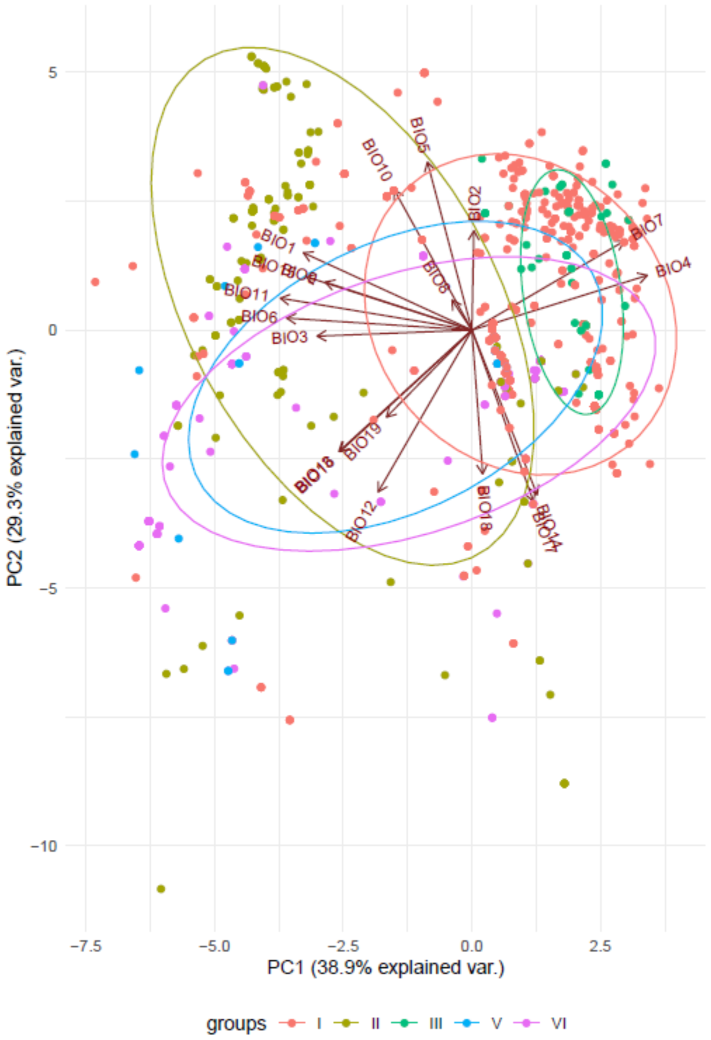

Supplementary Figure 11. PCA result of *Trebouxia* distribution depending on climatic factors and *Trebouxia* major clades, based on dataset of Clade A (N=1080).

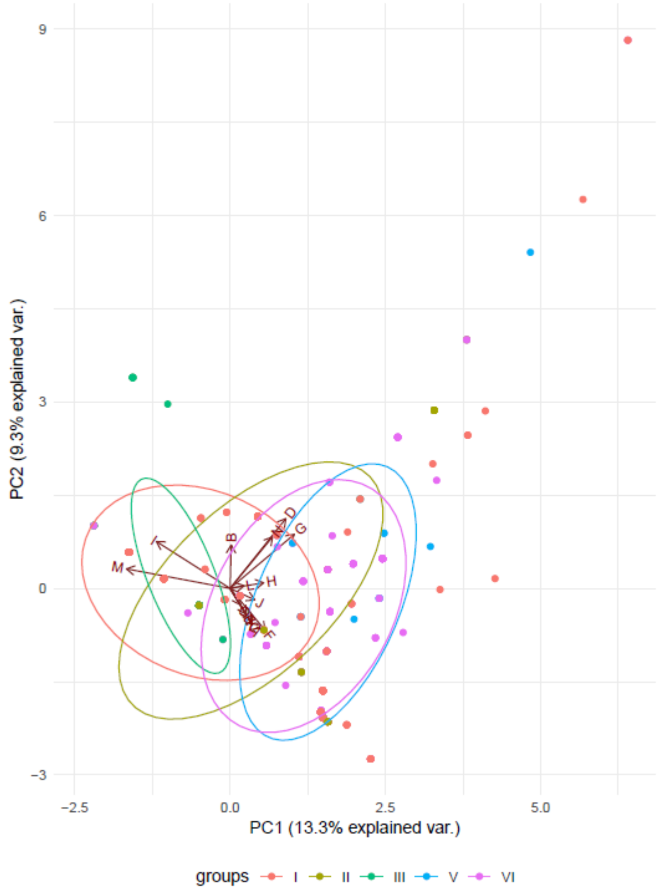

Supplementary Figure 12. PCA result of *Trebouxia* distribution depending on secondary metabolites factors and *Trebouxia* major clades, based on dataset of Clade A (N=1080). No substances (A). Presence of aliphatic (fatty) acids (B), anthraquinones (C), ergochromes (D), depsones (E), orcinol depsides (F), β-orcinol depsides (G), orcinol depsidones (H), β-orcinol depsidones (I), orcinol tridepsides (J), pulvinic acid derivatives (K), terpenoids (L), usnic acid derivatives (M), xanthones (N), pigments (O).

a. **b.** **c.**

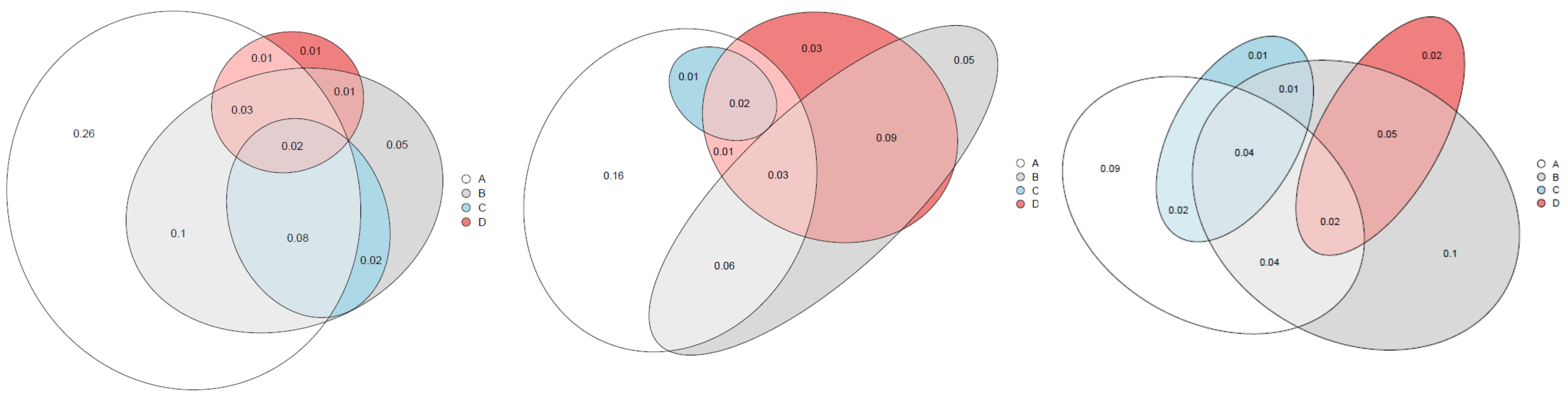

Supplementary Figure 13. Venn diagrams showing the variation partitioning of the genetic variation of *Trebouxia* photobiont explained by each group of explanatory variables based on dataset of Clade A (N=1080) (A – mycobiont, B – climatic variable, C- altitude, D - geographical distances) a – genus of mycobiont, b – family of mycobiont, c – secondary metabolites composition.

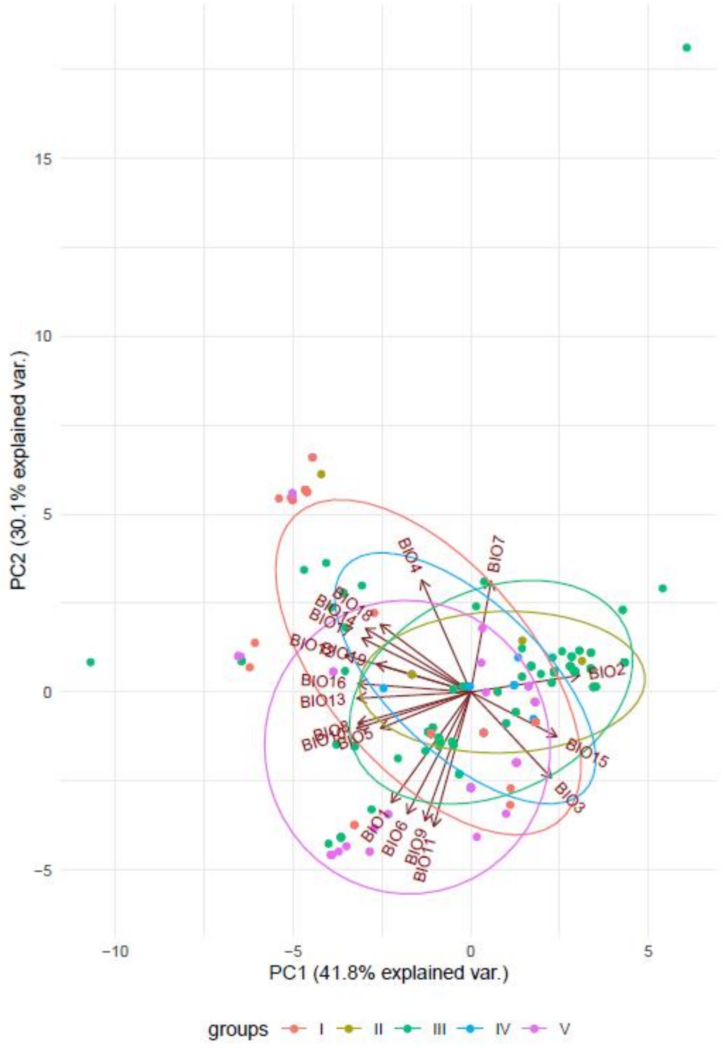

Supplementary Figure 14. PCA result of *Trebouxia* distribution depending on climatic factors and *Trebouxia* major clades, based on dataset of Clade C (N=347).

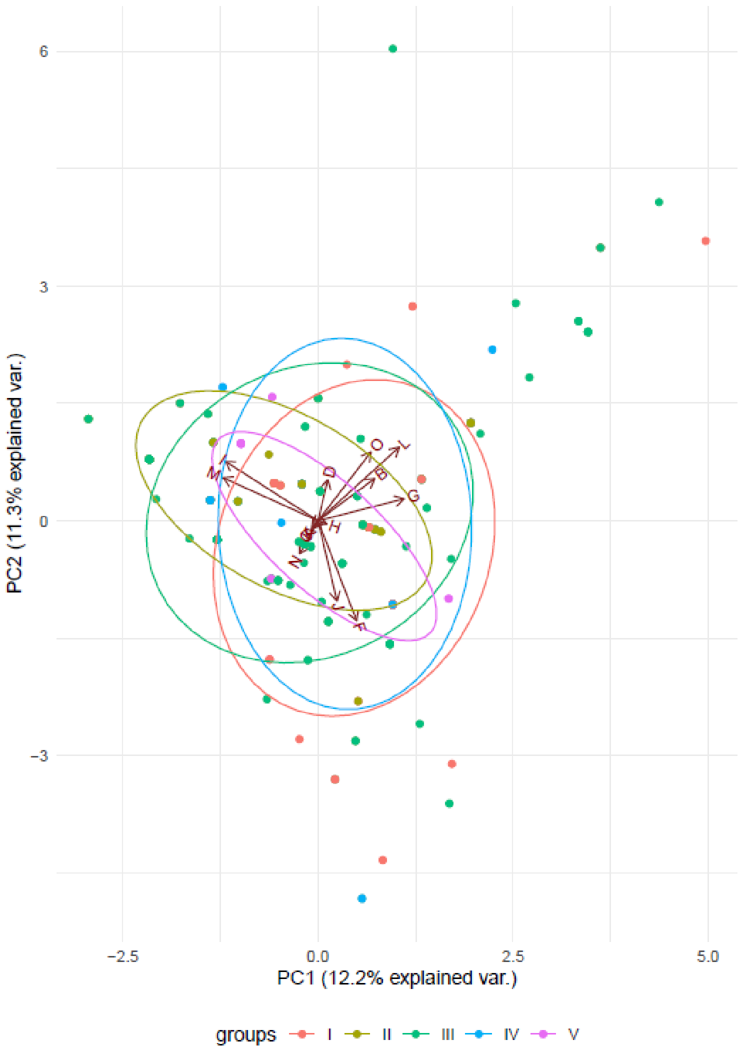

Supplementary Figure 15. PCA result of *Trebouxia* distribution depending on secondary metabolites factors and *Trebouxia* major clades, based on dataset of Clade C (N=347). No substances (A). Presence of aliphatic (fatty) acids (B), anthraquinones (C), ergochromes (D), depsones (E), orcinol depsides (F), β-orcinol depsides (G), orcinol depsidones (H), β-orcinol depsidones (I), orcinol tridepsides (J), pulvinic acid derivatives (K), terpenoids (L), usnic acid derivatives (M), xanthones (N), pigments (O).

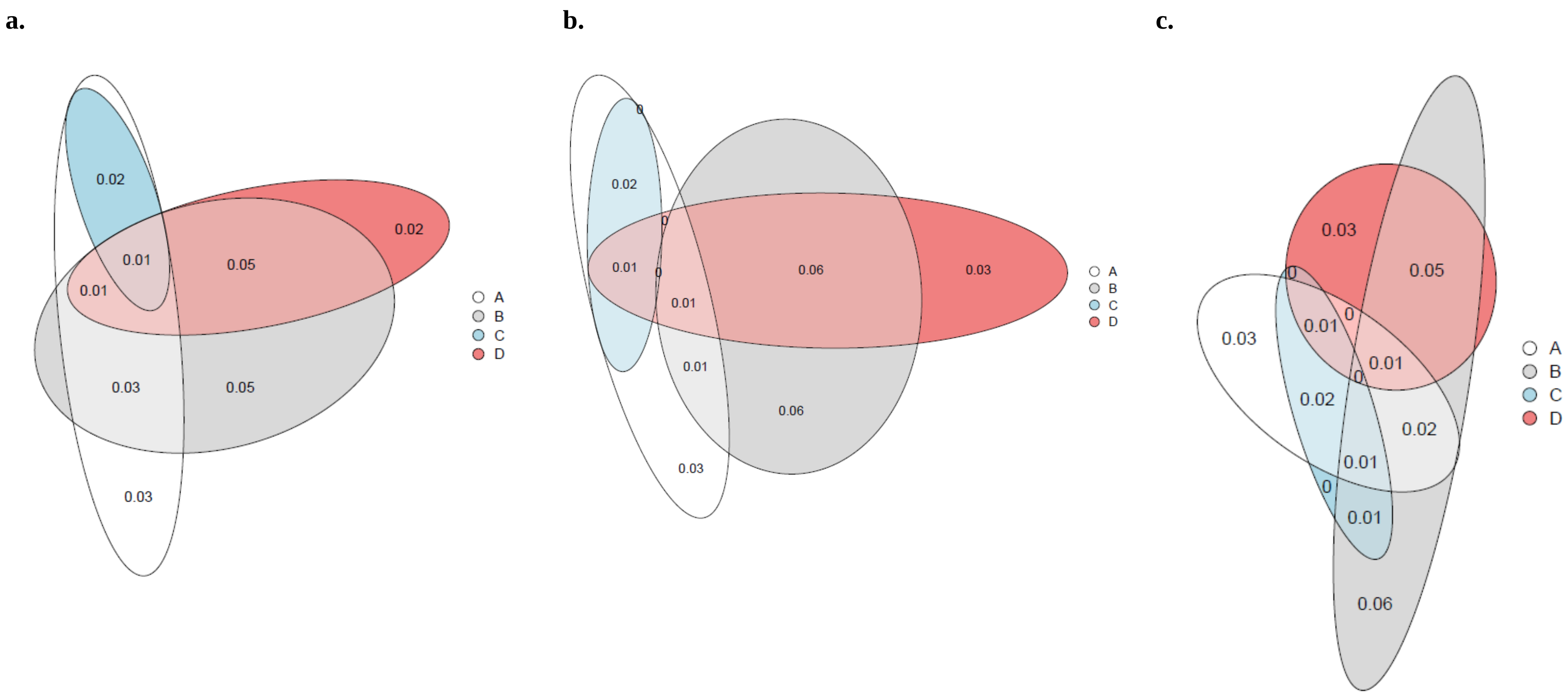

Supplementary Figure 16. Venn diagrams showing the variation partitioning of the genetic variation of *Trebouxia* photobiont explained by each group of explanatory variables based on dataset of Clade C (N=347) (A – mycobiont, B – climatic variable, C- altitude, D - geographical distances) a – genus of mycobiont, b – family of mycobiont, c – secondary metabolites composition.

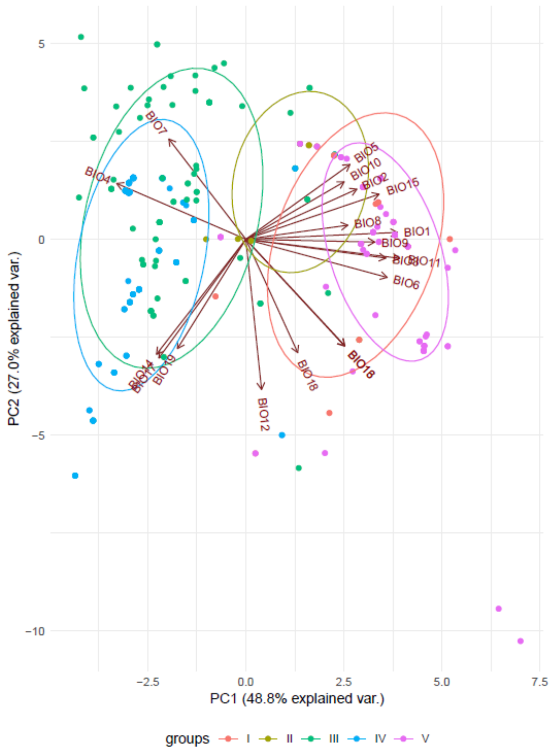

Supplementary Figure 17. PCA result of *Trebouxia* distribution depending on climatic factors and *Trebouxia* major clades, based on dataset of Clade I (N=378).

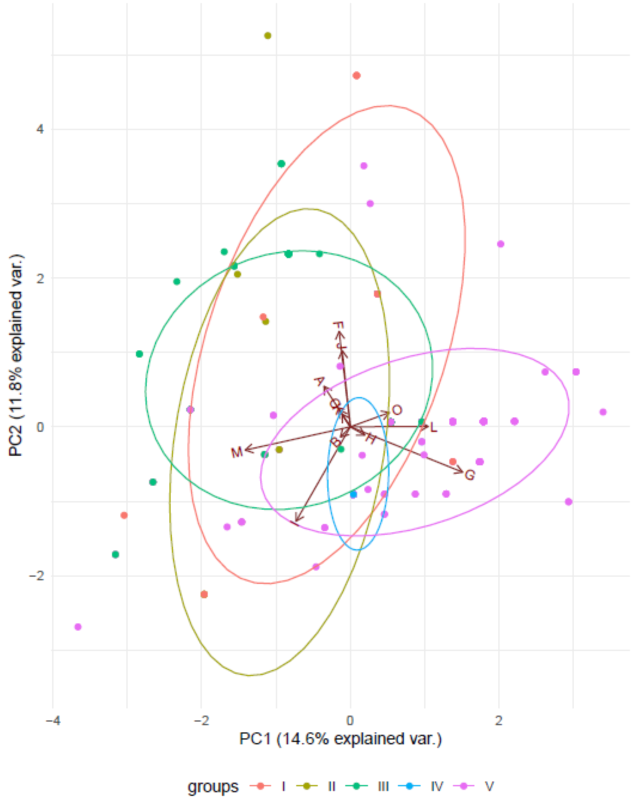

Supplementary Figure 18. PCA result of *Trebouxia* distribution depending on secondary metabolites factors and *Trebouxia* major clades, based on dataset of Clade I (N=378). No substances (A), presence of aliphatic (fatty) acids (B), anthraquinones (C), orcinol depsides (F), β-orcinol depsides (G), orcinol depsidones (H), β-orcinol depsidones (I), orcinol tridepsides (J), pulvinic acid derivatives (K), terpenoids (L), usnic acid derivatives (M), pigments (O).

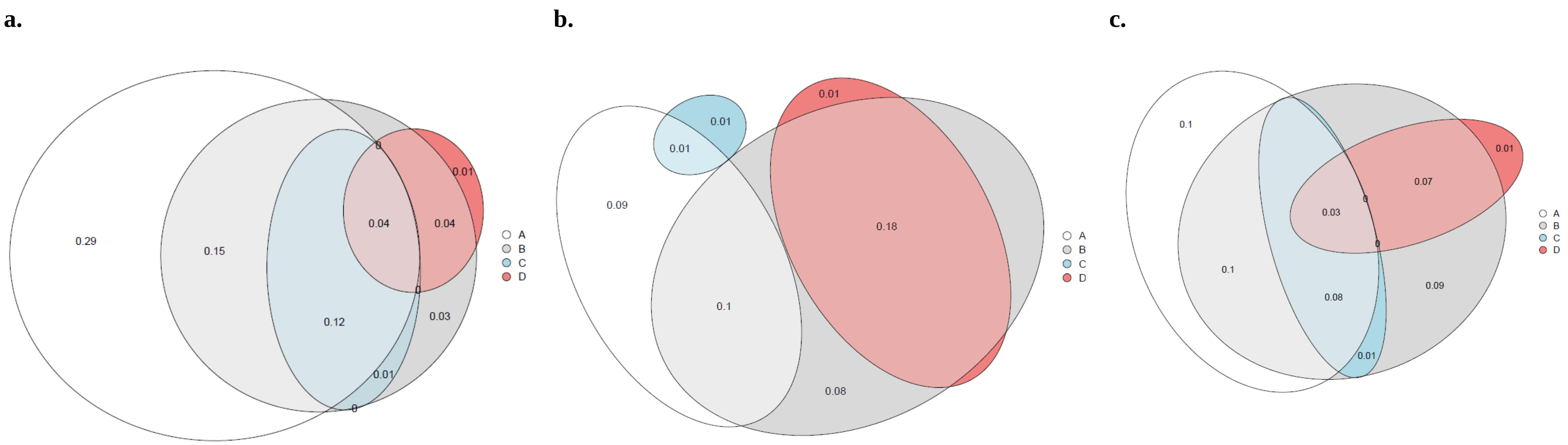

Supplementary Figure 19. Venn diagrams showing the variation partitioning of the genetic variation of *Trebouxia* photobiont explained by each group of explanatory variables based on dataset of Clade I (N=378) (A – mycobiont, B – climatic variable, C- altitude, D - geographical distances) a – genus of mycobiont, b – family of mycobiont, c – secondary metabolites composition.

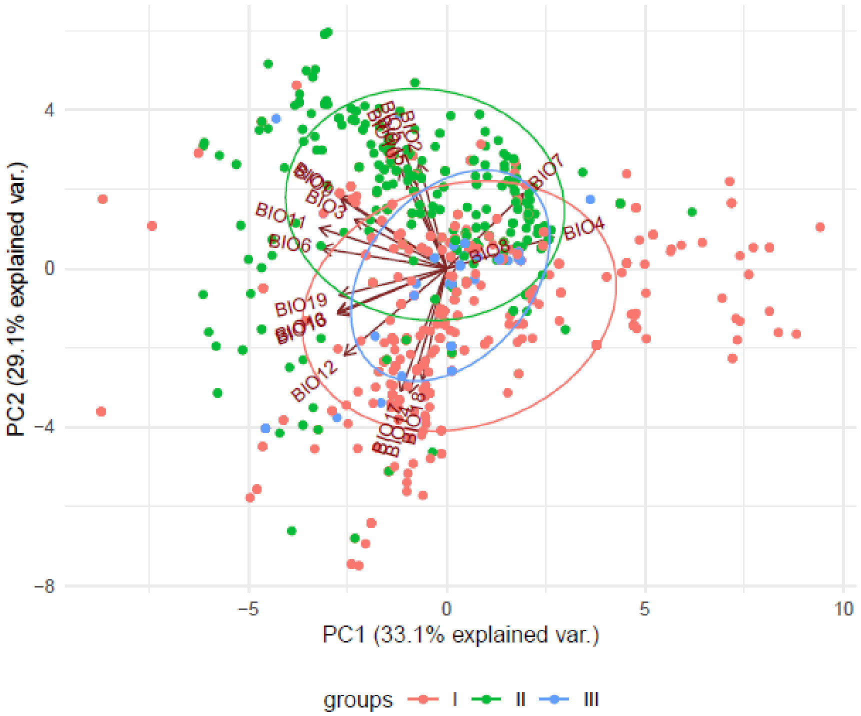

Supplementary Figure 20. PCA result of *Trebouxia* distribution depending on climatic factors and *Trebouxia* major clades, based on dataset of Clade S (N=1070).

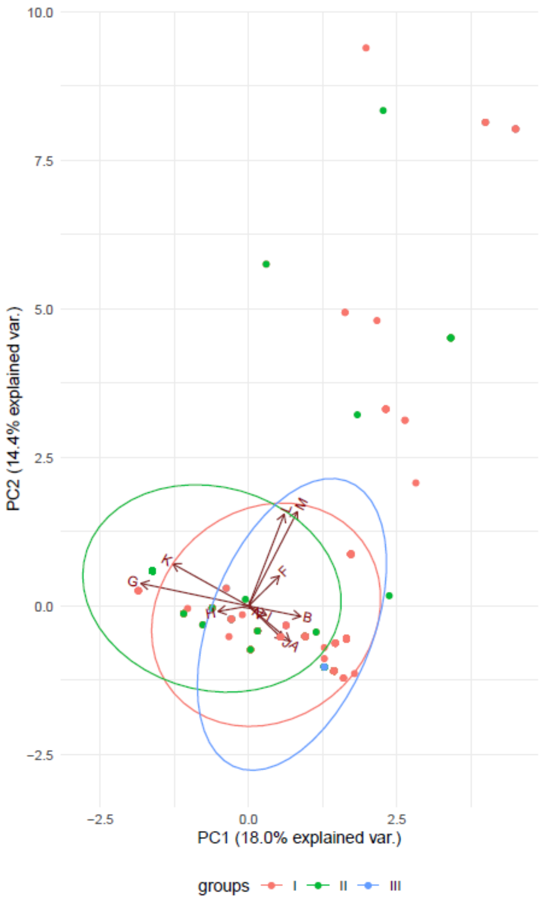

Supplementary Figure 21. PCA result of *Trebouxia* distribution depending on secondary metabolites factors and *Trebouxia* major clades, based on dataset of Clade S (N=1070). No substances (A). Presence of aliphatic (fatty) acids (B), orcinol depsides (F), β-orcinol depsides (G), orcinol depsidones (H), β-orcinol depsidones (I), orcinol tridepsides (J), pulvinic acid derivatives (K), terpenoids (L), usnic acid derivatives (M), xanthones (N).

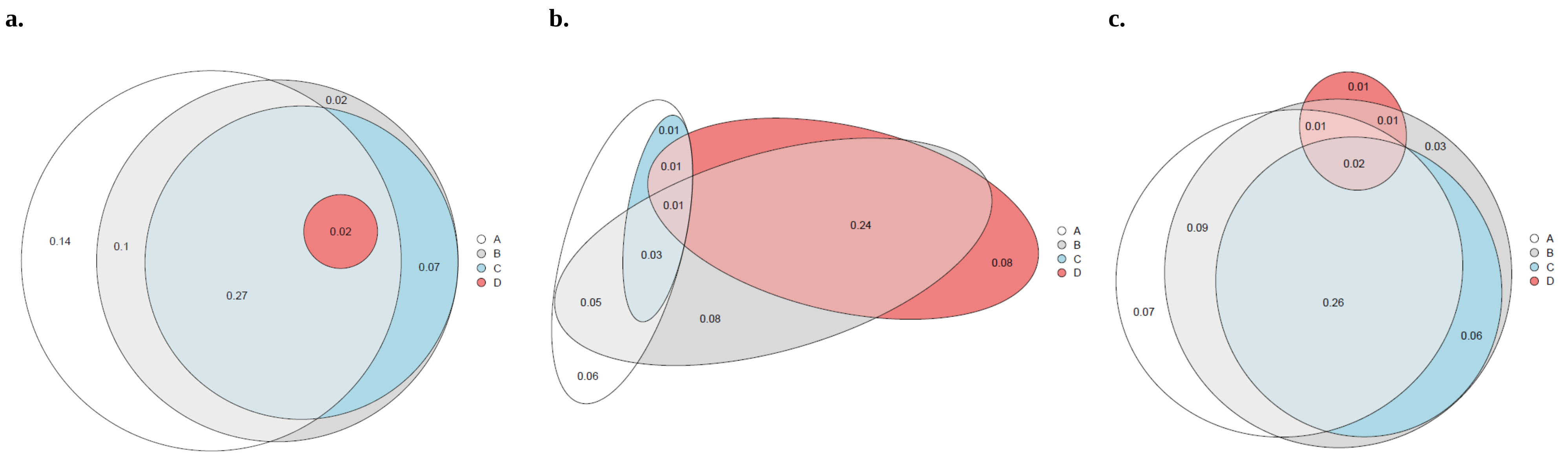

Supplementary Figure 22. Venn diagrams showing the variation partitioning of the genetic variation of *Trebouxia* photobiont explained by each group of explanatory variables based on dataset of Clade S (N=1070) (A – mycobiont, B – climatic variable, C- altitude, D - geographical distances) a – genus of mycobiont, b – family of mycobiont, c – secondary metabolites composition.

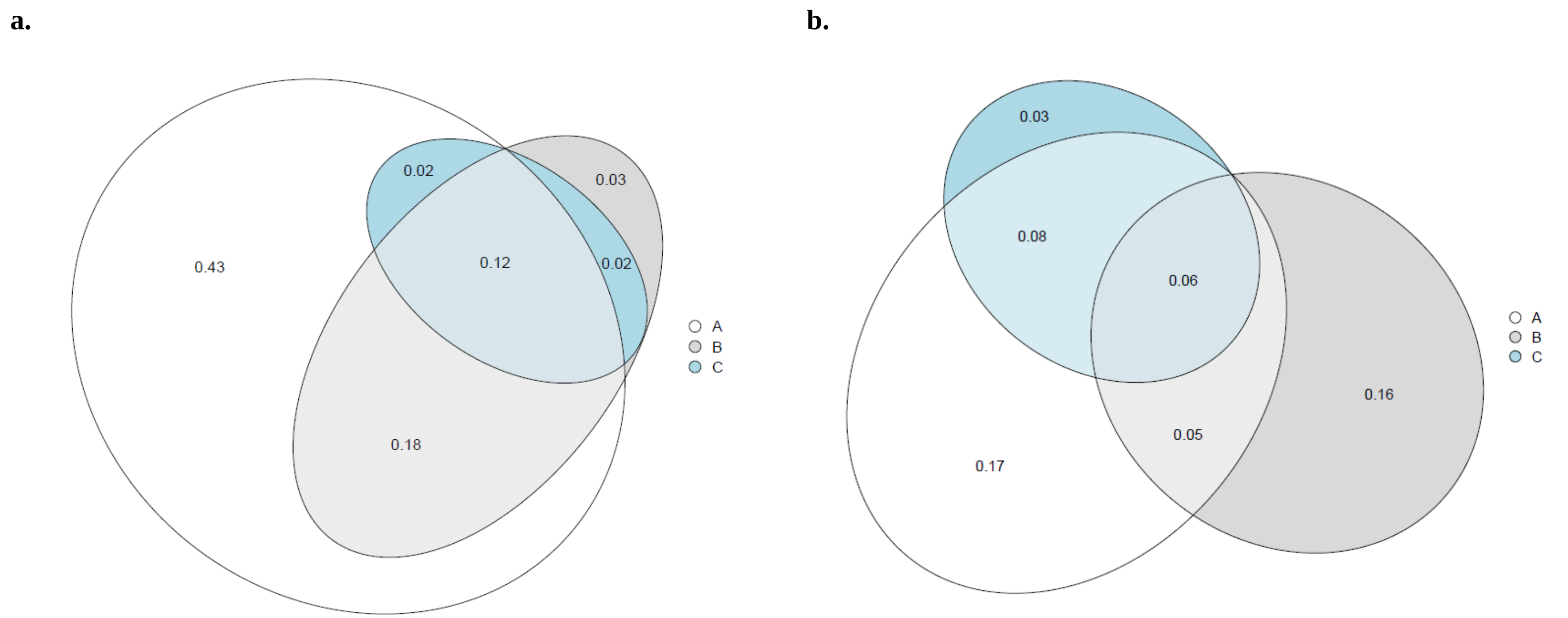

Supplementary Figure 23. Venn diagrams showing the variation partitioning of the genetic variation of *Trebouxia* photobiont explained by each group of explanatory variables based on dataset of *Lecanora* (N=140) (A – mycobiont, B – climatic variable, C - geographical distances) a – species of mycobiont, b – secondary metabolites composition.

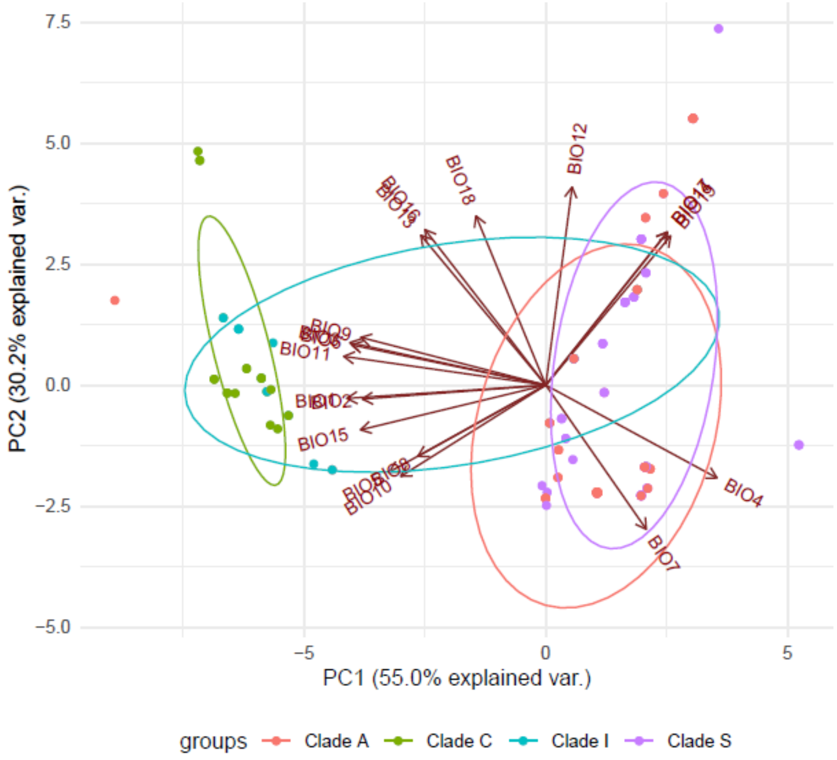

Supplementary Figure 24. PCA result of *Trebouxia* distribution depending on climatic factors and *Trebouxia* major clades, based on dataset of *Lecanora* (N=140).

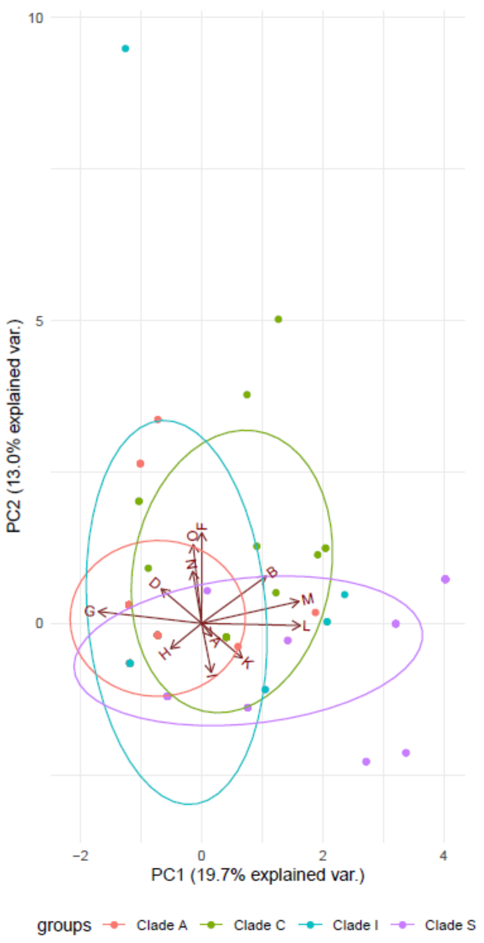

Supplementary Figure 25. PCA result of *Trebouxia* distribution depending on secondary metabolites factors and *Trebouxia* major clades, based on dataset of *Lecanora* (N=140). No substances (A). Presence of aliphatic (fatty) acids (B), ergochromes (D), orcinol depsides (F), β-orcinol depsides (G), orcinol depsidones (H), β-orcinol depsidones (I), pulvinic acid derivatives (K), terpenoids (L), usnic acid derivatives (M), xanthones (N), pigments (O).

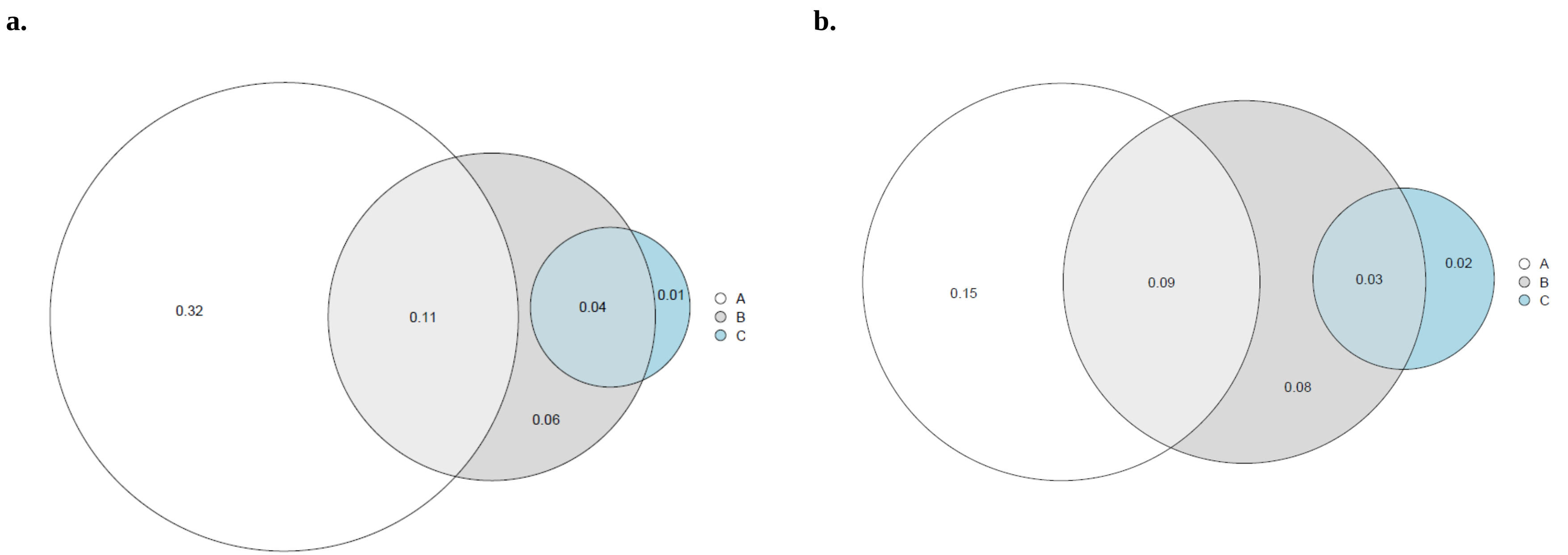

Supplementary Figure 26. Venn diagrams showing the variation partitioning of the genetic variation of *Trebouxia* photobiont explained by each group of explanatory variables based on dataset of *Lecidea* (N=100) (A – mycobiont, B – climatic variable, C - geographical distances) a – species of mycobiont, b – secondary metabolites composition.

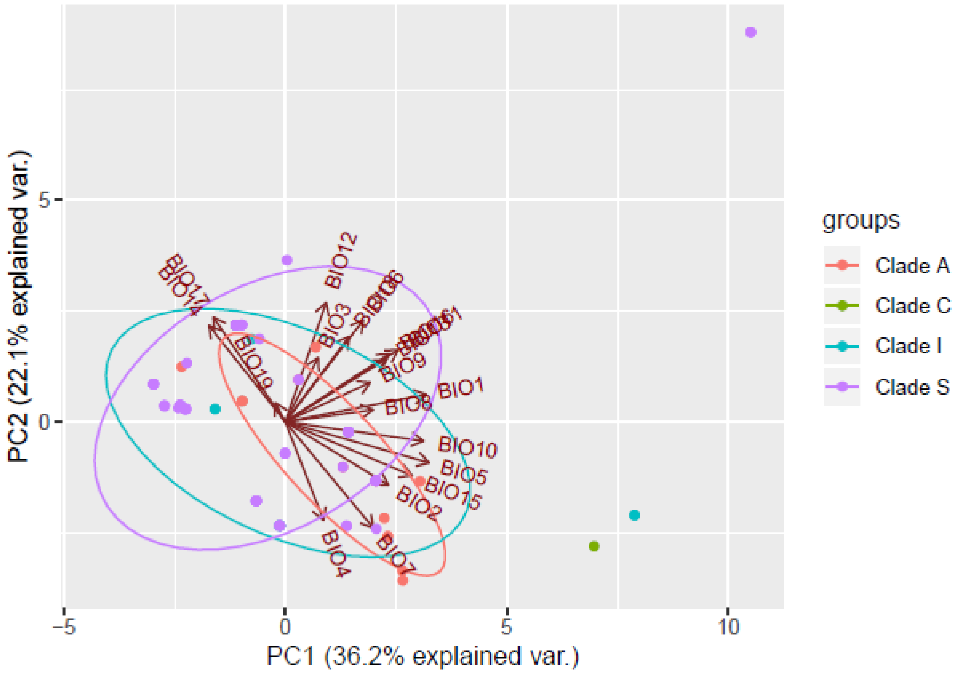

Supplementary Figure 27. PCA result of *Trebouxia* distribution depending on climatic factors and *Trebouxia* major clades, based on dataset of *Lecidea* (N=100).

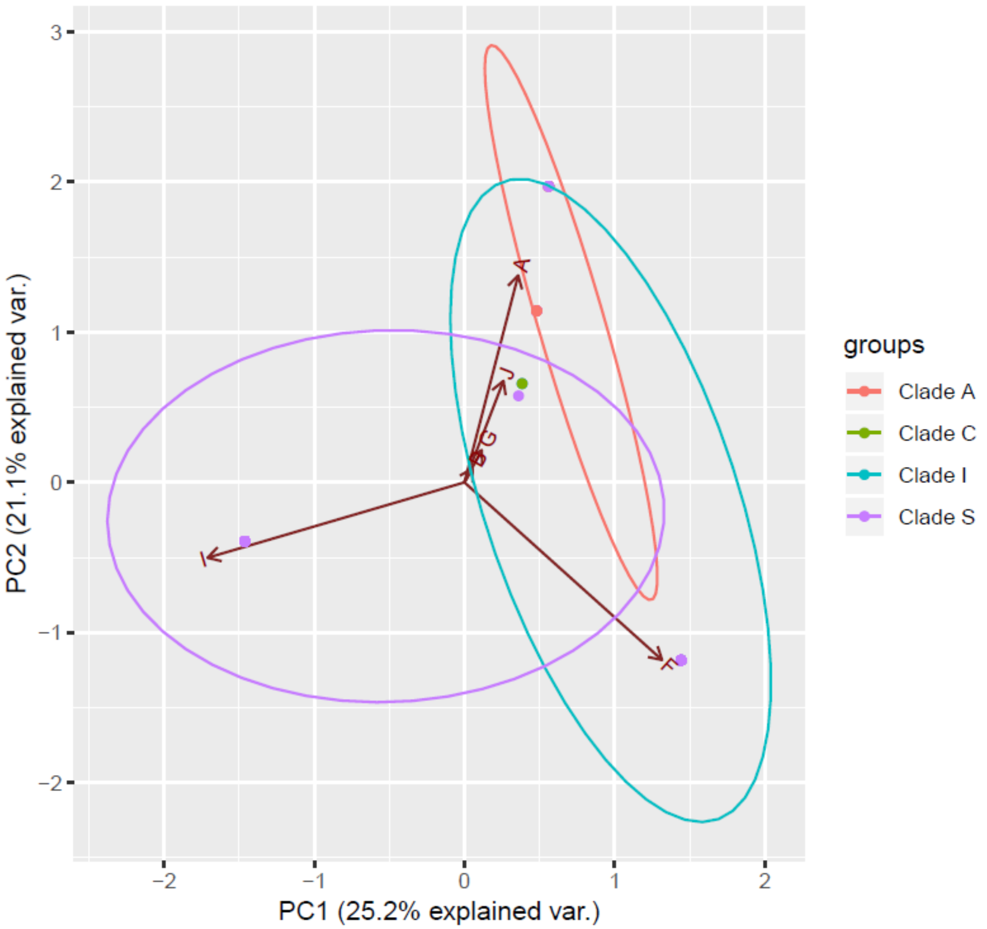

Supplementary Figure 28. PCA result of *Trebouxia* distribution depending on secondary metabolites factors and *Trebouxia* major clades, based on dataset of *Lecidea* (N=100). No substances (A). Presence of aliphatic (fatty) acids (B), orcinol depsides (F), β-orcinol depsides (G), β-orcinol depsidones (I), orcinol tridepsides (J).

a. **b.**

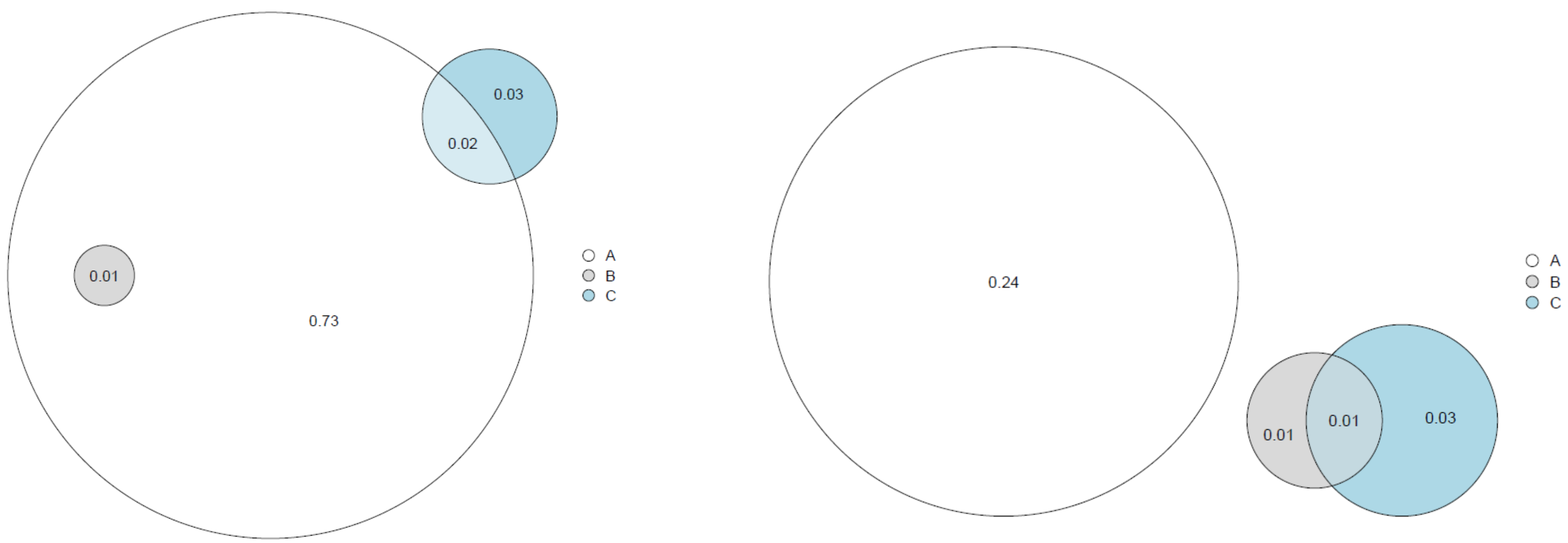

Supplementary Figure 29. Venn diagrams showing the variation partitioning of the genetic variation of *Trebouxia* photobiont explained by each group of explanatory variables based on dataset of *Hypotrachyna* (N=60) (A – mycobiont, B – climatic variable, C - geographical distances) a – species of mycobiont, b – secondary metabolites composition.

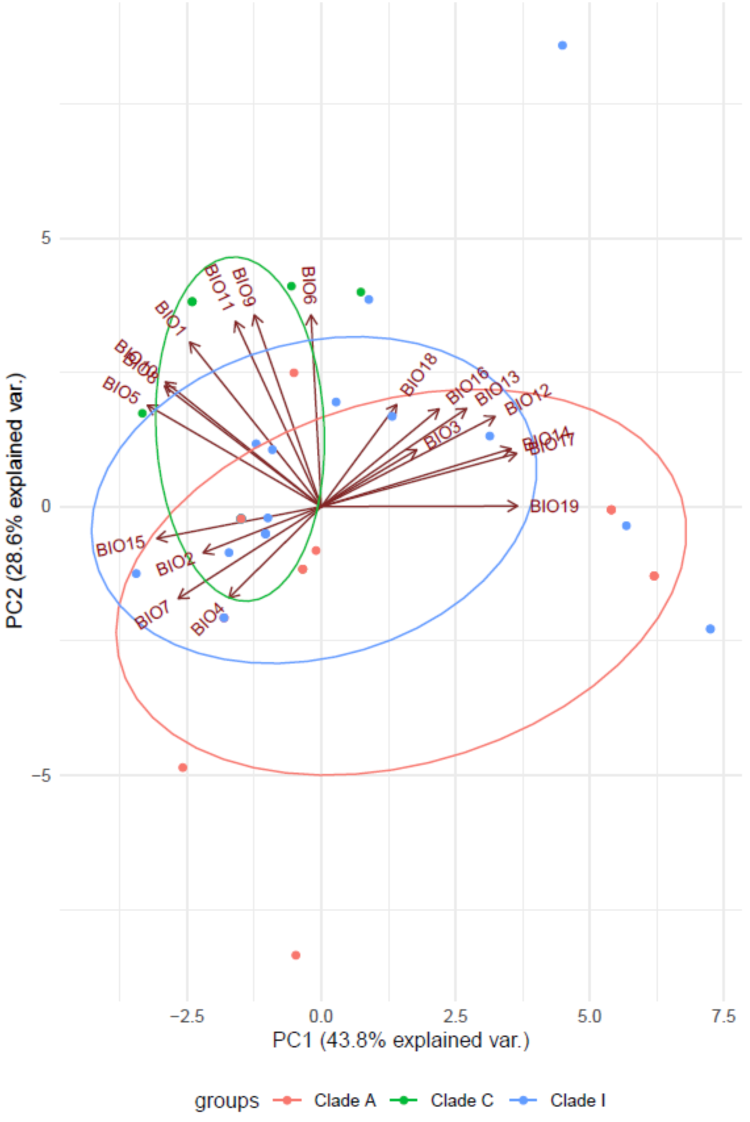

Supplementary Figure 30. PCA result of *Trebouxia* distribution depending on climatic factors and *Trebouxia* major clades, based on dataset of *Hypotrachyna* (N=60).

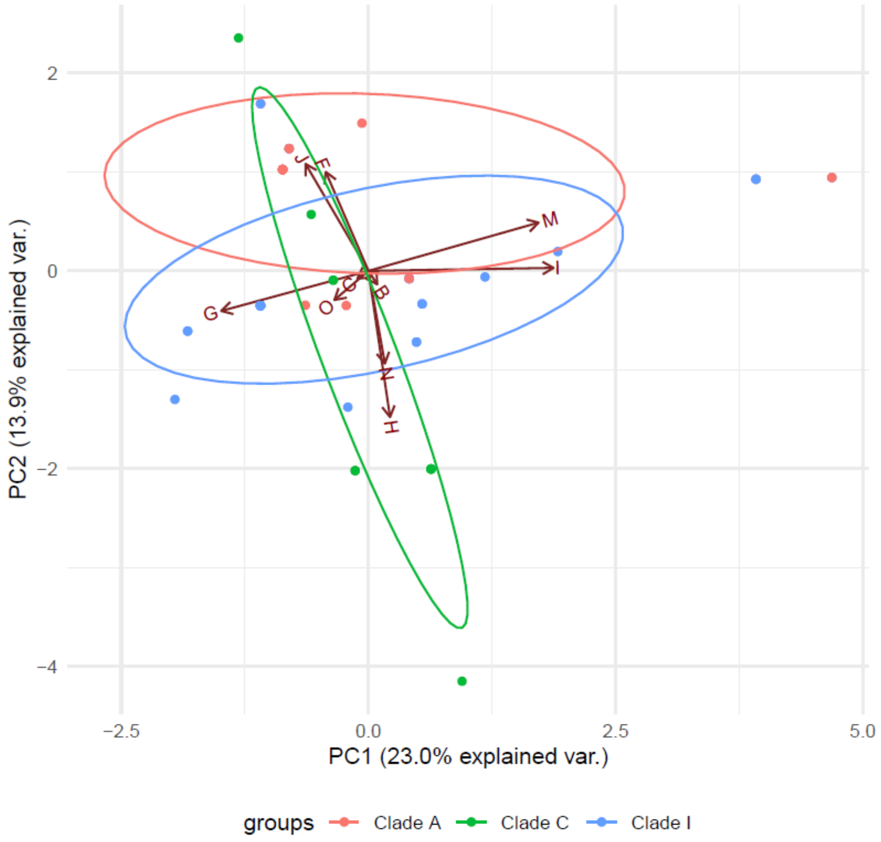

Supplementary Figure 31. PCA result of *Trebouxia* distribution depending on secondary metabolites factors and *Trebouxia* major clades, based on dataset of *Hypotrachyna* (N=60). Presence of aliphatic (fatty) acids (B), anthraquinones (C), ergochromes (D), orcinol depsides (F), β-orcinol depsides (G), orcinol depsidones (H), β-orcinol depsidones (I), orcinol tridepsides (J), pulvinic acid derivatives (K), terpenoids (L), usnic acid derivatives (M), xanthones (N), pigments (O).

a.

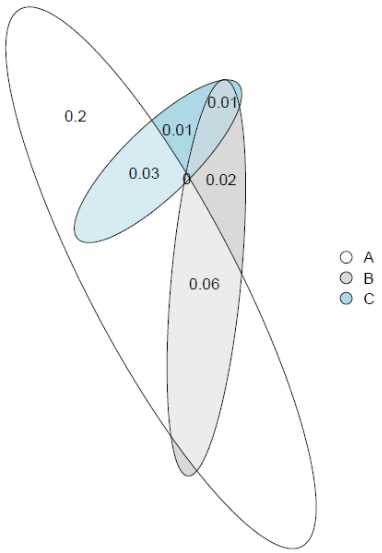

b.

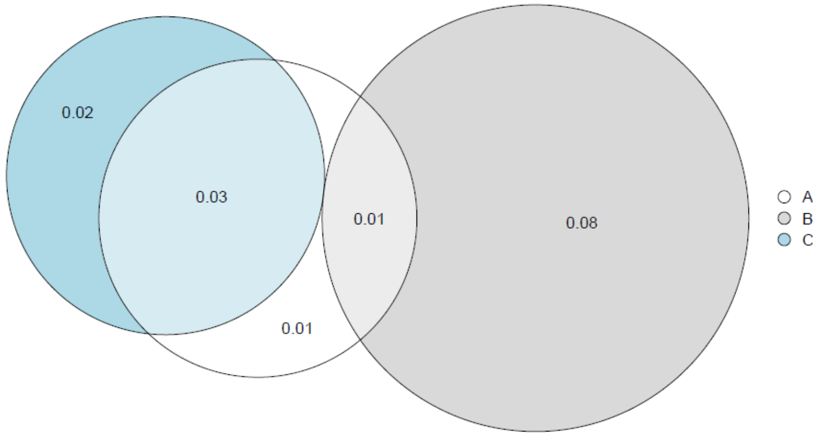

Supplementary Figure 32. Venn diagrams showing the variation partitioning of the genetic variation of *Trebouxia* photobiont explained by each group of explanatory variables based on dataset of *Parmotrema* (N=137) (A – mycobiont, B – climatic variable, C - geographical distances) a – species of mycobiont, b – secondary metabolites composition.

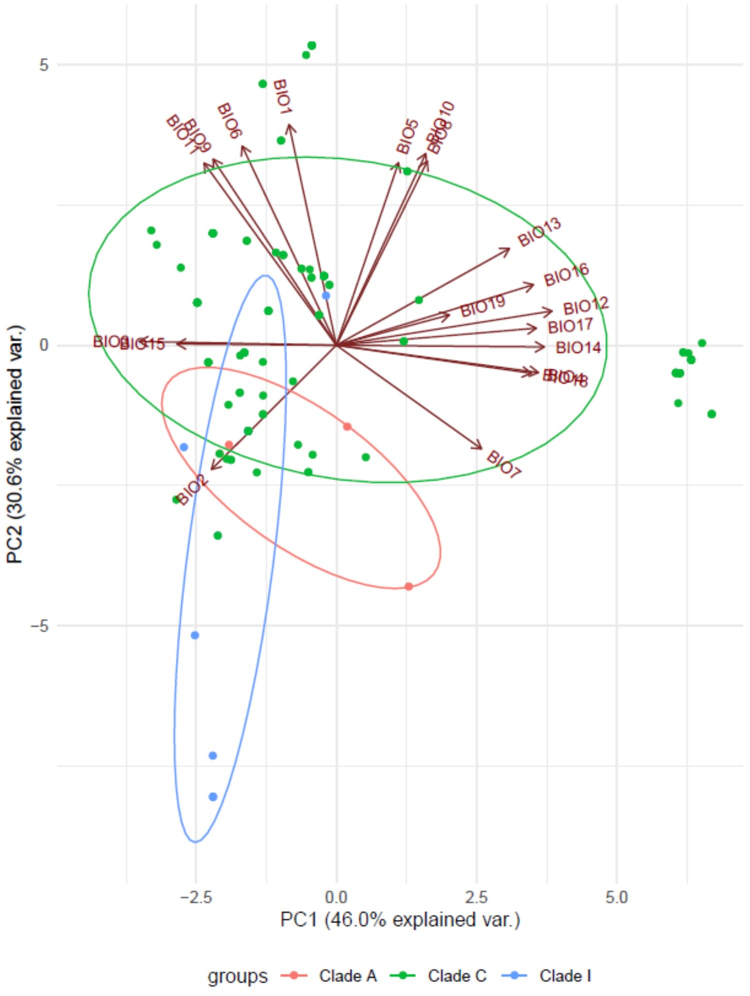

Supplementary Figure 33. PCA result of *Trebouxia* distribution depending on climatic factors and *Trebouxia* major clades, based on dataset of *Parmotrema* (N=137).

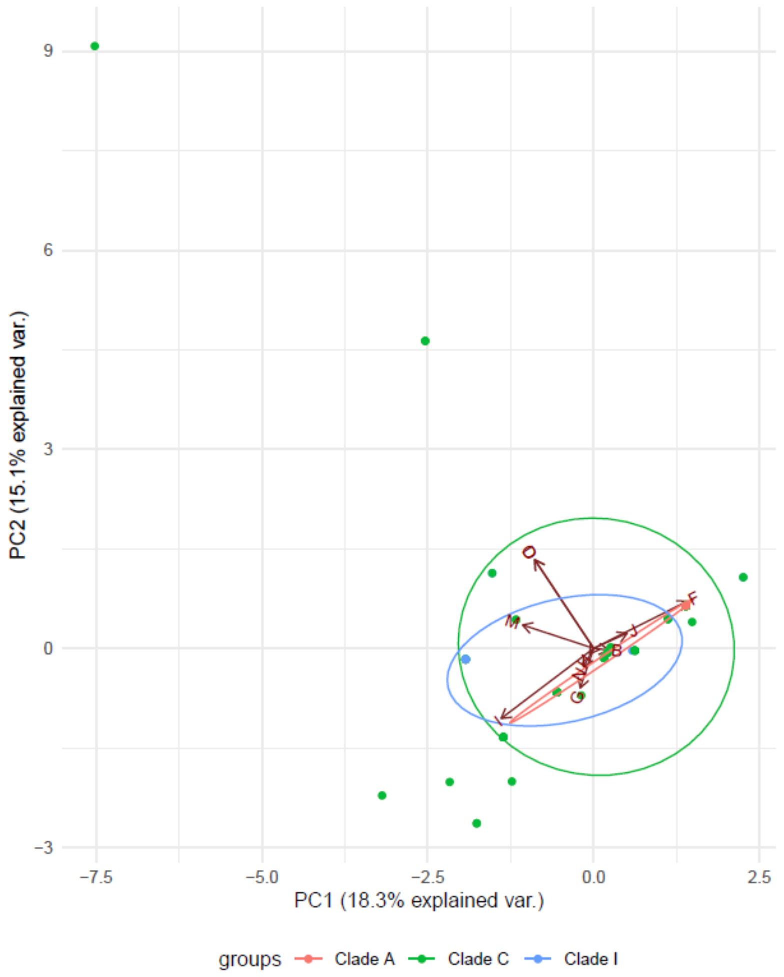

Supplementary Figure 34. PCA result of *Trebouxia* distribution depending on secondary metabolites factors and *Trebouxia* major clades, based on dataset of *Parmotrema* (N=137). No substances (A), presence of aliphatic (fatty) acids (B), ergochromes (D), orcinol depsides (F), β-orcinol depsides (G), orcinol depsidones (H), β-orcinol depsidones (I), orcinol tridepsides (J), pulvinic acid derivatives (K), terpenoids (L), usnic acid derivatives (M), xanthones (N), pigments (O).

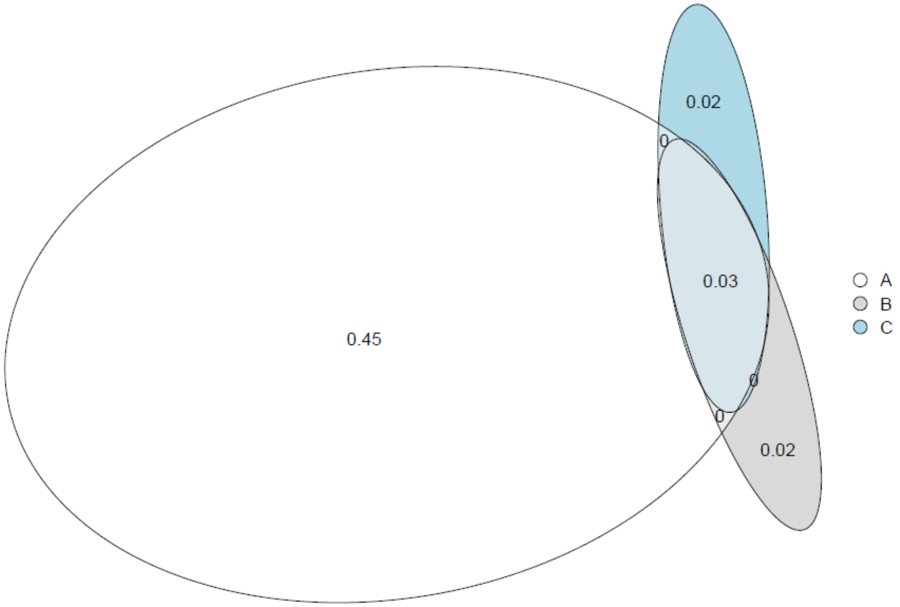

Supplementary Figure 35. Venn diagram showing the variation partitioning of the genetic variation of *Trebouxia* photobiont explained by each group of explanatory variables based on dataset of *Usnea* (N=51) (A – species of mycobiont, B – climatic variable, C - geographical distances).

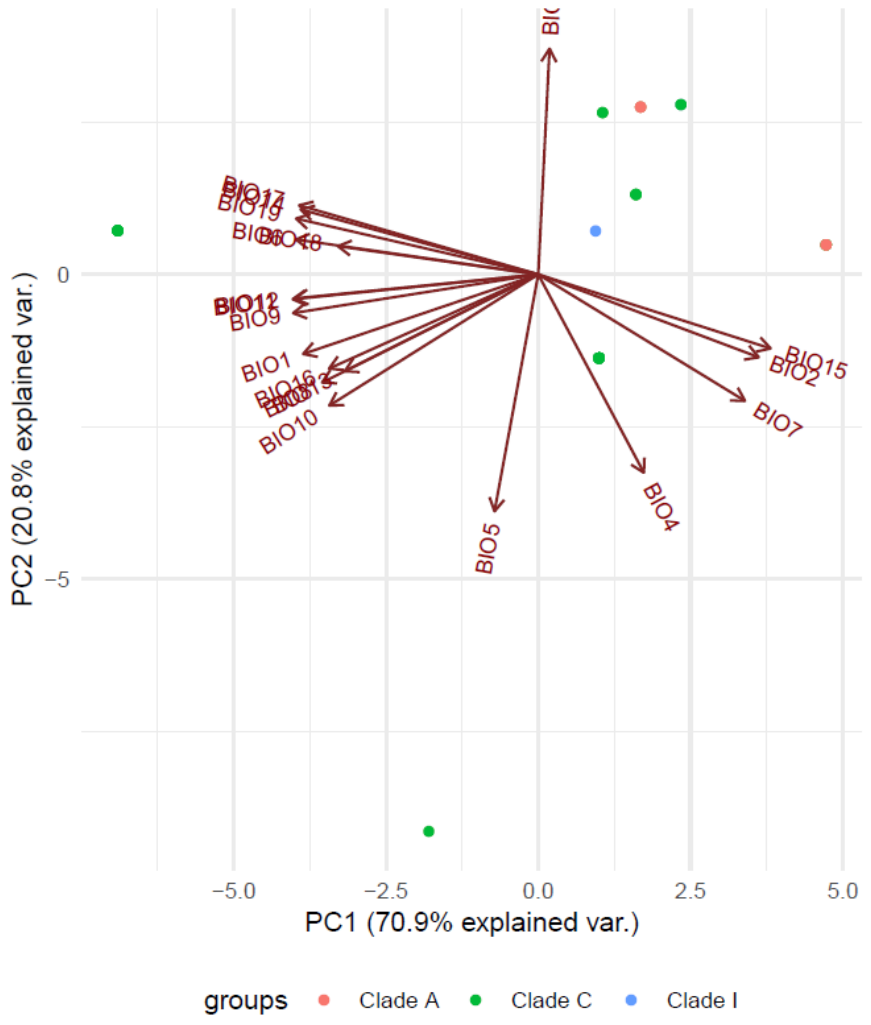

Supplementary Figure 36. PCA result of *Trebouxia* distribution depending on climatic factors and *Trebouxia* major clades, based on dataset of *Usnea* (N=51).

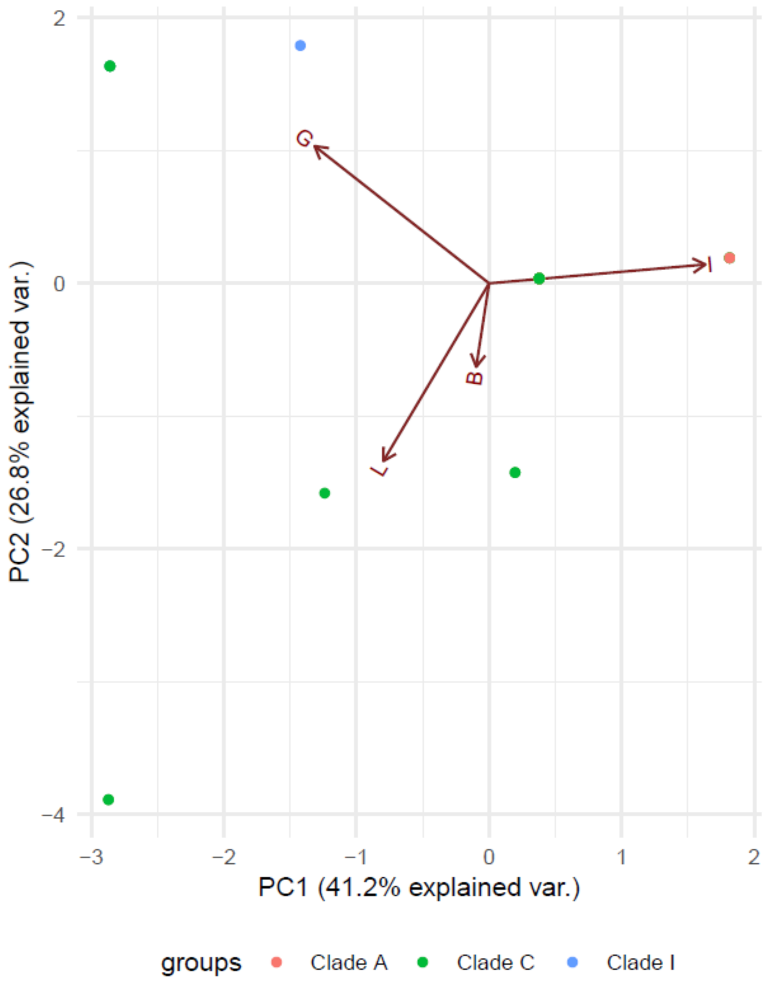

Supplementary Figure 37. PCA result of *Trebouxia* distribution depending on secondary metabolites factors and *Trebouxia* major clades, based on dataset of *Usnea* (N=51). Presence of aliphatic (fatty) acids (B), β-orcinol depsides (G), β-orcinol depsidones (I), terpenoids (L).

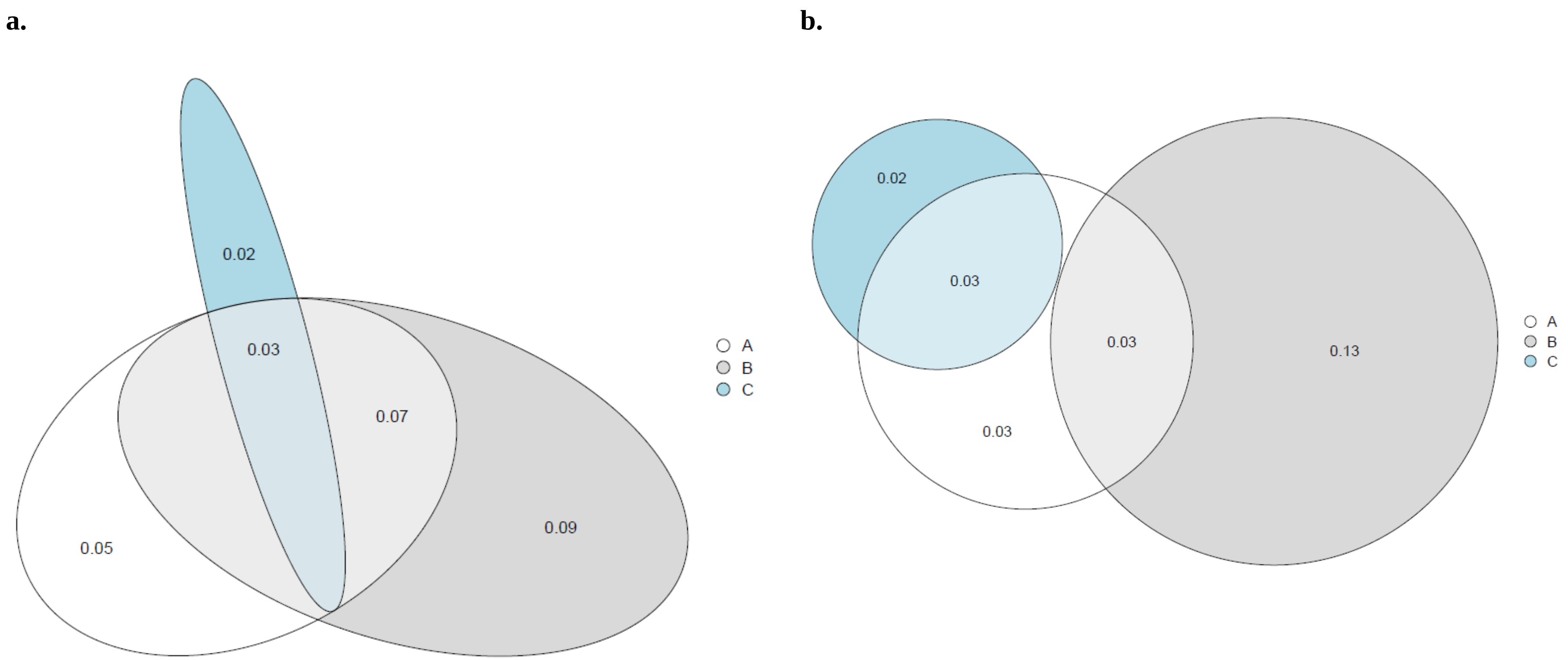

Supplementary Figure 38. Venn diagrams showing the variation partitioning of the genetic variation of *Trebouxia* photobiont explained by each group of explanatory variables based on dataset of *Xanthoparmelia* (N=431) (A – mycobiont, B – climatic variable, C - geographical distances) a – species of mycobiont, b – secondary metabolites composition.

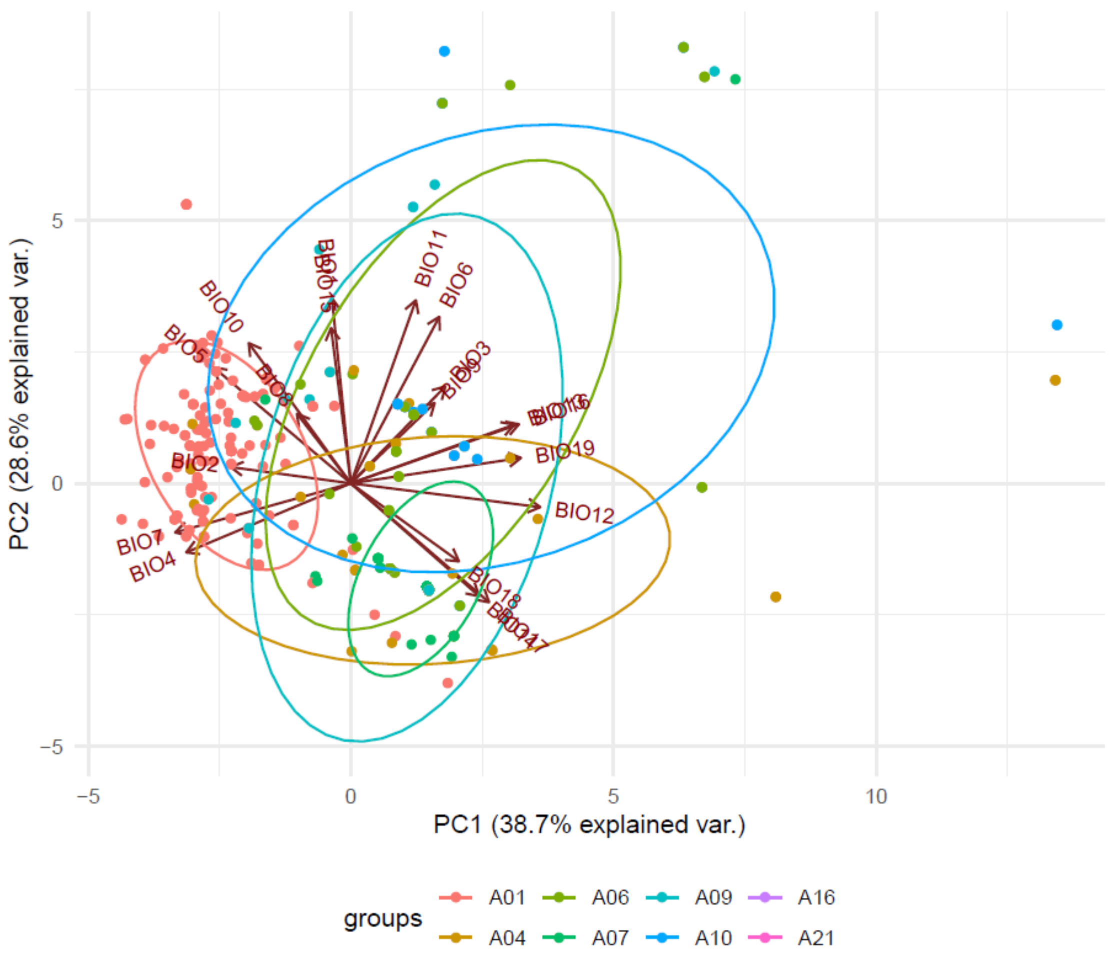

Supplementary Figure 39. PCA result of *Trebouxia* distribution depending on climatic factors and *Trebouxia* OTUs from clade A, based on dataset of *Xanthoparmelia* (N=431).

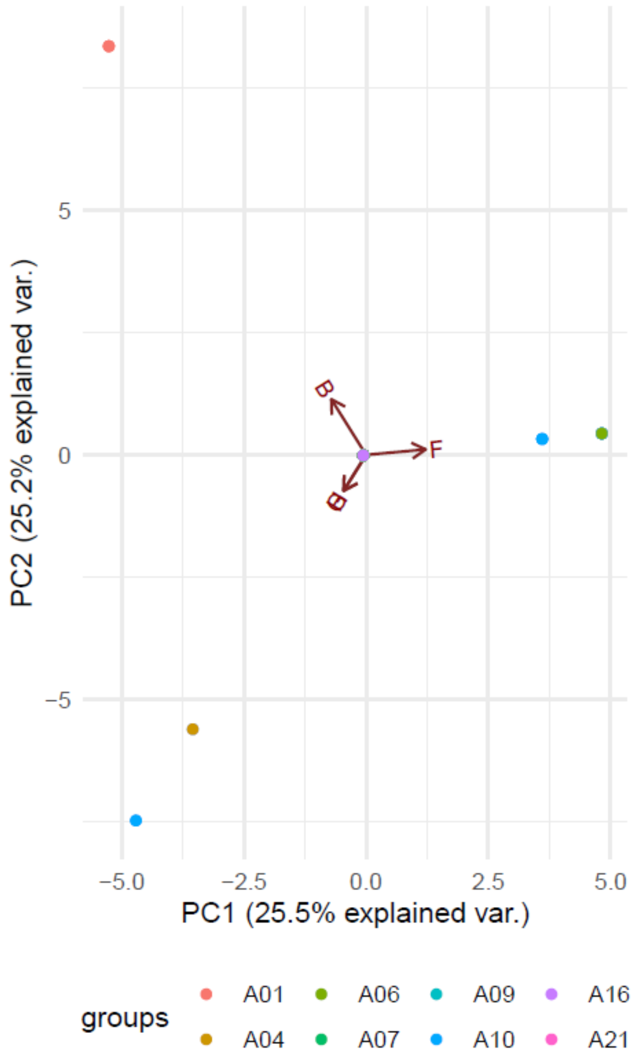

Supplementary Figure 40. PCA result of *Trebouxia* distribution depending on secondary metabolites factors and *Trebouxia* OTUs from clade A, based on dataset of *Xanthoparmelia* (N=431). Presence of aliphatic (fatty) acids (B), orcinol depsides (F), orcinol depsidones (H), β-orcinol depsidones (I).

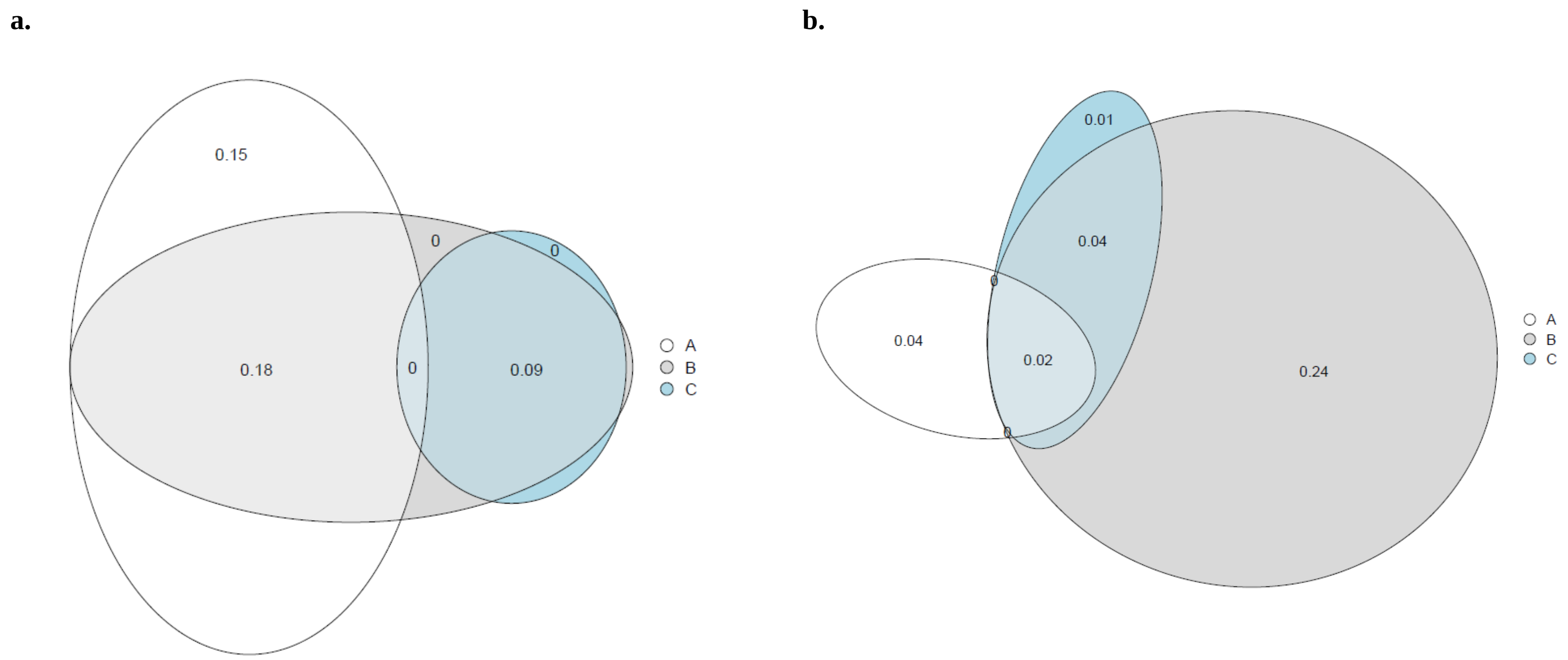

Supplementary Figure 41. Venn diagrams showing the variation partitioning of the genetic variation of *Trebouxia* photobiont explained by each group of explanatory variables based on dataset of *Polyblastidium* (N=28) (A – mycobiont, B – climatic variable, C - geographical distances) a – species of mycobiont, b – secondary metabolites composition.

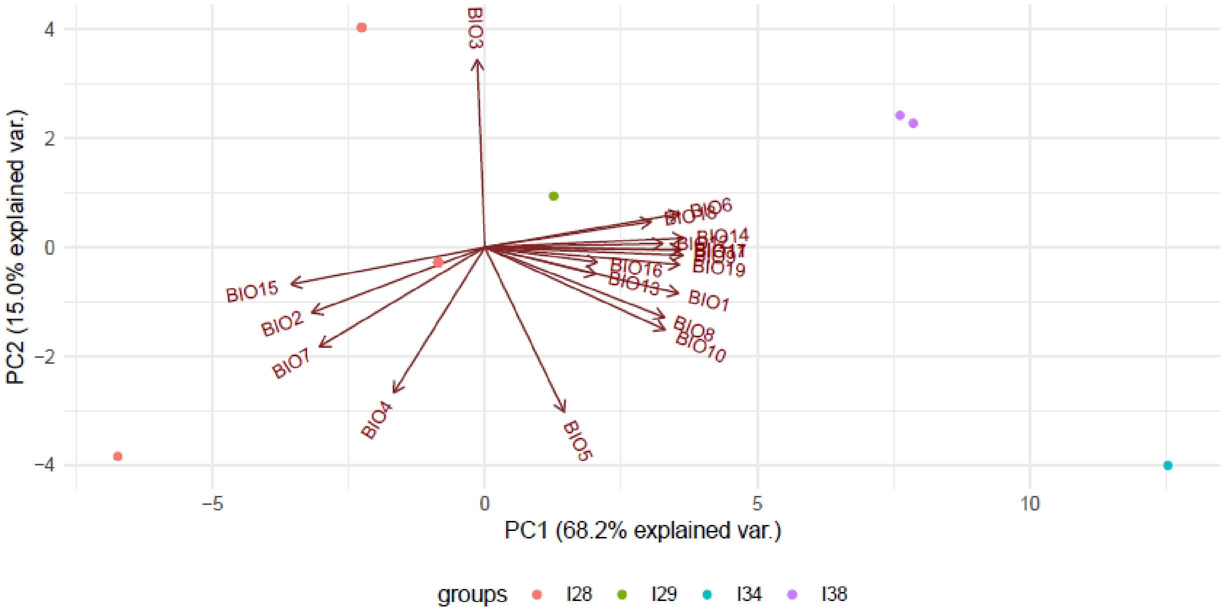

Supplementary Figure 42. PCA result of *Trebouxia* distribution depending on climatic factors and *Trebouxia* major clades, based on dataset of *Polyblastidium* (N=28).

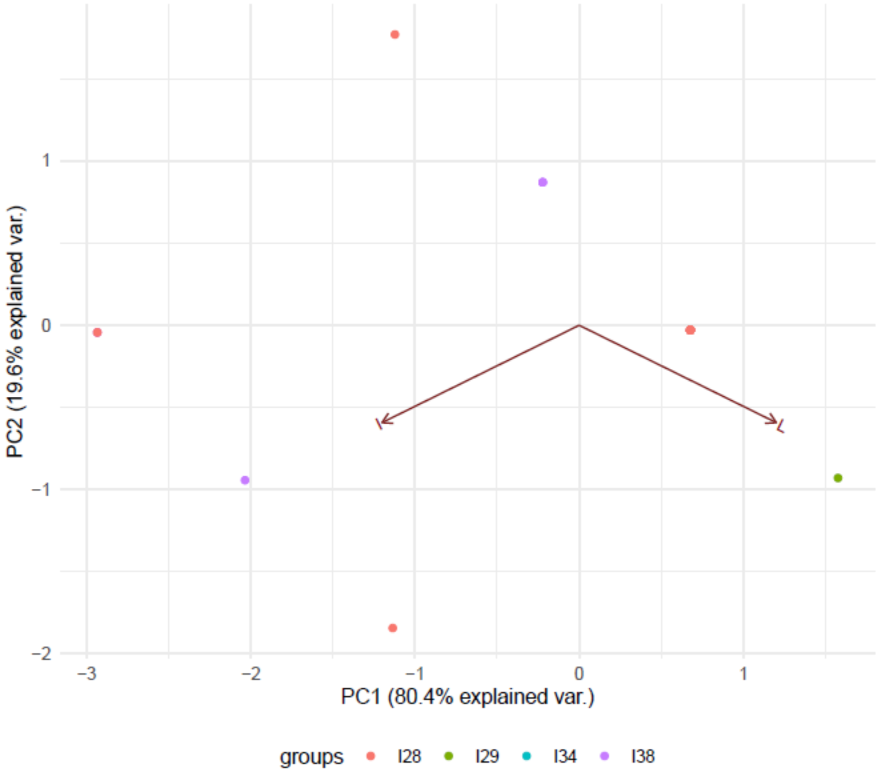

Supplementary Figure 43. PCA result of *Trebouxia* distribution depending on secondary metabolites factors and *Trebouxia* major clades, based on dataset of *Polyblastidium* (N=28). Presence of β-orcinol depsidones (I), terpenoids (L).

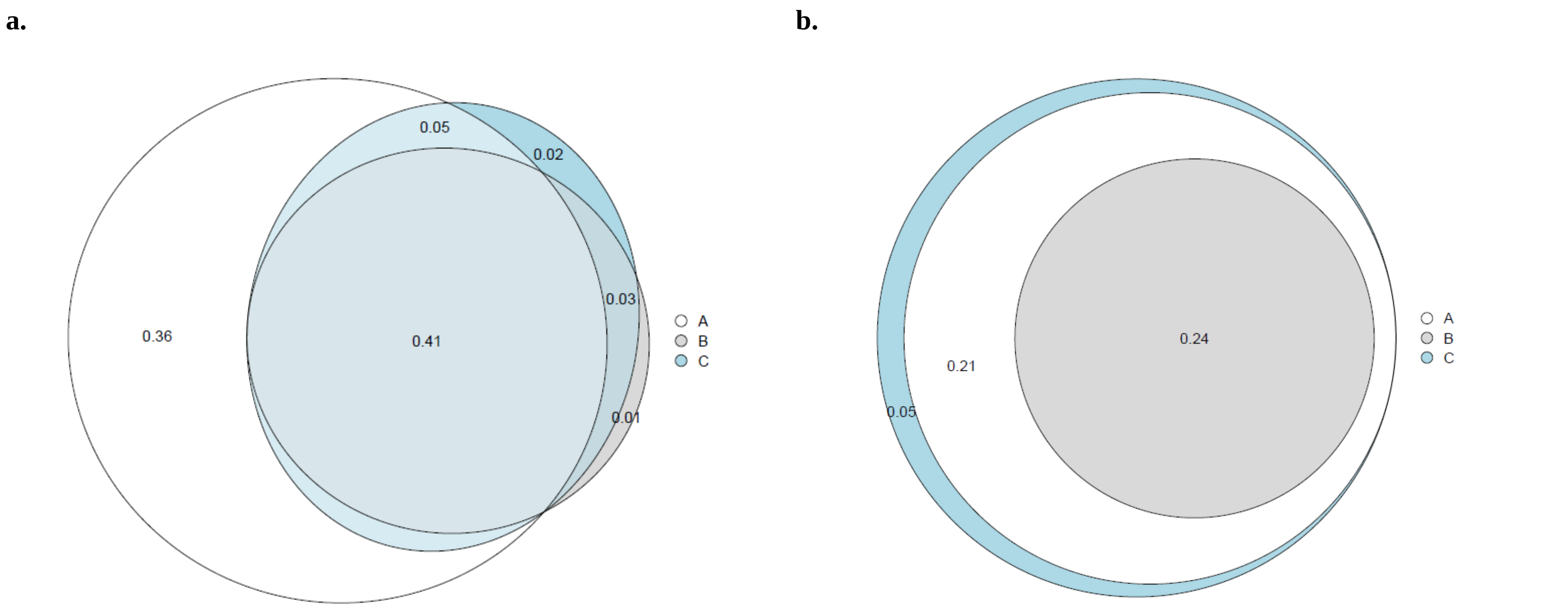

Supplementary Figure 44. Venn diagrams showing the variation partitioning of the genetic variation of *Trebouxia* photobiont explained by each group of explanatory variables based on dataset of *Pertusaria* (N=33) (A – mycobiont, B – climatic variable, C - geographical distances) a – species of mycobiont, b – secondary metabolites composition.

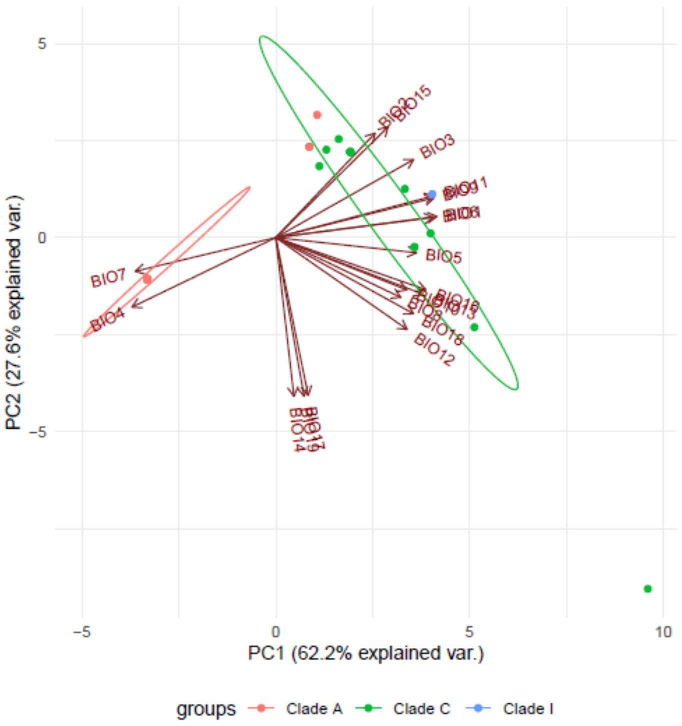

Supplementary Figure 45. PCA result of *Trebouxia* distribution depending on climatic factors and *Trebouxia* major clades, based on dataset of *Pertusaria* (N=33).

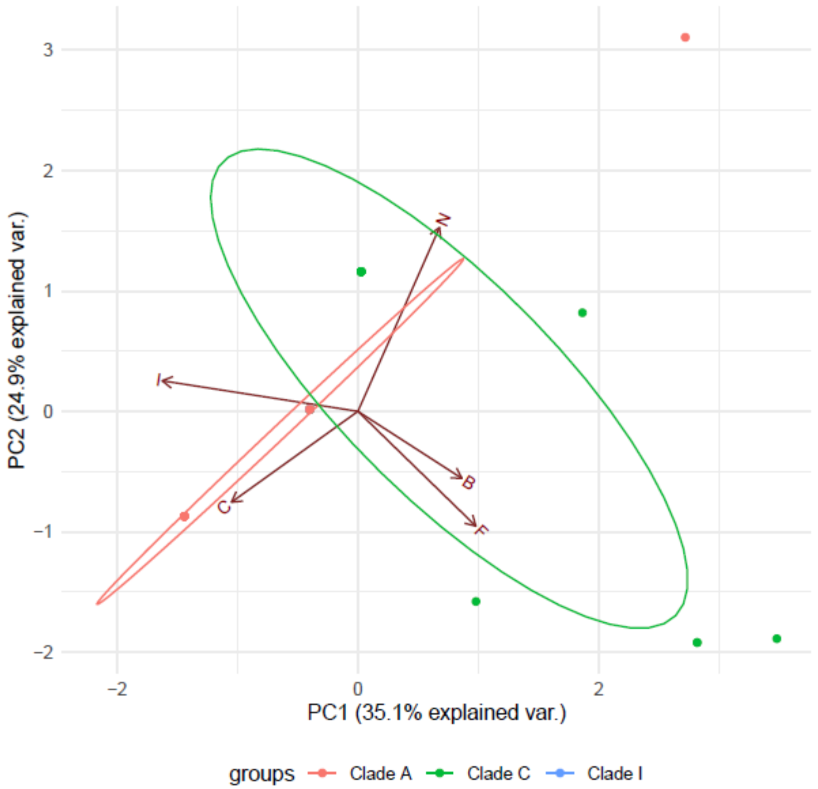

Supplementary Figure 46. PCA result of *Trebouxia* distribution depending on secondary metabolites factors and *Trebouxia* major clades, based on dataset of *Pertusaria* (N=33). Presence of aliphatic (fatty) acids (B), anthraquinones (C), orcinol depsides (F), β-orcinol depsidones (I), xanthones (N).

a. **b.**

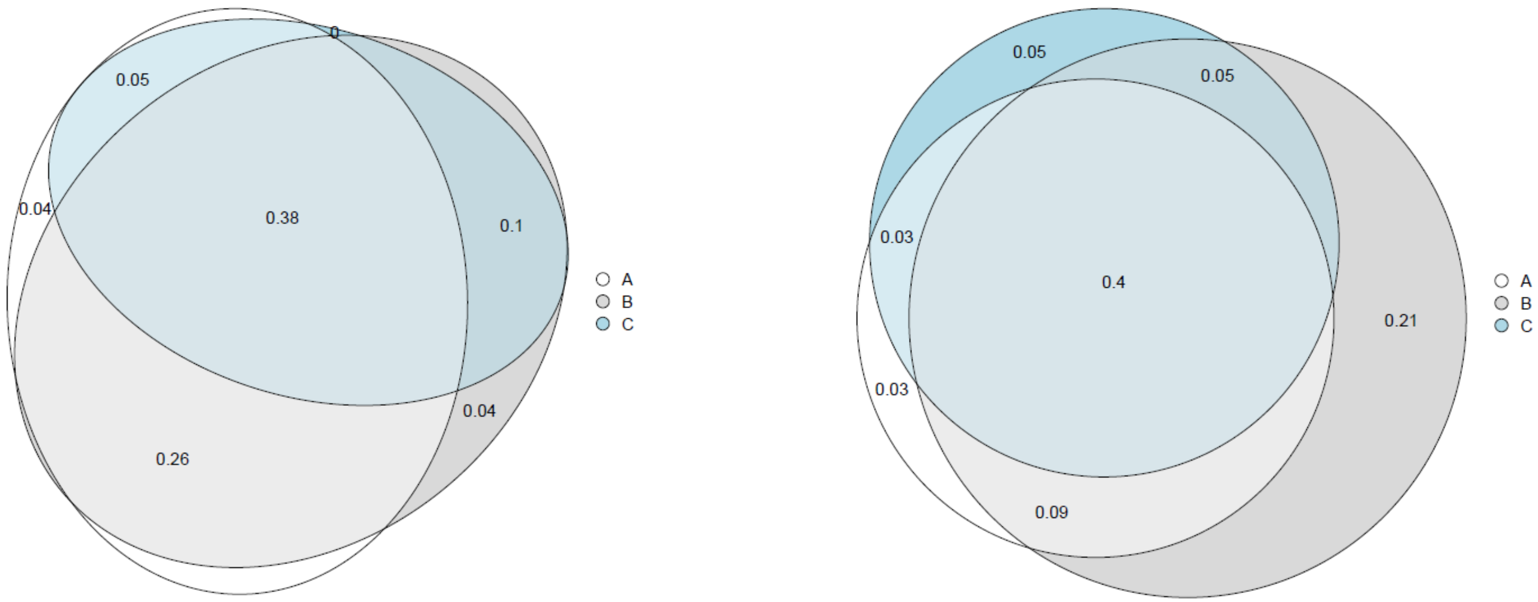

Supplementary Figure 47. Venn diagrams showing the variation partitioning of the genetic variation of *Trebouxia* photobiont explained by each group of explanatory variables based on dataset of *Lepra* (N=33) (A – mycobiont, B – climatic variable, C - geographical distances) a – species of mycobiont, b – secondary metabolites composition.

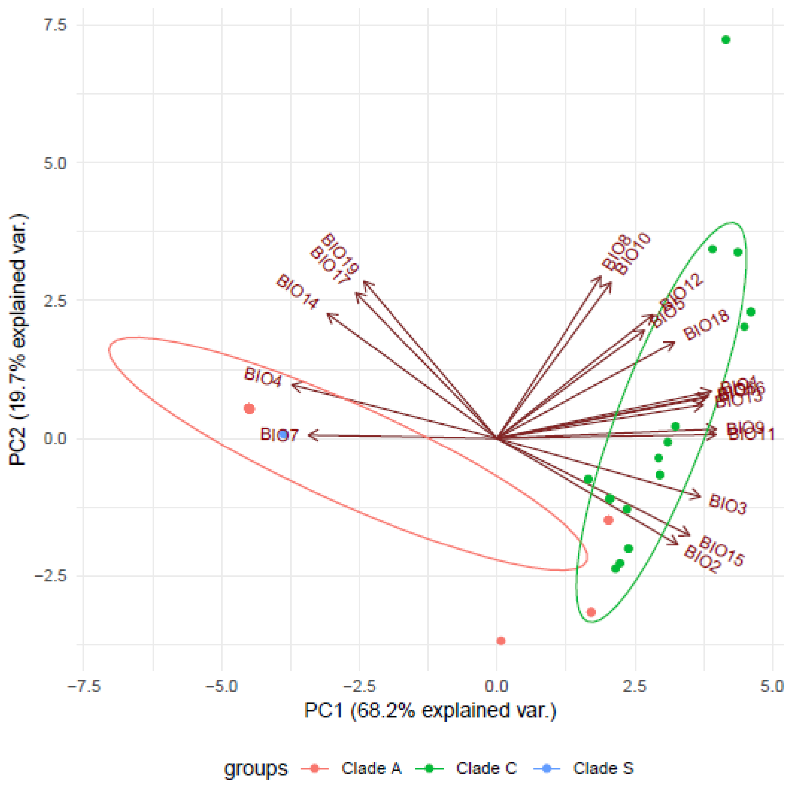

Supplementary Figure 48. PCA result of *Trebouxia* distribution depending on climatic factors and *Trebouxia* major clades, based on dataset of *Lepra* (N=33).

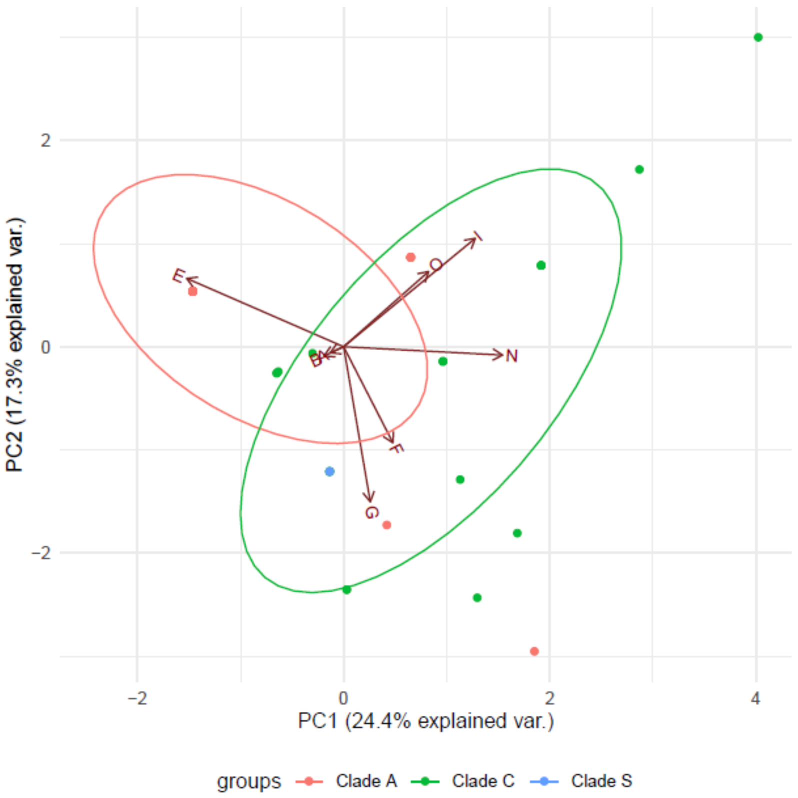

Supplementary Figure 49. PCA result of *Trebouxia* distribution depending on secondary metabolites factors and *Trebouxia* major clades, based on dataset of *Lepra* (N=33). Presence of aliphatic (fatty) acids (B), depsones (E), orcinol depsides (F), β-orcinol depsides (G), β-orcinol depsidones (I), xanthones (N).

1.2 Supplementary Tables

Supplementary Table 1. List of specimens from which photobiont sequences were newly obtained with their ITS rDNA and *rbc*L gene GenBank accession numbers. All samples were collected in Bolivia and voucher specimens are deposited in herbaria LPB and UGDA (Excel file).

Supplementary Table 2. The PCR condition used in this study to amplify the ITS rDNA region, the chloroplast *rbc*L marker.

Region		ITS rDNA region (algal)		chloroplast *rbc*L gene	ITS rDNA region (fungal)
Primer name		ITS1T and ITS4T (Kroken & Taylor, 2000)	AL1500bf (Helms et al. 2001) and ITS4M (Guzow-Krzemińska 2006)	PRASF1 (Nozaki et al. 1995) and a-ch-rbcL-991-30-MPN (Nelsen et al. 2011)	ITS1F (Gardes and Bruns, 1993) and ITS4A (Kroken and Taylor 2001)
initial denaturation		94°C for 2 min	95°C for 2 min	95°C for 2 min	94°C for 3 min
35 cycles	denaturation	95°C for 30 s	95°C for 45 s	95°C for 1 min	94°C for 30 s
	annealing	56°C for 30 s	51°C for 40 s	60°C for 1 min	54°C for 30 s
	elongation	72°C for 1 min	72°C for 1 min 20 s	72°C for 1 min	72°C for 1 min
final extension		72°C for 5 min	72°C for 5 min	72°C for 7 min	72°C for 10 min

Supplementary Table 3. Sequences of ITS rDNA, *rbc*L and *cox2* markers of *Trebouxia* downloaded from GeneBank and used in this study in phylogenetic inference (Excel file).

Supplementary Table 4. Summary of occurrence of particular *Trebouxia* lineages in selected habitat types in Bolivia. In proper color are marked lineages that were observed in particular habitat – red for open high Andean vegetation, orange for upper montane cloud forest part 1, yellow for upper montane cloud forest 1, and green for lower montane cloud forest. Lineages that were identified in more than one habitat type are marked in grey (Excel file).

Supplementary Table 5. Results of distance-based redundancy analyzes (dbRDA) used to select statistically significant predicators for explaining variation for each data sets used in variation partitioning analyses (Excel file).

Supplementary Table 6. List of specimens and GenBank accession numbers of ITS rDNA used in this study variation partitioning analyses (N=2880). Altitude above sea level, if not given in the publication, was determined based on a given geographic coordinates using the gps converter (www.gps-coordinates.net) (Excel file).

Supplementary Table 7. List of specimens and GenBank accession numbers of ITS rDNA for *Trebouxia* spp. clade A used in this study variation partitioning analyses (N=1080). Altitude above sea level, if not given in the publication, was determined based on a given geographic coordinates using the gps converter (www.gps-coordinates.net) (Excel file).

Supplementary Table 8. List of specimens and GenBank accession numbers of ITS rDNA for *Trebouxia* spp. clade C used in this study variation partitioning analyses (N=347). Altitude above sea level, if not given in the publication, was determined based on a given geographic coordinates using the gps converter (www.gps-coordinates.net) (Excel file).

Supplementary Table 9. List of specimens and GenBank accession numbers of ITS rDNA for *Trebouxia* spp. clade I used in this study variation partitioning analyses (N=378). Altitude above sea level, if not given in the publication, was determined based on a given geographic coordinates using the gps converter (www.gps-coordinates.net) (Excel file).

Supplementary Table 10. List of specimens and GenBank accession numbers of ITS rDNA for *Trebouxia* spp. clade S used in this study variation partitioning analyses (N=1070). Altitude above sea level, if not given in the publication, was determined based on a given geographic coordinates using the gps converter (www.gps-coordinates.net) (Excel file).

Supplementary Table 11. Results of variation partitioning analyses for Bolivian data, all *Trebouxia* dataset, Clade A, C, I and S and selected genera of mycobionts, i.e. *Lecanora* (Lecanoraceae), *Lecidea* (Lecideaceae), *Hypotrachyna*, *Parmotrema*, *Usnea*, *Xanthoparmelia* (Parmeliaceae), *Heterodermia*, *Polyblastidium* (Physciaceae), *Pertusaria* (Pertusariaceae) and *Lepra* (Variolariaceae) (Excel file).

Supplementary Table 12. Secondary metabolites composition detected in selected lichens in respect to their *Trebouxia* sp. OTUs (Excel file).
